# The first record of albanerpetontid amphibians (Amphibia: Albanerpetontidae) from East Asia

**DOI:** 10.1371/journal.pone.0189767

**Published:** 2018-01-03

**Authors:** Ryoko Matsumoto, Susan E. Evans

**Affiliations:** 1 Department of Zoology, Kanagawa Prefectural Museum of Natural History, Odawara, Kanagawa Prefecture, Japan; 2 Department of Cell and Developmental Biology, University College London, London, England; Royal Belgian Institute of Natural Sciences, BELGIUM

## Abstract

Albanerpetontids are an enigmatic fossil amphibian group known from deposits of Middle Jurassic to Pliocene age. The oldest and youngest records are from Europe, but the group appeared in North America in the late Early Cretaceous and radiated there during the Late Cretaceous. Until now, the Asian record has been limited to fragmentary specimens from the Late Cretaceous of Uzbekistan. This led to speculation that albanerpetontids migrated into eastern Asia from North America in the Albian to Cenomanian interval via the Beringian land bridge. However, here we describe albanerpetontid specimens from the Lower Cretaceous Kuwajima Formation of Japan, a record that predates their first known occurrence in North America. One specimen, an association of skull and postcranial bones from a single small individual, permits the diagnosis of a new taxon. High Resolution X-ray Computed Microtomography has revealed previously unrecorded features of albanerpetontid skull morphology in three dimensions, including the presence of a supraoccipital and epipterygoids, neither of which occurs in any known lissamphibian. The placement of this new taxon within the current phylogenetic framework for Albanerpetontidae is complicated by a limited overlap of comparable elements, most notably the non-preservation of the premaxillae in the Japanese taxon. Nonetheless, phylogenetic analysis places the new taxon closer to *Albanerpeton* than to *Anoualerpeton*, *Celtedens*, or *Wesserpeton*, although Bootstrap support values are weak. The results also question the monophyly of *Albanerpeton* as currently defined.

## Introduction

Albanerpetontidae form a distinct and highly derived extinct amphibian clade with a long fossil record. Albanerpetontids share the double occipital condyle of lissamphibians but are characterised by a unique set of skeletal features including: a complex ‘mortise and tenon’ interdentary joint; non-pedicellate, slightly tricuspid, teeth; a sculptured median (fused) frontal; and an amniote-like ‘atlas-axis’ involving three anterior cervical components. Albanerpetontids have been variously considered to be caudates or stem-caudates [1–5], stem-batrachians [6–9], the sister group of Gymnophionomorpha [10–11]; or stem-lissamphibians [5,11–13]. This uncertainty is due partly to differing opinions on the origins and monophyly of Lissamphibia [14], but also to the fragmentary nature of most albanerpetontid material. Where complete specimens exist, from the Early Cretaceous Spanish locality of Las Hoyas [6, 8, 15], they are two-dimensionally compressed, leaving many skull features difficult to interpret. Some three-dimensional skull associations have been described [13, 16], but this material (*Albanerpeton pannonicum*) is fragmentary and comparatively recent (Pliocene), so not necessarily representative of primitive albanerpetontid morphology.

Albanerpetontidae are predominantly Laurasian in distribution [17], the exceptions being specimens from the Middle Jurassic (Bathonian) [18] and Early Cretaceous (Berriasian) [19–20] of Morocco. The longest record for albanerpetontids is in Europe and extends from the Middle Jurassic (Bathonian) of France [21] and England [20, 22–23], to the Pliocene of Hungary [16] and Italy [24], albeit with an unexplained hiatus in the Eocene [25]. In North America, albanerpetontids are first recorded from the uppermost Aptian or lower Albian [26–28] and then remained a consistent element of North American microvertebrate assemblages through to the late Palaeocene [17, 26–27, 29–30]. By contrast, the Asian record of the group is poor and, until now, was restricted to rare and fragmentary material from the Late Cretaceous (Cenomanian/Coniacian) of Uzbekistan, Central Asia [17, 31–33]. The relative ages of the first Asian and American records have influenced discussions as to the direction, timing, and route of possible albanerpetontid dispersal between the two continents [26, 32–34].

Here we extend the record of albanerpetontids in Asia with the description of a new taxon represented by an associated specimen and two isolated dentaries from the Early Cretaceous (Barremian) of Japan. This is the earliest confirmed record from Asia and the first from eastern Asia. Moreover, the three-dimensional preservation of individual elements, as revealed through High Resolution X-ray Computed Microtomography (μCT), provides important new information on albanerpetontid morphology. Given that most recent phylogenetic analyses find albanerpetontids to be related to crown Lissamphibia, a fuller understanding of their morphology has the potential to inform the debate on lissamphibian origins.

## Geological setting

The Mesozoic (Middle Jurassic-Lower Cretaceous) deposits of the Tetori Group are widely distributed in the western part of central Japan, and present a gradual transition from marine to freshwater conditions [35–38]. The Tetori Group is traditionally subdivided into three ascending units: the Kuzuryu Subgroup, dominated by marine deposits; the Itoshiro Subgroup, containing marine and terrestrial deposits; and the Akaiwa Subgroup, consisting mainly of terrestrial sediments [36–37].

**Fig 1:**
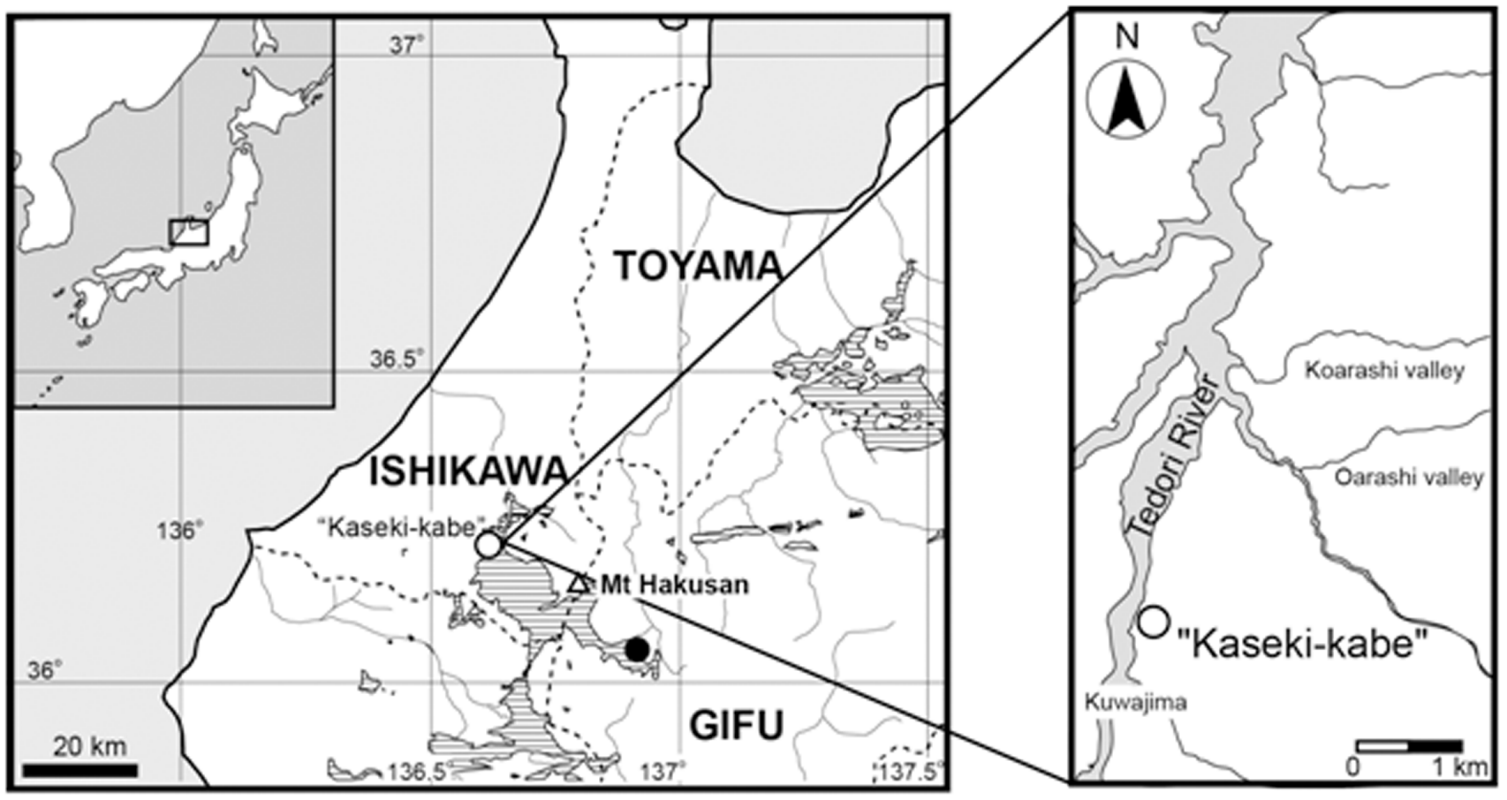
Map of western Japan showing position of the type locality. Areas marked by hashed lines indicate outcrops of the Tetori Group in this region.

The Kuwajima Formation forms the upper part of the Itoshiro Subgroup in the Tetodori River District, and it is mainly composed of non-marine sandstones and mudstones. The albanerpetontid specimens described herein were collected from an outcrop of the Kuwajima Formation forming the Fossil Cliff ("Kaseki-Kabe") in the Kuwajima district, Hakusan City (formerly Shiramine Village), Ishikawa Prefecture (Fig 1). Isajii et al. [39] identified three facies at this locality: Facies I, a carbonaceous swamp; Facies II, a shallow lake; and Facies III, a vegetated swamp. Together, these are thought to represent the wide, stable, vegetated floodplain of a meandering river system, with a humid environment [40–41]. All of these facies have yielded vertebrate remains, including dinosaurs [42–43], pterosaurs [44], mammals [45–46], tritylodonts [47–49], lizards [50–54], fish [55–57], turtles [58], turtle eggshells [59], choristoderes [60–61], and rare frogs [62]. To date, more than 2500 specimens have been recorded from this locality. The greatest abundance of terrestrial taxa has been recovered from Facies III, as were the albanerpetontid specimens described herein.

Most researchers have dated the Kuwajima Formation to the Early Cretaceous, but age estimates have varied from early Neocomian [40] to Hauterivian [38], Valanginian [39], or Berriasian-Hauterivian [42, 60], based on biostratigraphic correlations with other Tetori Group strata. However, zircon U-Pb from a tuff that intruded into the lower part of the Kuwajima Formation gave a date of 130.7 ± 0.8 (2SE) Myr [63], and zircon U-Pb ages of 132 ± 0.9 (2 SE) and 117 ± 0.7 (2 SE) Myr have been reported [64] for the Okurodani Formation in neighbouring Gifu Prefecture, which shares faunal components with the Kuwajima Formation (e.g. the lizard *Sakurasaurus* and the choristodere *Monjurosuchus*). The most recent work [65] dated the Kuwajima Formation to the Barremian, and this is the age estimate we are using herein. The deposit may be older than this but is unlikely to be younger.

## Material and methods

The construction of a road tunnel through the "Kaseki-Kabe" fossil cliff in 1997 yielded almost 17, 000 m^3^ of fossiliferous matrix. Much of this was attributed to the plant rich Facies I, but a sample of 210 m^3^ of Facies II and Facies III was separated out and retained. The matrix is resistant to chemicals and each large block is carefully split into smaller pieces, while examining the exposed surfaces for bone. Any exposed bone is then prepared manually by collections staff and volunteers, with the resulting specimens also being examined periodically by specialists. However, this approach does make it almost impossible to trace the component parts of an original block once it has been broken into smaller pieces. No permits were required for the described study, which complied with all relevant regulations.

The available albanerpetontid material comprises two referred dentaries and a small block of matrix, containing a bone association. All three specimens are held in the Shiramine Institute of Paleontology, Hakusan Board of Education, Hakusan City, Ishikawa Prefecture, Japan (SBEI, Shiramine Board of Education). They are accessible to researchers on application to the curators. Comparative albanerpetontid material from the UK (NHMUK PV R 36956, a parietal) and Spain (MCCM-LH-15710, a partial skeleton) was on loan from the permanent collections of the Natural History Museum, London, and the Museo de las Ciencias de Castilla-La-Mancha, Cuenca, Spain, respectively.

**Fig 2:**
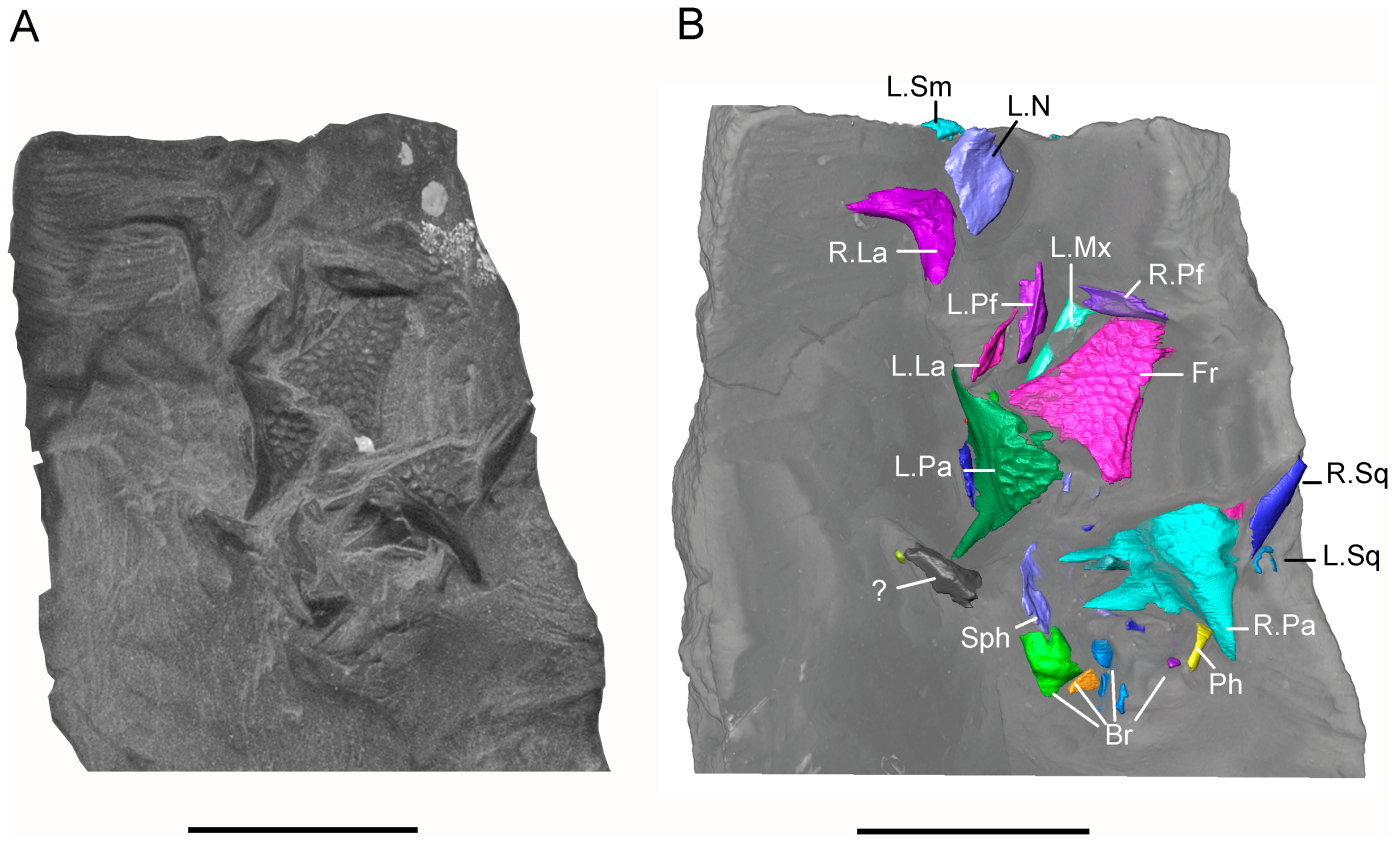
*Shirerpeton isajii* gen. et sp. nov., SBEI 2459, holotype block. A, digital photograph showing surface view of the specimen after manual preparation; B, rendered view of the surface from μCT data showing identifications of exposed elements. Abbreviations: Br, braincase elements; Fr, frontal; L.La, left lacrimal; L.Mx, left maxilla; L.N, left nasal; L.Pa, left parietal; LPf, left prefrontal; L.Sm, left septomaxilla; L.Sq, left squamosal; R.La, right lacrimal; R.Pa, right parietal; R.Pf, right prefrontal; R.Sq, right squamosal;?, unidentified element. Scale bars = 5 mm.

**Fig 3:**
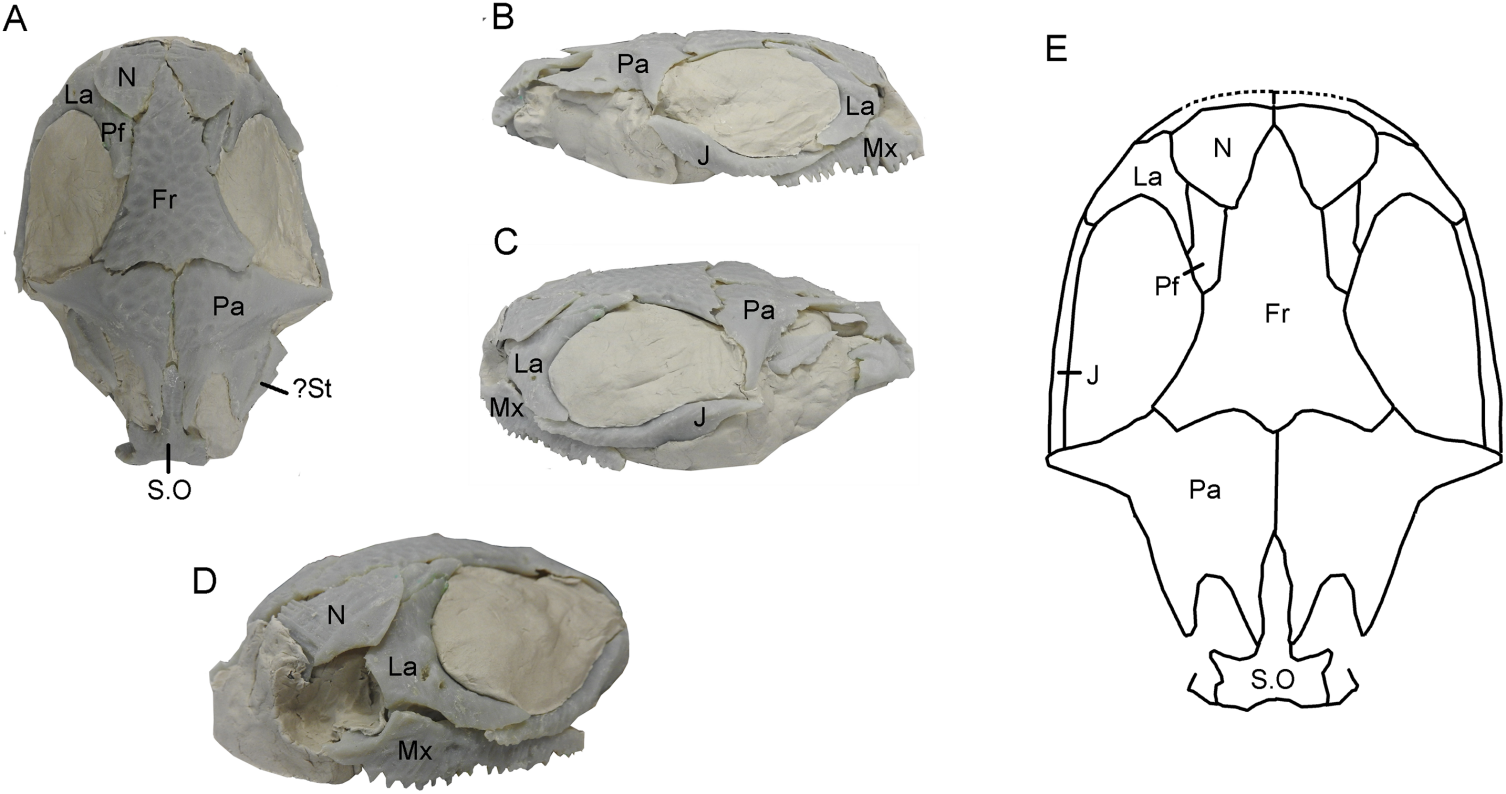
*Shirerpeton isajii* gen. et sp. nov., skull reconstruction. A-D, Model construction. 3-D models constructed using printouts of the individually segmented elements from the μCT data (mirrored as needed: nasal, parietal, possible supratemporal) and fitted into modelling clay. A, dorsal; B, right lateral; C, left lateral; D, left anterolateral showing the relations of the nasal, lacrimal, and maxilla in the narial margin. The tip of the rostrum is roughly reconstructed in modelling clay. E, outline reconstruction of the skull in dorsal view, based on the 3-D model in A-D. Note that the suspensorial elements are omitted as their positions are uncertain. Abbreviations: Fr, frontal; J, jugal; La, lacrimal; Mx, maxilla; N, nasal; Pa, parietal; Pf, prefrontal; S.O, supraoccipital;? St, possible supratemporal.

The matrix block containing the bone association is very small. Initially, only a small area of sculptured bone was exposed on one surface, providing no indication that it originally formed part of a larger specimen, the remains of which cannot be traced. Subsequent manual preparation of the block revealed not only several skull bones on the surface (Fig 2A), but also further underlying bones that could not be accessed without damaging neighbouring elements. Courtesy of the Tokyo Metropolitan Industrial Technology Research Institute, we were able to scan the whole block using a CT scanner (Toscaner 30000 micro CN) at a slice distance of 0.029 mm (100 kV, 30 μA). The braincase region was then rescanned on the same machine at a slice thickness of 0.016 mm (100.0 kV, 30 μA) to achieve greater resolution. One dentary specimen (SBEI 2405) was scanned at the Nagoya Municipal Industrial Research Institute (Toscanner 30000) at a slice width of 0.008 mm (100 kv, 35 μA). A second dentary (SBEI 2462), recovered later, was scanned at the Tokyo Metropolitan Industrial Technology Research Institute at a slice thickness of 0.006 mm (100 kV, 65 μA). Image reconstruction in all cases used AVISO v. 8 (Fig 2B), although the small size of individual elements rendered segmentation of features like foramina, tooth tips, and fine edges difficult. Individual elements segmented out from the scanned block were then 3-D printed (Objet350 Connex) at 20x original size, mirrored where necessary (to provide pairs), and fitted together manually using modelling clay to determine bone positions and articulations in three dimensions (Fig 3A–3D). The physical model generated was then used for the skull reconstruction shown in Fig 3E.

### Nomenclatural acts

The electronic edition of this article conforms to the requirements of the amended International Code of Zoological Nomenclature, and hence the new names contained herein are available under that Code from the electronic edition of this article. This published work and the nomenclatural acts it contains have been registered in ZooBank, the online registration system for the ICZN. The ZooBank LSIDs (Life Science Identifiers) can be resolved and the associated information viewed through any standard web browser by appending the LSID to the prefix “http://zoobank.org/”. The LSID for this publication is: urn:lsid:zoobank.org:pub: C8490EBE-E927-4CD6-BEA6-BEFE1A71DF77. The electronic edition of this work was published in a journal with an ISSN, and has been archived and is available from the following digital repositories: PubMed Central, LOCKSS, UCL Discovery Publications database.

### Note on nomenclature

Marjanović and Laurin [11] noted that as *erpeton* is neuter, a number of original species names have a grammatically incorrect ending e.g. *Anoualerpeton unicus* [20] should be *An*. *unicum*. We have therefore used the corrected species spelling throughout this manuscript.

## Systematic palaeontology

Lissamphibia [66]

Albanerpetontidae [12]

### *Shirerpeton* gen. nov.

urn: lsid: zoobank.org: act:C3726120-0EF5-4407-8624-05A4C0B28EFD

### Etymology

From the Japanese Shiro, white, partly for Shiramine, the type locality, but also because the family name, Albanerpetontidae, derives from the original French locality of La Grive-Saint-Alban, with Alba/Alban (Latin) meaning white.

### Type species

*Shirerpeton isajii* sp. nov.

urn:lsid:zoobank.org:act:86783E12-F44E-4F12-8C96-81327BA47DB6

#### Diagnosis

As for type and only species

### *Shirerpeton isajii* sp. nov.

#### Holotype

Shiramine Board of Education Ishikawa Prefecture, SBEI 2459, a small block bearing most of a disarticulated but associated skull with some postcranial elements (Fig 2A). The specimen is housed in the Shiramine Institute of Paleontology, Hakusan Board of Education, Hakusan City, Ishikawa Prefecture, Japan.

#### Etymology

Species name honours Dr Shinji Isaji, Chiba Prefecture Museum, Japan, for his longstanding work on the fossils, geology, and palaeoenvironment of the Kuwajima Formation.

#### Locality and horizon

"Kaseki-Kabe" (fossil cliff), Kuwajima, Hakusan City (formerly Shiramine Village), Ishikawa Prefecture, Honshu, Japan. Lower Cretaceous, (Barremian), Kuwajima Formation, Tetori Group. Both the holotype and the referred specimen came from Facies III, the more terrestrial of the three facies at the type locality.

#### Differential diagnosis

A genus of albanerpetontid that resembles *Albanerpeton* spp. and *Wesserpeton evansae* [34], and differs from *Anoualerpeton* spp. [20] and *Celtedens ibericus* [6], in having a frontal that is triangular rather than bell-shaped; differs from all but *Albanerpeton arthridion* [12], in that the frontal body is short but anteriorly quite wide, but differs from *A*. *arthridion* in having a longer, more pointed internasal process; resembles *Wesserpeton* and *A*. *arthridion* in its very small body size, but the frontal differs from that of *Wesserpeton* in the more tapered internasal process, the anterior contact between the ventrolateral crests, and the fact that the posterior margins of the prefrontal facets lie posterior to the mid-length of the bone; differs from *Wesserpeton*, *Albanerpeton inexpectatum* [3], *Celtedens ibericus* [6], and *Anoualerpeton priscum* [20], in having parietals with proportionally longer postorbital wings, the dorsal surfaces of which remain completely unsculptured; further differs from *A*. *inexpectatum* in lacking fusion of the prefrontal and lacrimal, having a bifurcate occipital shelf of each parietal, and in having a nasal that enters the narial margin. In the latter feature it resembles *A*. *pannonicum* [16], but differs in that the nasal makes a larger contribution to the narial margin; differs from both Neogene taxa in the lack of fusion of the basicranial and otic elements, the discrete supraoccipital, and the long anterodorsal process of the latter bone. The dentary and maxilla resemble *Anoualerpeton* spp. and differ from *Wesserpeton*, *Celtedens*, unattributed material from the Cretaceous of Uzbekistan (‘*Nukusurus*’), and *Albanerpeton* (except *A*. *nexuosum* [2]) in showing size heterodonty with large anterior teeth supported by a convex profile of the labial alveolar margin. However, the maxilla of *Shirerpeton* is distinguished from that of *Anoualerpeton* spp and *A*. *nexuosum* by the combination of a pointed (rather than rounded) anterior premaxillary process and a weakly concave anterior narial margin. The dentary of *Shirerpeton* is distinguished from that of *Anoualerpeton* spp and *A*. *nexuosum* by the greater sinuosity of the labial dental margin (with a concave-convex-concave profile), the positioning of the prearticular facet behind the tooth row (rather than extending forward beneath it), and the shallow posterior inclination of the subdental shelf such that the posterior teeth are not markedly smaller than those in the symphyseal region. Note that direct comparison with the type species of *Celtedens*, *C*. *megacephalus* [67], is not possible as the holotype specimen (Instituto Geologico dell’Universitá de Napoli, Italy, IGUN M542: the anterior part of a skeleton) does not show comparable features.

#### Referred specimens

**Fig 4:**
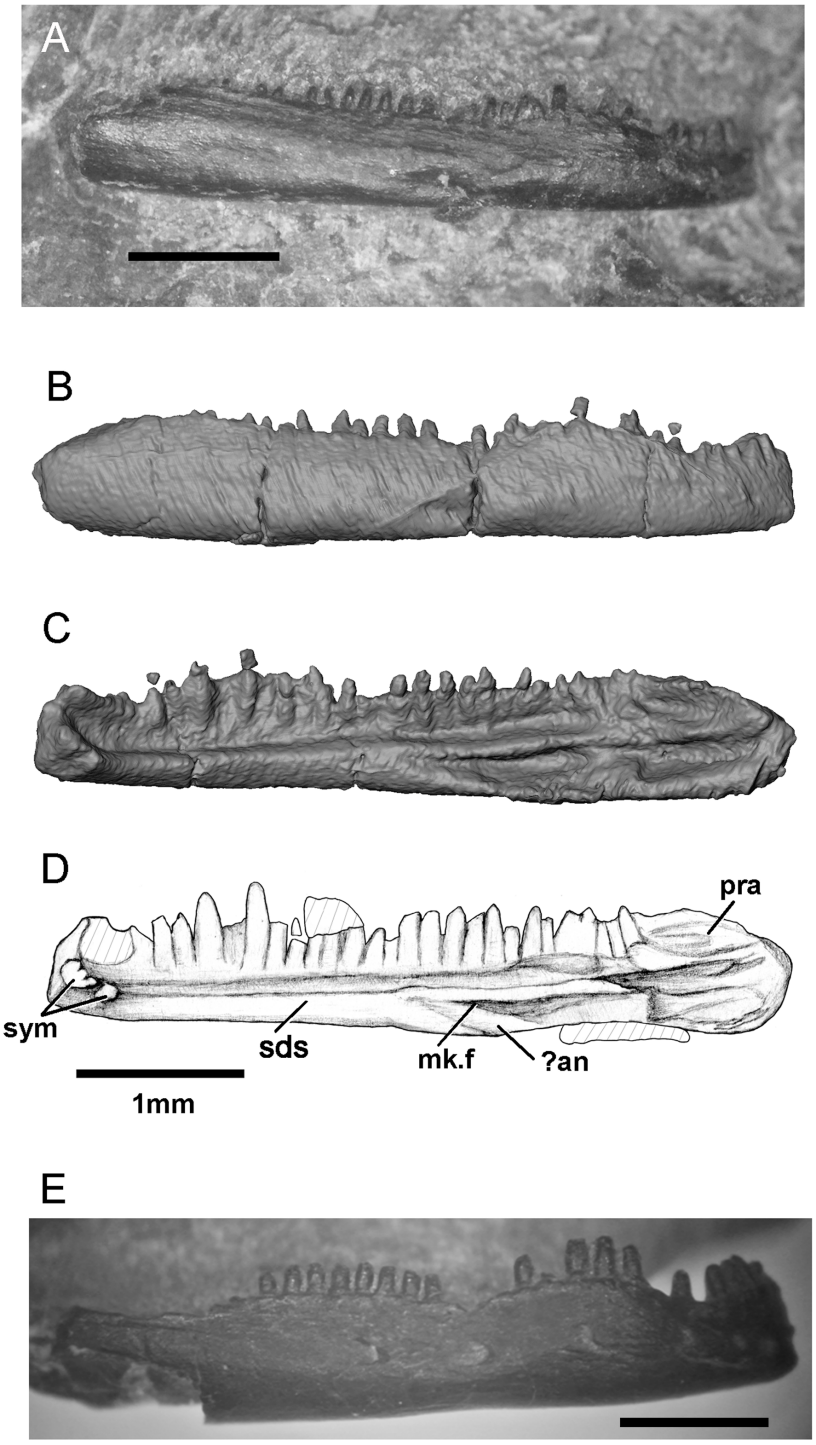
*Shirerpeton isajii* gen. et sp. nov., referred right dentaries. A-D, SBEI 2405, **in** A, labial view as preserved, digital photograph; B-C, specimen as segmented from μCT slice data in B, labial, and C, lingual views; D, lingual view, interpretative drawing. E, SBEI 2462, labial view as preserved, digital photograph. Abbreviations:? an, possible angular facet; mk.f, Meckelian fossa; pra, facet for prearticular; sds, subdental shelf; sym, symphyseal prongs. Scale bars = 1 mm.

SBEI 2405, an almost complete right dentary (Fig 4A–4D); and SBEI 2462 (Fig 4E), a second right dentary, both from the type locality.

### Anatomical description

**Fig 5:**
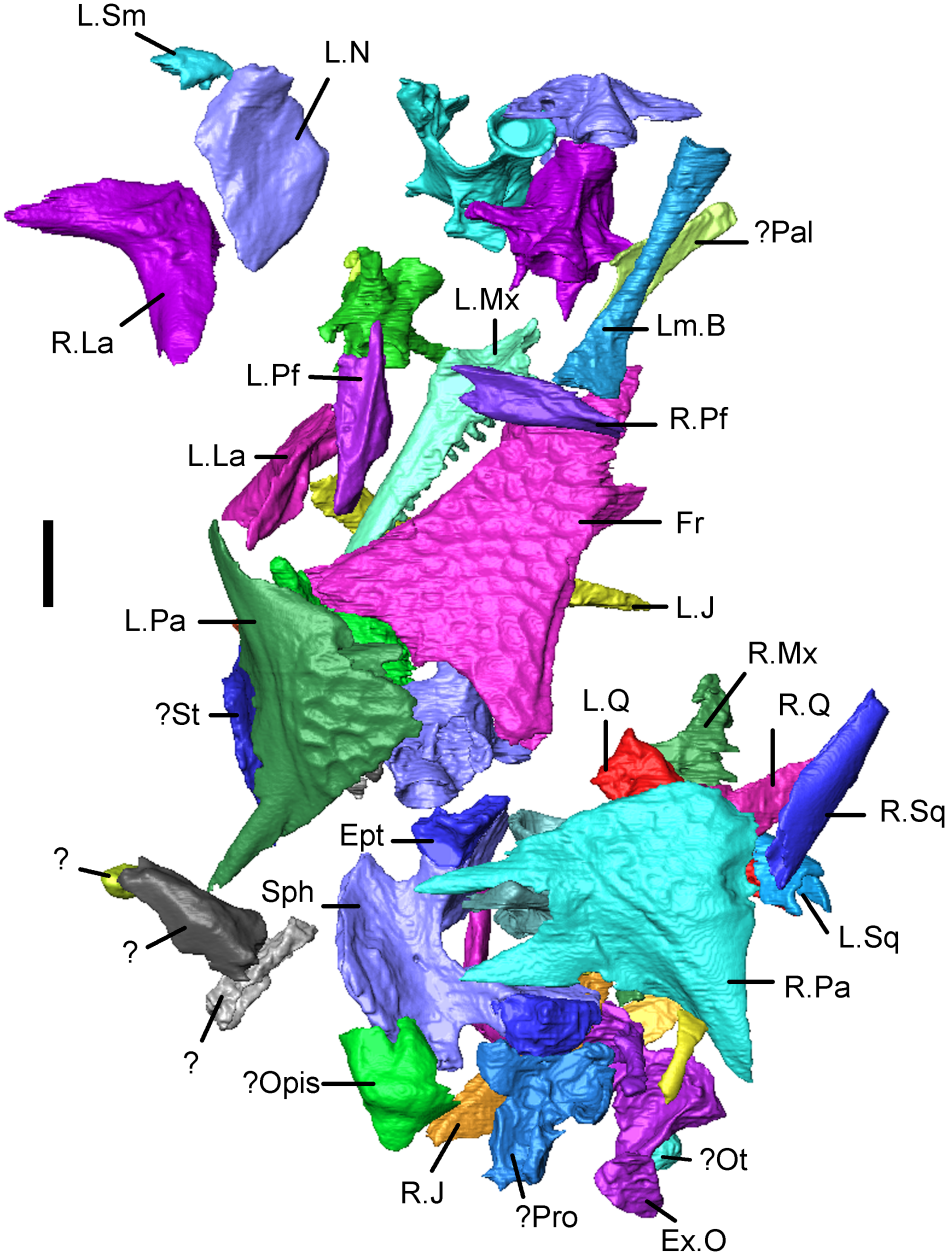
*Shirerpeton isajii* gen. et sp. nov., SBEI 2459, holotype block in dorsal view showing elements segmented from μCT slice data. Abbreviations: Ept, epipterygoid; Ex.O, exoccipital plate; Fr, frontal; L.J, left jugal; L.La, left lacrimal; Lm.B, limb element; L.Mx, left maxilla; L.N, left nasal; L.Pa, left parietal; LPf, left prefrontal; L.Q, left quadrate; L.Sm, left septomaxilla; L.Sq, left squamosal;? Opis, possible opisthotic;? Ot, otic associated element;? Pal, possible palatal element;? Pro, possible prootic; R.J, right jugal; R.La, right lacrimal; R.Mx, right maxilla; R.Pa, right parietal; R.Pf, right prefrontal; R.Q, right quadrate; R.Sq, right squamosal; Sph, sphenoid;? St, possible supratemporal;?, unidentified elements. Scale bar = 1 mm. Note that the vertebrae are not labelled in this figure but are described and figured later in the text.

**Fig 6:**
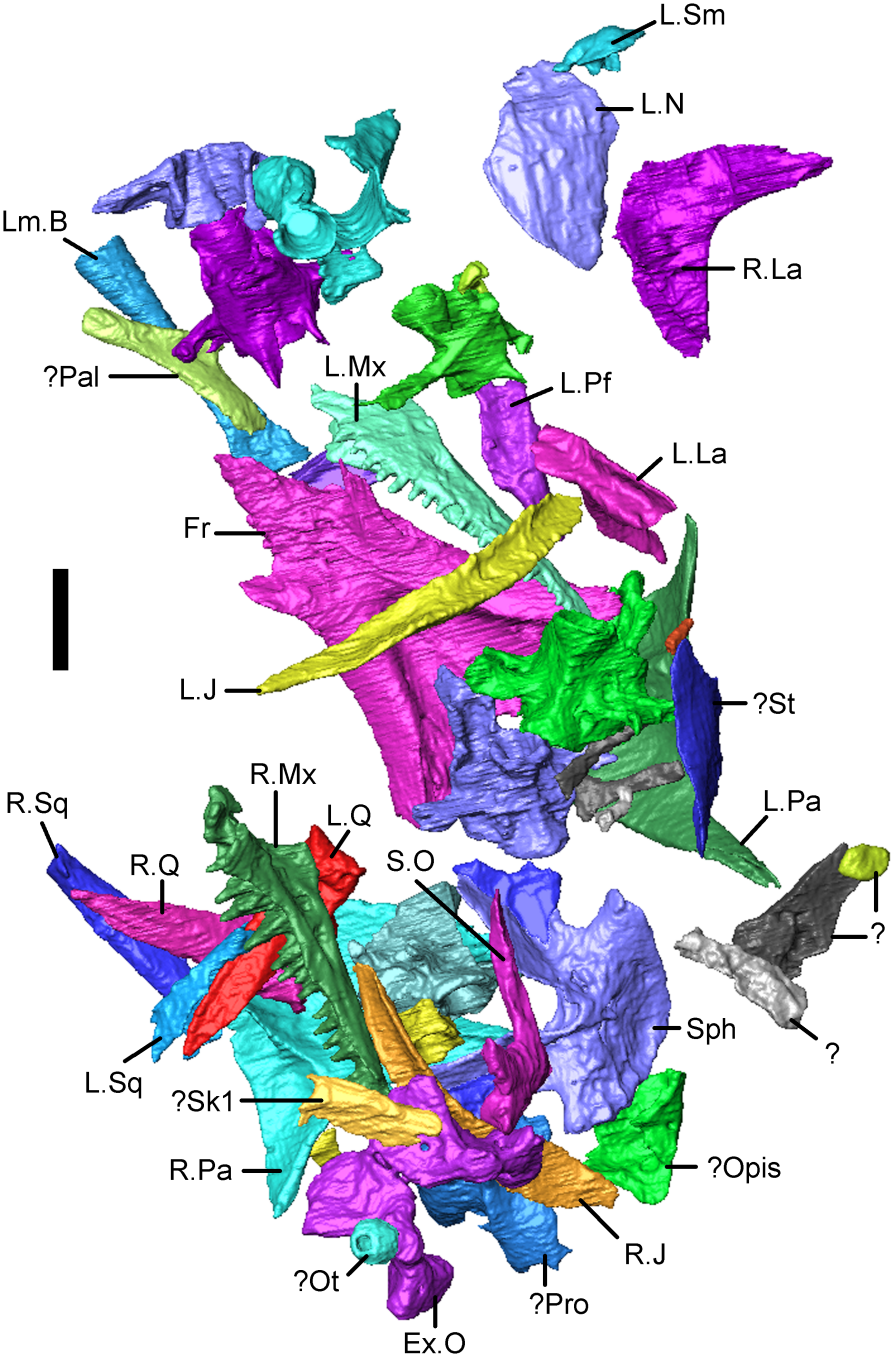
*Shirerpeton isajii* gen. et sp. nov., SBEI 2459, holotype block in ventral view showing elements segmented from μCT slice data. Abbreviations: Ex.O, exoccipital plate; Fr, frontal; L.J, left jugal; L.La, left lacrimal; Lm.B, limb element; L.Mx, left maxilla; L.N, left nasal; L.Pa, left parietal; L.Pf, left prefrontal; L.Q, left quadrate; L.Sm, left septomaxilla; L.Sq, left squamosal;? St, possible left supratemporal;? Opis, possible opisthotic;? Ot, otic associated element;? Pal, possible palatal element;? Pro, possible prootic; R.J, right jugal; R.La, right lacrimal; R.Mx, right maxilla; R.Pa, right parietal; R.Q, right quadrate; R.Sq, right squamosal; S.O, supraoccipital;?Sk1, unidentified skull element; Sph, sphenoid;? St, possible supratemporal;?, unidentified elements. Scale bar = 1 mm. Note that the vertebrae are not labelled in this figure but are described and figured later in the text.

**Fig 7:**
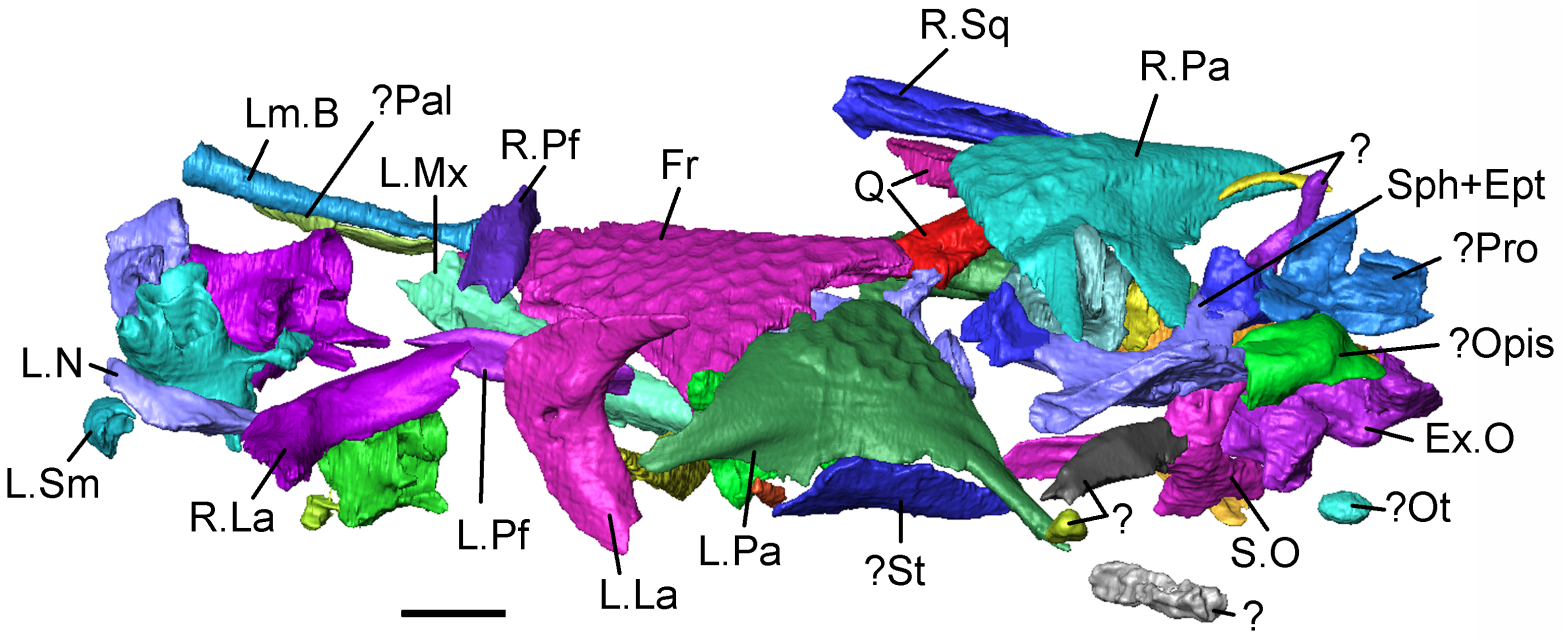
*Shirerpeton isajii* gen. et sp. nov., SBEI 2459, holotype block. Lateral view of skull elements as segmented from μCT slice data. Abbreviations: Ex.O, exoccipital plate; Fr, frontal; L.La, left lacrimal; Lm.B, limb element; L.Mx, left maxilla; L.N, left nasal; L.Pa, left parietal; LPf, left prefrontal; Q, quadrates; L.Sm, left septomaxilla;? Opis, possible opisthotic;? Ot, otic associated element;? Pal, possible palatine;? Pro, possible prootic; R.La, right lacrimal; R.Pa, right parietal; R.Pf, right prefrontal; R.Sq, right squamosal; S.O, supraoccipital; Sph+ Ept, sphenoid and epipterygoid;? St, possible supratemporal;?, unidentified elements. Scale bar = 1 mm. Note that the vertebrae are not labelled in this figure but are described and figured later in the text.

The holotype of *Shirerpeton isajii*, SBEI 2459 (Fig 2), is a small block of grey mudstone bearing three-dimensionally preserved but partly disarticulated skull and postcranial elements. From the anatomical relationships of the bones, lack of duplication, and equivalent size, the remains clearly pertain to a single individual. The reconstructed midline skull length was 8-10mm, with an estimated snout-pelvis length (SPL) of roughly 45 mm (based Gardner [26], with SPL approximately 10x frontal length). As exposed on the surface of the block (Fig 2A), the most distinctive elements are a fused frontal bearing the raised polygonal sculpture typical of albanerpetontids, and paired sculptured parietals. Despite the small size of the bones, the sculpture is of strong relief. Displaced to the left of the frontal are several other bones including the left and right lacrimal, the left prefrontal, and the left maxilla preserved in partial dorsal view. The right prefrontal, crosses the anterior end of the frontal. Anterior to the prefrontal is the left nasal, inverted but complete. Posterior to the frontal are the left and right parietals. An anteriorly directed, rod-like element adjacent to the edge of the right parietal is interpreted as the displaced squamosal of that side. There are further partially exposed elements posterior to the parietals. These were difficult to interpret in surface view but were subsequently found to be vertebrae and parts of the braincase. The surface view has been supplemented by the μCT data which has revealed important details of the exposed bones as well as those of elements that are fully or partially embedded within the matrix (Figs 5–7). These additional bones include the right maxilla, both septomaxillae, quadrates, epipterygoids, and jugals, as well as many parts of the endocranium, several vertebrae, a few limb elements, and several additional elements, some of which have a distinctive structure but have yet to be identified. Unfortunately there is no trace of the premaxillae on the holotype block, nor the lower jaws, and these parts of the block were probably removed inadvertently during trimming of a larger block.

#### Skull

**Fig 8:**
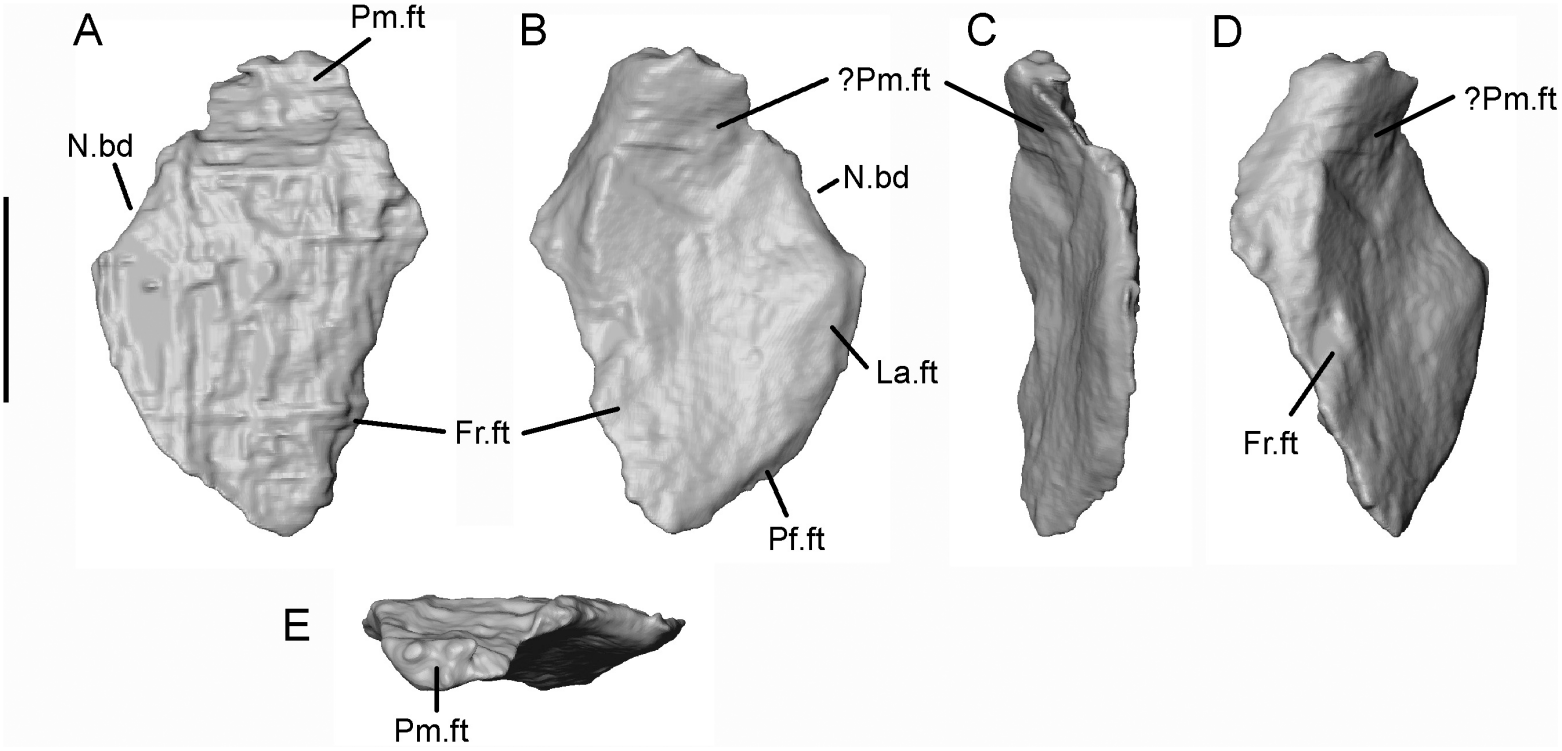
*Shirerpeton isajii* gen. et sp. nov., SBEI 2459, nasal. Left nasal as segmented from μCT slice data in A, dorsal; B, ventral; C, medial; D, lateral; and E, anterior views. Abbreviations: Fr.ft, frontal facet; La.ft, lacrimal facet; N.bd, narial border; Pf.ft, prefrontal facet; Pm.ft, anterodorsal facet for nasal process of premaxilla;? Pm.ft, possible anteroventral facet for the premaxilla. Scale bar = 1 mm.

There are few published accounts of the albanerpetontid nasal [3,8], leading to a degree of uncertainty about its presence, size, and relations in most taxa. Venczel and Gardner [16] described the first three-dimensionally preserved albanerpetontid nasal in the Pliocene *Albanerpeton pannonicum*, revealing that it was larger than previously estimated [3]. Mechanical preparation of SBEI 2459 exposed a single complete nasal in ventral view, and further details have been revealed by the μCT data (Fig 8). Based on its morphology and fit with the frontal, we interpret the bone as a left nasal. It is roughly rhomboidal. The posterior tip is tapered whereas the anterior tip is blunt and thickened. The facetted posteromedial edge fitted against the internasal process of the frontal. The complementary edges (of nasal and frontal) are of similar length, suggesting that the nasals met only, at most, for a short distance at their anteromedial angle. The posterolateral edge of the nasal bears a narrower surface that contacted the medial edges of the prefrontal and the lacrimal. Our reconstruction demonstrates that the nasal-lacrimal contact excluded the prefrontal from the narial margin. Of the two anterior nasal borders, the thin unfacetted anterolateral edge clearly entered the narial opening. The anteromedial edge is thicker, inflected ventrally, and slightly buttressed. It supports a dorsal facet for the nasal process of the premaxilla (Pm.ft). The thickened edge is squared-off and bears several rugosities. In other albanerpetontid taxa, the naso-premaxillary contact is usually described as being an abutment and/or a variably developed suture in which the premaxilla overlaps the anterior margin of the nasal [16]. The Japanese specimen appears to combine these features, with the premaxilla overlapping the nasal, and the tip of the nasal abutting against a ridge or tuberosity on the underside of the premaxillary nasal process. However, a second anteroventral facet on the Japanese nasal suggests the articulation may have been more complex, perhaps with a pocket facet on the premaxilla (?Pm.ft). Without the premaxilla, however, we cannot speculate further.

**Fig 9:**
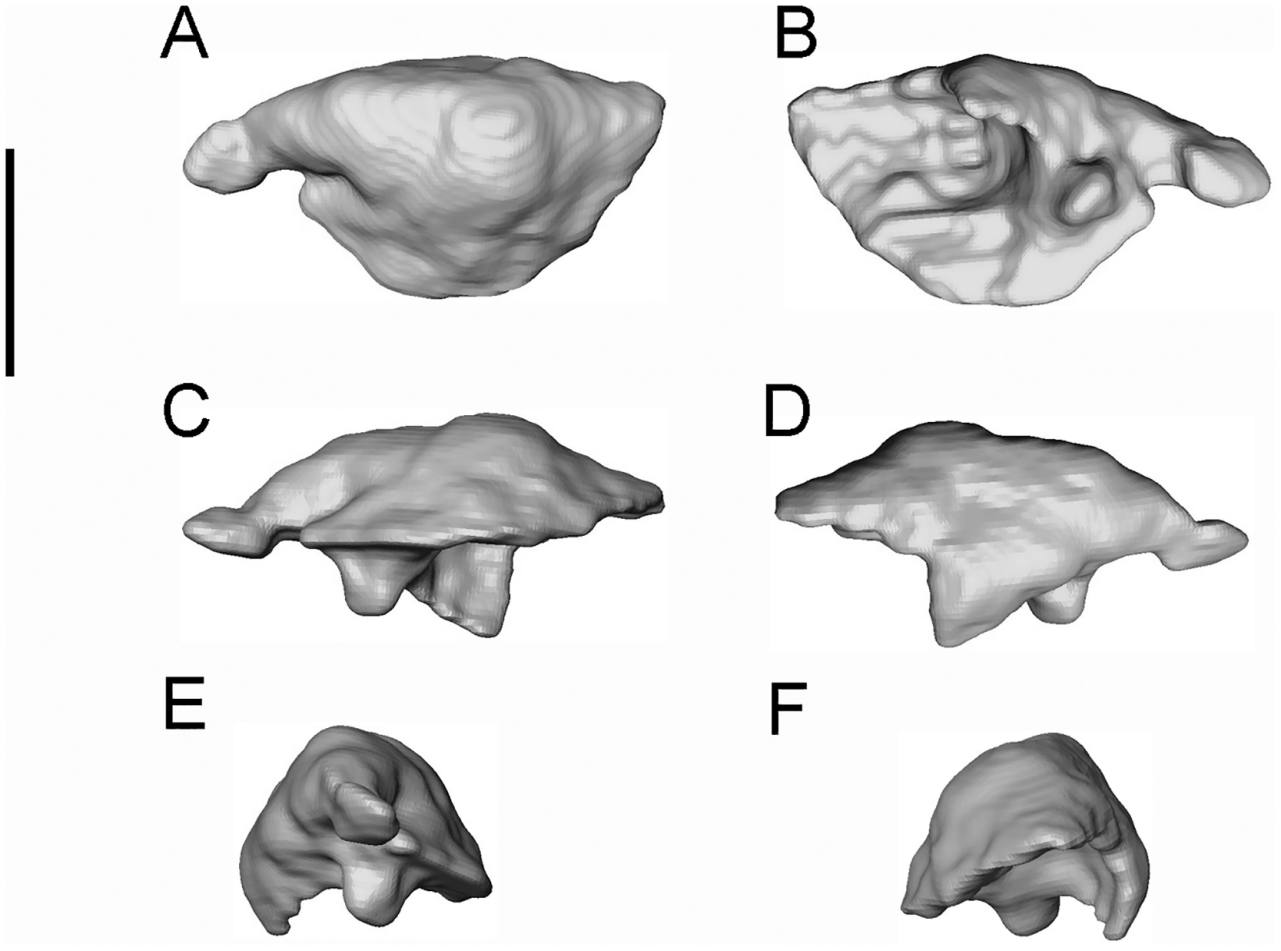
*Shirerpeton isajii* gen. et sp. nov., SBEI 2459, septomaxilla. Left septomaxilla as segmented from μCT slice data in A, dorsal; B, ventral; C, posterior; D, anterior; E, lateral; and F, medial views. Scale bar = 0.5 mm.

The μCT scans revealed the presence of a very small element lying adjacent to the anteromedial edge of the nasal. This has a smooth, rounded, hemispherical external surface and an internal surface divided between a deep concavity and a rugose portion that tapers to a point. It appears to be a single complete element rather than a broken part of a larger bone (Fig 9). From its position in relation to the nasal, and its convex-concave shape, a septomaxilla is the most plausible identification. This element, associated with the nasal capsule, is present in at least some representatives of both extinct and extant amphibian lineages [68, 69], and therefore its presence in albanerpetontids would not be unexpected.

**Fig 10:**
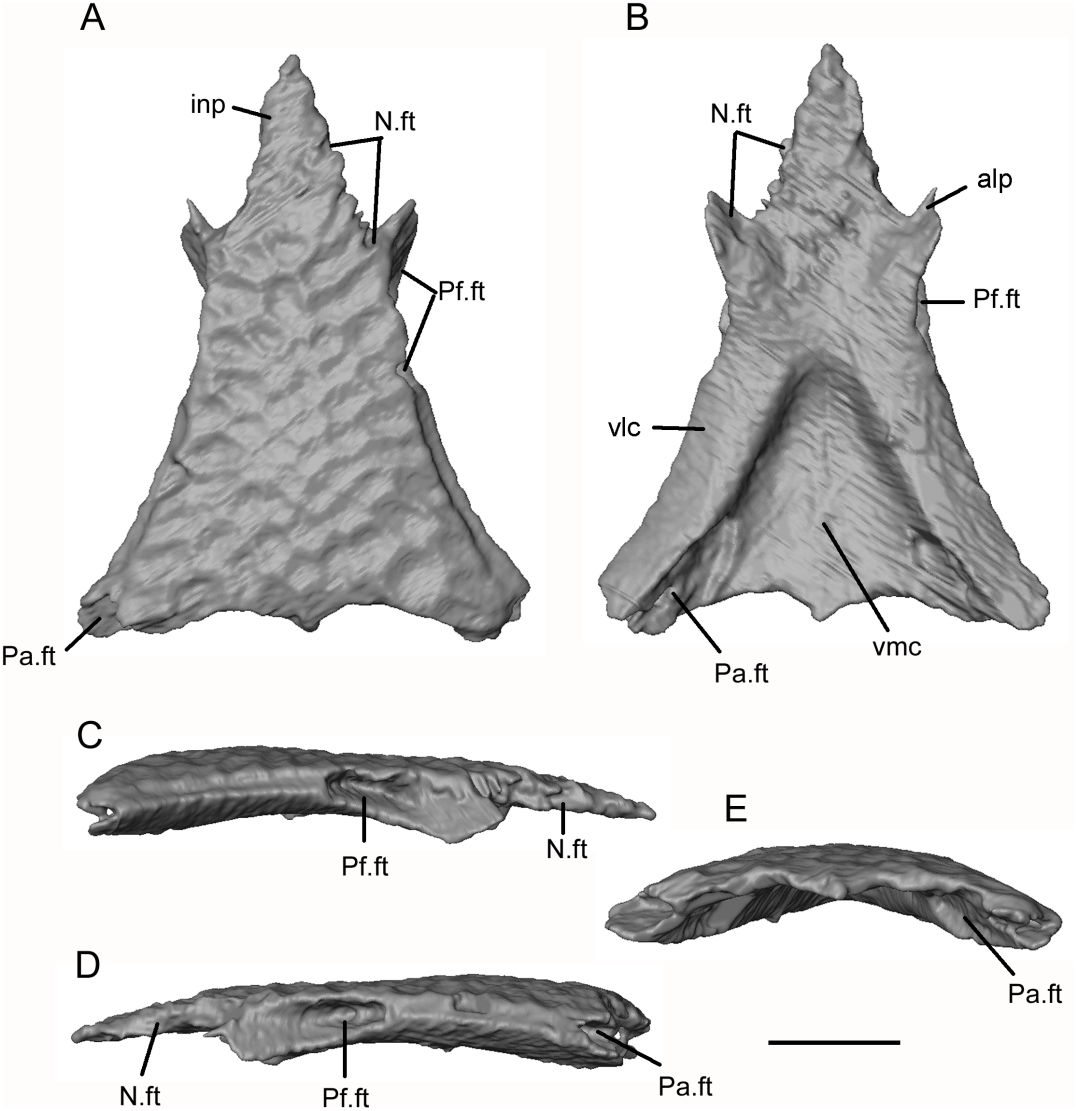
*Shirerpeton isajii* gen. et sp. nov., SBEI 2459, frontal. Frontal as segmented from μCT slice data in A, dorsal; B, ventral; C, right lateral; D, left lateral; and E, posterior views. Abbreviations: alp, anterolateral process; inp, internasal process; N.ft, nasal facet; Pa.ft, parietal facet; Pf.ft, prefrontal facet; vlc, ventrolateral crest; vmc, ventromedian crest. Scale bar = 1 mm.

The frontal is well preserved. In surface view the internasal process is obscured by the overlying right prefrontal (Figs 2 and 5), but this has been digitally removed. As reconstructed from μCT data (Fig 10), the frontal has a midline length of *c*. 4.5 mm and a posterior width of *c*. 3.7 mm, giving a midline length to posterior width proportion of 1.21. The internasal process is relatively long and tapering (length = 1.26x basal width), with the edge recessed in its posterior half by the nasal facet. Further anteriorly, the naso-frontal articulation appears to have been more of an abutment but allowance must be made for possible artefacts introduced during the segmentation of these very small bones. Small anterolateral processes separate the nasal facets from the prefrontal facets. However, these processes do not appear to have reached the level of the dorsal surface, so that the prefrontal and nasal would have been in contact in a dorsal view of the skull. The anterior end of the orbital margin (demarcated by the posterior end of the slot for reception of the prefrontal) lies posterior to the mid-point of the frontal long axis. The lateral edges of the bone are only slightly concave, but the dorsal surface of the bone (Fig 10A) is slightly narrower than the ventral one (Fig 10B), so that a narrow gutter runs along the edge from the posterior margin of the prefrontal facet to the posterolateral tip of the frontal (Fig 10C and 10D). Here there is a small dorsal slot facet for the parietal (more completely preserved on the left than the right). The posterior margin of the frontal (Fig 10A, 10B and 10E) is weakly W-shaped, with shallow bilateral emarginations flanking a short posteromedian process.

The ventral surface of the frontal has been rendered digitally (Fig 10B). The surfaces of the ventrolateral crests are weakly concave. The crests are widest at the posterior margin of the prefrontal facet and then narrow slightly toward the frontoparietal suture. In the posterior two-thirds of the bone, the crests are separated by a deep concavity, but this is closed off further anteriorly where the ventrolateral crests are joined in the midline. There is a weak ventromedian crest. Seen in lateral view, the prefrontal facets are deep and extend along the full length of the lateral surface of the anterolateral processes. As noted above, the nasal facets are shallower. At the posterodorsal corner of the frontal (preserved more completely on the left than the right, Fig 10A), the frontal bears a small facet for the parietal. This is continuous with a larger ventral facet that extends medial to the ventrolateral crest. The posterolateral corner of the frontal thus slots into a recess in the anterior face of the parietal.

**Fig 11:**
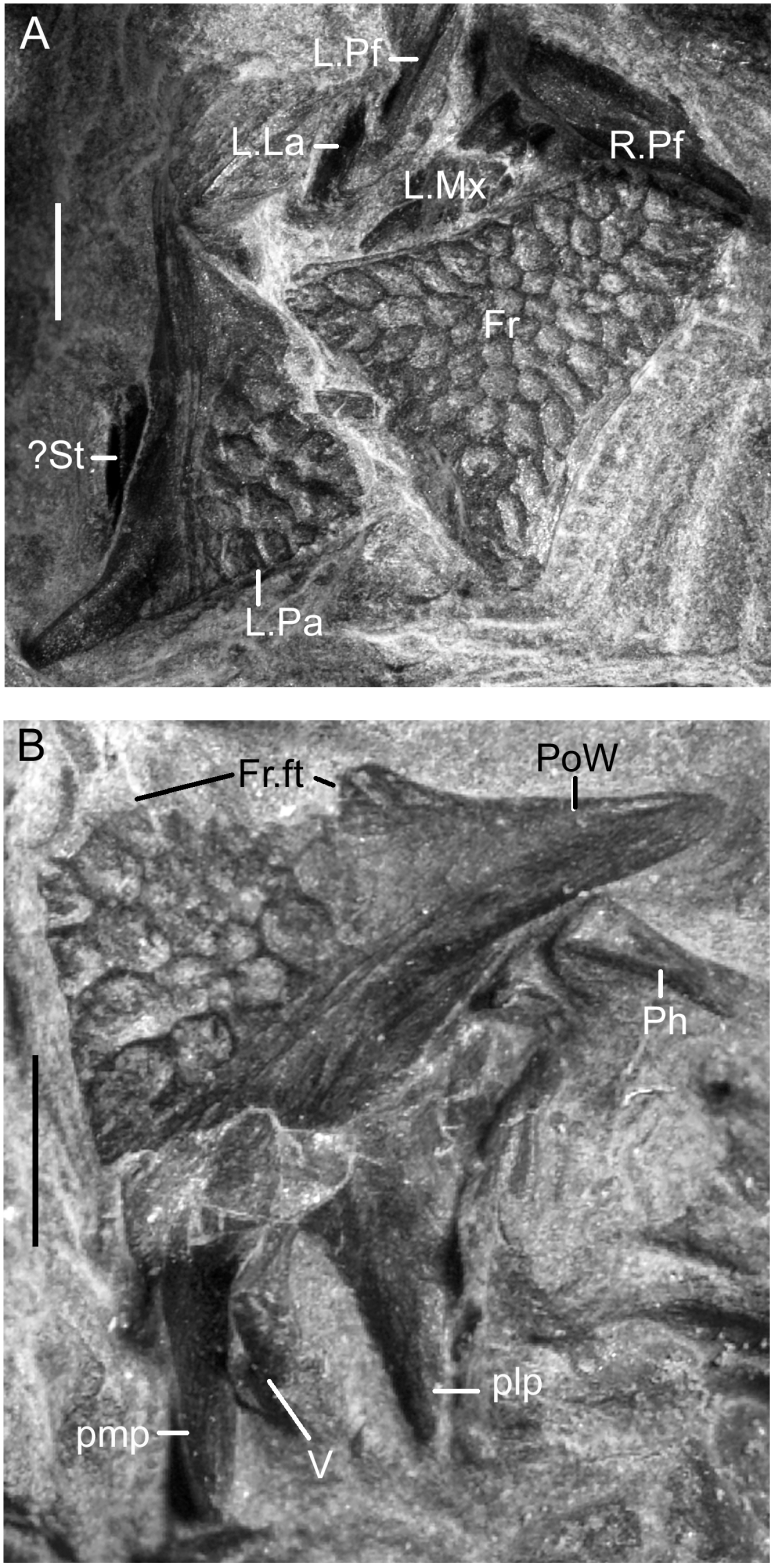
*Shirerpeton isajii* gen. et sp. nov, SBEI 2459, digital photograph showing details of skull roofing elements. A, frontal and left parietal; B, right parietal. Abbreviations: Fr, frontal; Fr,ft, frontal facet; L.La, left lacrimal; L.Mx, left maxilla; L.Pa, left parietal; L.Pf, left prefrontal; plp, posterolateral process; pmp, posteromedial process; PoW, postorbital wing; R.Pf, right prefrontal;? St, possible supratemporal; V, vertebra. Scale bars = 1 mm.

**Fig 12:**
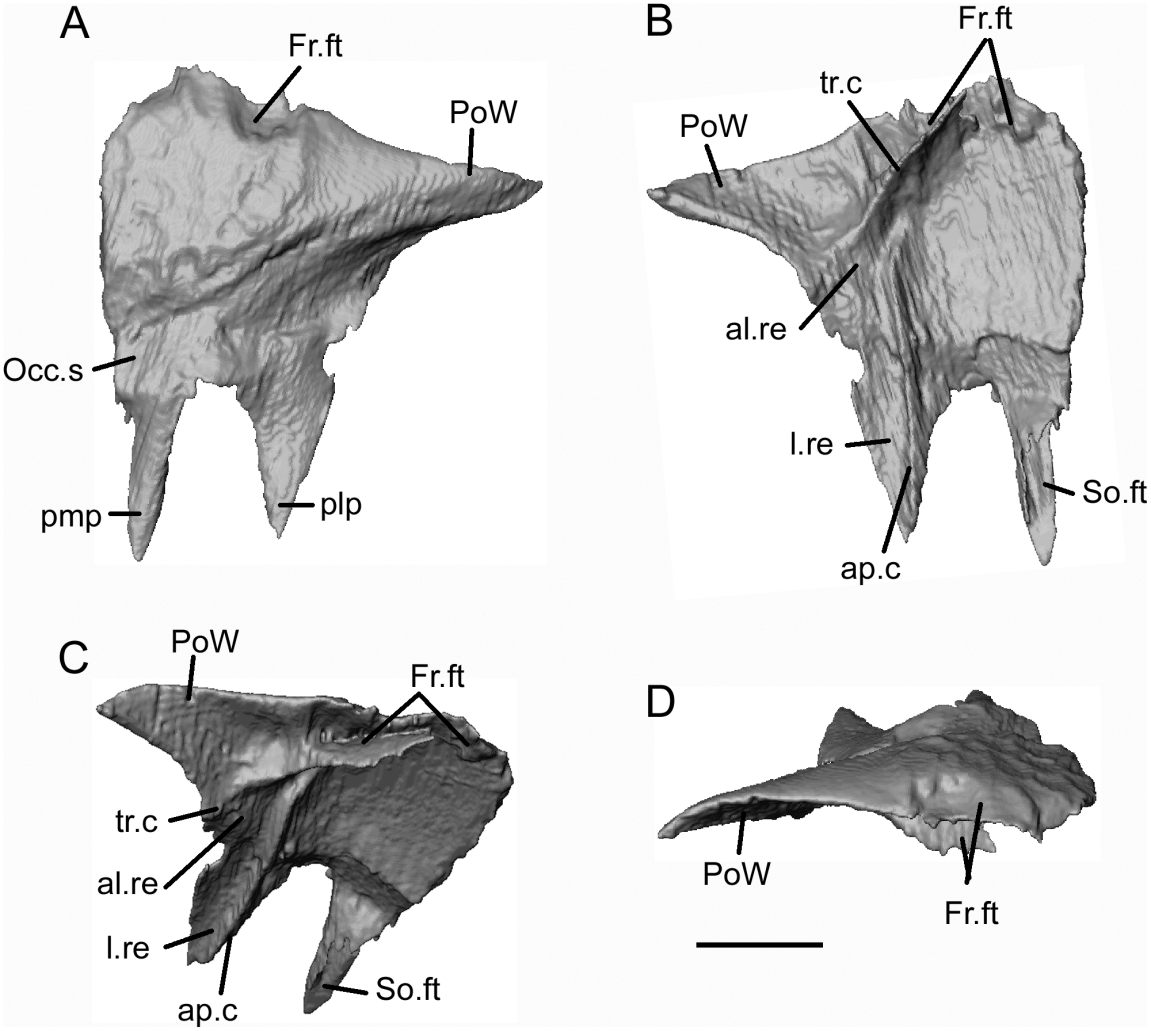
*Shirerpeton isajii* gen. et sp. nov., SBEI 2459, right parietal. Right parietal as segmented from μCT slice data in A, dorsal; B, ventral; C, oblique anteroventral; D, anterior views. Abbreviations: al.re, anterolateral recess; ap.c, anteroposterior crest; Fr.ft, frontal facet; l.re, lateral recess; Occ.s, occipital shelf; plp, posterolateral process; pmp, posteromedial process; PoW, postorbital wing; SO.ft, supraoccipital facet; tr.c, transverse crest; Scale bar = 1 mm.

**Fig 13:**
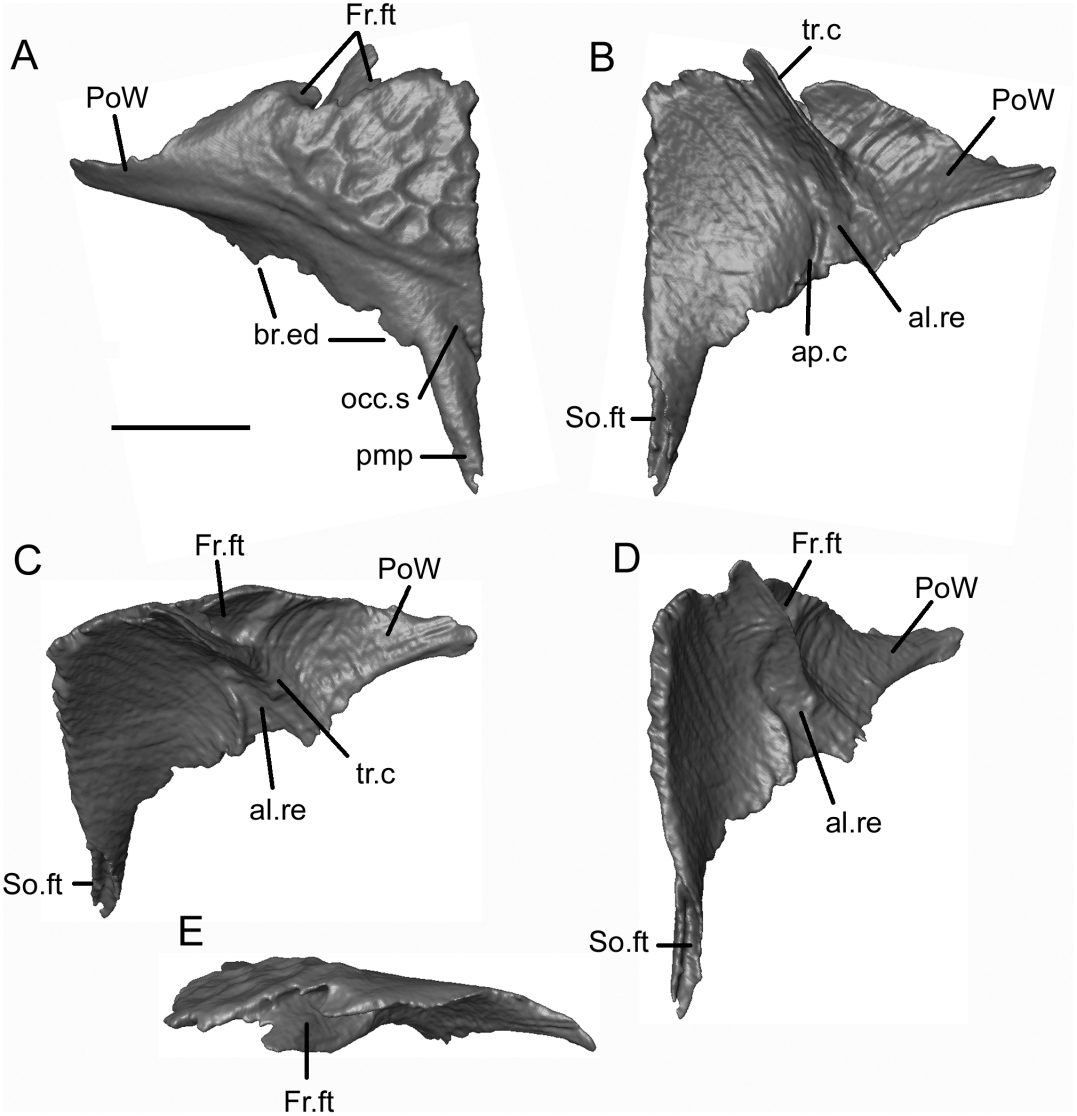
*Shirerpeton isajii* gen. et sp. nov., SBEI 2459, left parietal. Left parietal as segmented from μCT slice data in A, dorsal; B, ventral; C, anteroventral; D, ventromedial; and E, anterior views. Abbreviations: al.re, anterolateral recess; ap.c, anteroposterior crest; br.ed, broken edge; Fr.ft, frontal facet; occ.s, occipital shelf; pmp, posteromedial process; PoW, postorbital wing; So.ft, supraoccipital facet; tr.c, transverse crest. Scale bar = 1 mm.

Albanerpetontid parietals have rarely been described and, to date, the only nearly complete bones that have been figured are those of the Miocene *Albanerpeton inexpectatum* [3] and the Early Cretaceous *Celtedens ibericus* [6, 8]. The parietals are well preserved in the Tetori specimen. The left bone is roughly in situ relative to the frontal (Figs 5, 6 and 11), but the right has been rotated clockwise so that its anterior margin faces laterally (Fig 2). Most of the important details of the dorsal surface can be seen on the original block (Fig 11), but the description is supplemented by images from μCT scans which also allow visualisation of the ventral surface (Figs 12 and 13).

Each parietal is roughly triangular. The lateral margin of the parietal is curved and extends from the lateral tip of the postorbital process to the tip of the occipital shelf. The anterior margin is smooth along the tapering postorbital process, then irregular where it bears the frontal facet. The μCT scan images reveal a deep pocket (Fr.ft) that received the posterolateral tip of the frontal (Fig 12C). Anteroventral and anterolateral flanges match the facets on the corresponding surfaces of the frontal, contributing to a firm articulation. The central part of the frontoparietal suture and the straight interparietal suture appear weaker with only small interdigitations. However, the medial edge of the occipital shelf bears an incised slot facet for the anterior ramus of the supraoccipital (Figs 12B, 12C and 13B–13D).

The dorsal surface of the parietal (Figs 12A and 13A) is subdivided into three areas: a sculptured triangular anteromedial surface that forms the posterior part of the skull table; an unsculptured lateral postorbital wing; and an unsculptured occipital shelf to which neck muscles presumably attached. On the right parietal the shelf is extensive, as in *A*. *inexpectatum*, but it differs in being bifurcated. This does not appear to be an artefact of breakage (although a vertebral transverse process projects between the two rami) or crushing, as the edges bordering the emargination appear intact, and the completely preserved posteromedial processes on both parietals match one another in shape and size. On the left parietal the more lateral of the posterior processes has broken off at its base, as is clear from a comparison of the ventral surfaces of the right and left bones. On both elements, a sharp anteromedial to posterolateral transverse crest is seen to divide the main, medial, part of the bone from the recessed postorbital wing (Figs 12B and 13B). A second anteroposterior crest runs smoothly from the midpoint of the transverse crest to the tip of the posterolateral process. On the left parietal, this second crest is truncated posteriorly, marking where the posterolateral process has broken away. On the right parietal, the area lateral to the second crest is seen to bear a lateral recess, with a more oblique anterolateral recess towards its anterior end. Estes and Hoffstetter’s image of the parietal in *Albanerpeton inexpectatum* ([3] plate 8) shows a similar lateral recess, which the authors interpreted as accommodating the dorsal surface of the otic capsule. However, the larger lateral recess in *Shirerpeton* more closely resembles a facet for a dermal element, possibly a supratemporal (see below), whereas the more anterior recess matches the size and position of the dorsal end of an element that we interpret as the epipterygoid (see below). The strongly concave anteroventral surface of the postorbital wing presumably provided an area of origin for jaw adductor muscles (possibly the deep part of the internal adductor).

As reconstructed, therefore, the occipital shelves appear to have been deeply emarginated in the Japanese taxon. With the supraoccipital in articulation between the posteromedial parietal processes, this creates large ovoid openings in the skull roof on each side of the midline, medial to the otic capsules (Fig 3E). If genuine (i.e. not an artefact), these openings could have been covered by a thick sheet of fascia or an additional dermal element such as a postparietal.

**Fig 14:**
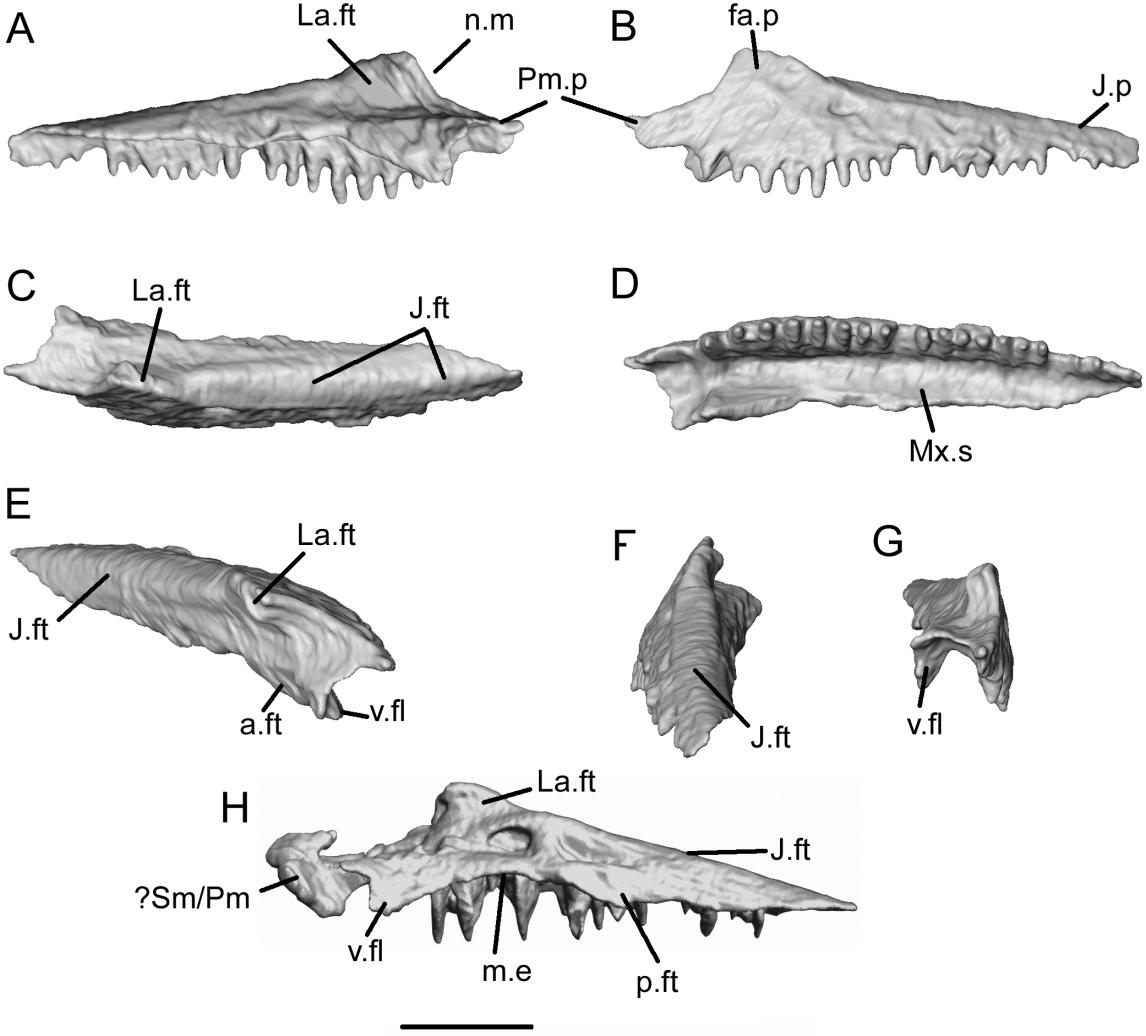
*Shirerpeton isajii* gen. et sp. nov., SBEI 2459, maxilla. A-G, left maxilla as segmented from μCT slice data, in A, lingual; B, labial; C, dorsal; D, ventral; E, oblique anterior; F, oblique posterior; G, anterior views. H, right maxilla in lingual view. Abbreviations: a.ft, anterior facet; fa.p, facial process; J.ft, jugal facet; J.p, jugal process; La.ft, lacrimal facet; m.e, medial emargination; Mx.s, maxillary shelf; n.m, narial margin; p.ft, posterior facet; Pm.p, premaxillary process;? Sm/Pm, bone fragment, part of premaxilla or septomaxilla; v.fl, ventral flange; Scale bar = 1 mm.

Both maxillae are preserved, with the left roughly in situ and the right lying under the right parietal (Figs 5 and 6). The maxilla has the shape of a shallow scalene triangle (Fig 14A and 14B), with a tapering premaxillary process, a low rounded facial process, and a long tapering jugal process (Fig 14A–14F and 14H). The anterior, narial, margin of the facial process is almost straight (rather than strongly concave as in some other taxa). Allowing for artefacts in segmentation and some damage, the labial surface of the maxilla appears unsculptured, but at least two neurovascular foramina open on this surface. The premaxillary process is bifurcated (Fig 14C–14E and 14G). The medial ramus bears a ventral flange that is almost vertical in orientation on the left bone (Fig 14G), but ventromedial on the right. It appears to bear a facet, either for the premaxilla or the vomer. The posterodorsal edge of the facial process is recessed both labially and lingually where it was straddled by the lacrimal bone (Fig 14B–14E and 14H). Further posteriorly, the dorsomedial surface bears a slightly flattened surface that supported the jugal. Lacrimal and jugal facets meet, indicating that the maxilla was excluded from the ventral orbital margin, and this is confirmed when the bones are rearticulated (Fig 3A–3D). A medial view of the right maxilla (Fig 14H) also shows a distinct embayment in the lingual edge of the maxillary shelf posterior to the medial premaxillary process. We interpret this as the lateral margin of the choana. It is less obvious on the left due to damage. Posterior to the embayment, the edge of the shelf is straighter and bears a facet for a palatal element. A large medial foramen perforates the posterior base of the right facial process and probably carried neurovascular structures into the bone. There is a second, more posterior, excavation but we cannot determine whether this also opened as a foramen. Anteriorly, the maxillary tooth row reaches the bifurcation of the premaxillary process, a point anterior to the narial margin of the facial process. Allowing for empty positions, the tooth row seems to accommodate 18–22 teeth, those below the facial process being longer than those at the anterior and posterior ends of the tooth row. This heterodonty is also reflected by the downward curvature of the ventrolateral edge of the maxilla which is deepest level with the apex of the facial process (Fig 14A and 14B). At its anterior end, the right maxilla is in contact with a fragment of another bone. This could be part of a right septomaxilla or premaxilla (Fig 14H).

**Fig 15:**
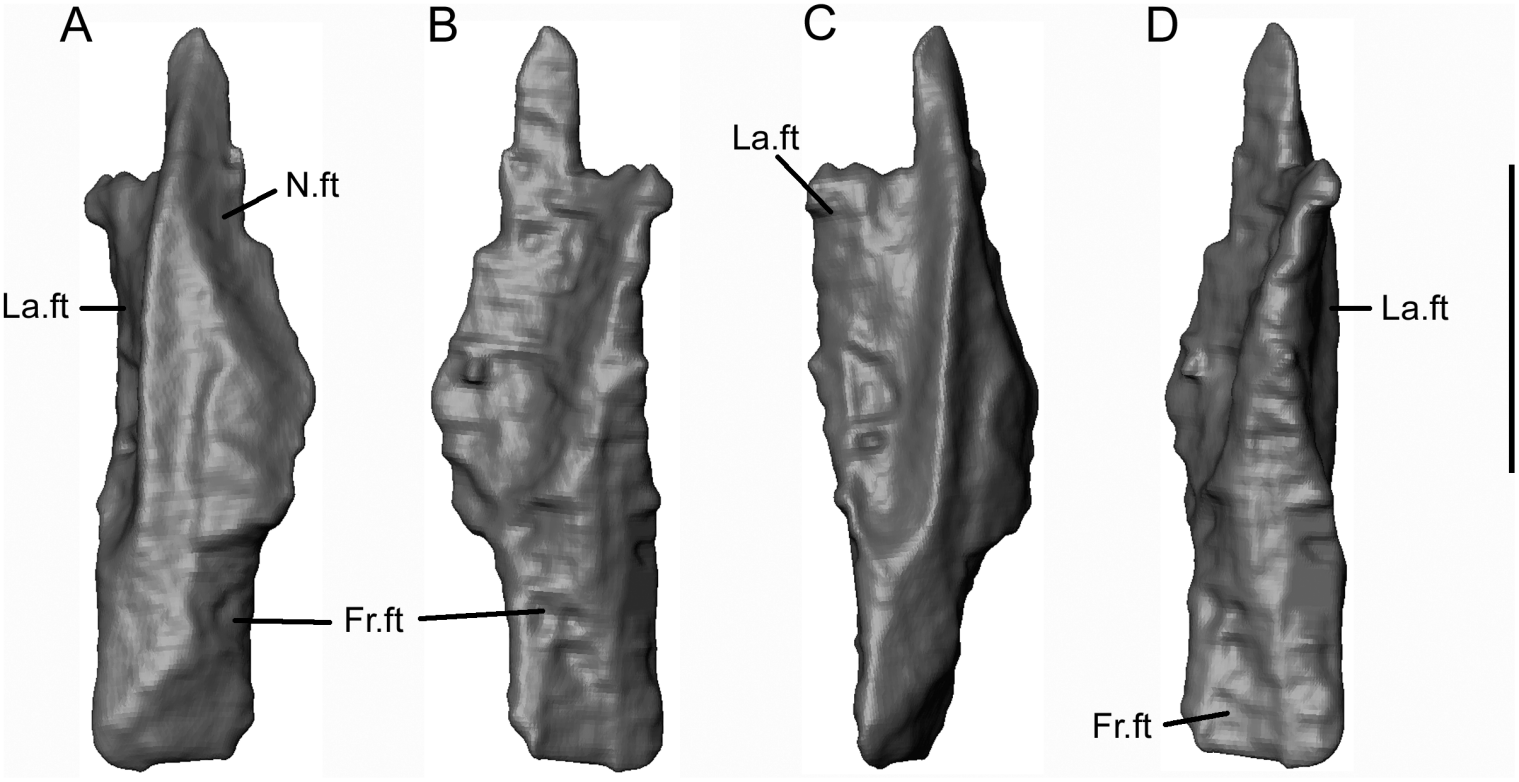
*Shirerpeton isajii* gen. et sp. nov., SBEI 2459, prefrontal. Left prefrontal as segmented from μCT slice data in A, dorsal; B, ventral; C, lateral; and D, medial views. Abbreviations: Fr.ft, frontal facet; La.ft, lacrimal facet; N.ft, nasal facet. Scale bar = 1 mm.

The left prefrontal lies lateral to the frontal (Figs 2 and 5–7). It is a slender bone with narrow medial shelf facets for the nasal and frontal, and a lateral, or ventrolateral, lacrimal facet visible on its exposed margin (Fig 15). The medial and lateral facets converge anteriorly and, at the tip of the bone, are separated only by a low ridge. Aligning this element to the edge of the frontal indicates that the posterior part of the medial facet met the frontal, but the more horizontal anteromedial part was overlapped by the nasal. The nasal and lacrimal met anterior to the prefrontal and excluded it from the narial margin (Fig 3E). The right prefrontal lies across the anterior tip of the frontal and has the same morphology as the left.

**Fig 16:**
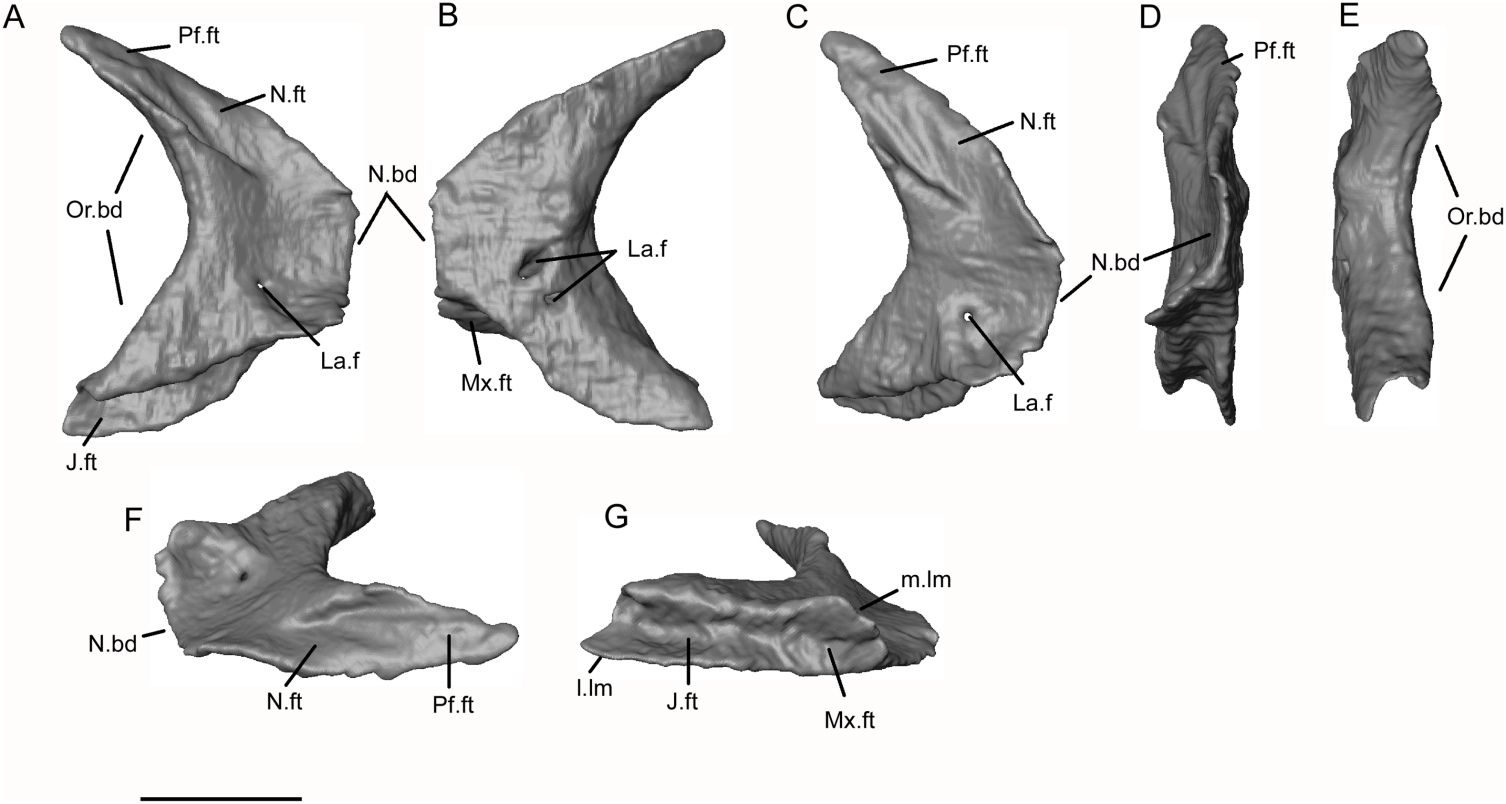
*Shirerpeton isajii* gen. et sp. nov., SBEI 2459, lacrimal. Left lacrimal as segmented from μCT slice data in A, medial; B, lateral; C, anterodorsomedial; D, anterior; E, posterior; F, dorsal; and G, ventral views. Abbreviations: J.ft, jugal facet; La.f, lacrimal duct foramen; l.lm, lateral lamina; m.lm, medial lamina; Mx.ft, maxillary facet; N.bd, narial border; N.ft, nasal facet; Or.bd, orbital border; Pf.ft, prefrontal facet; Scale bar = 1 mm.

The dorsal edge of the left lacrimal, bearing prefrontal and nasal facets, is exposed lateral to the maxilla and left prefrontal. The remainder of the bone is buried almost vertically in matrix (Figs 5–7). The right lacrimal is fully exposed in lateral view on the matrix block (Fig 2). Both bones are biradiate with tapering ventral and anterodorsal edges processes that lie almost at right angles to one another. Between them, the long, curved, posterior border framed the orbit, whereas the shorter, almost vertical, anterior edge contributed to the narial border. The extracted left lacrimal (Fig 16) resembles the right but reveals rather more detail. Just anterior to the orbital margin, the ventrolateral surface is perforated by two lacrimal duct foramina (Fig 16B). These run anteromedially into a short canal that opens on to the medial surface through a single foramen (Fig 16A and 16C). The lateral foramina are visible only as indentations on the exposed right bone, but they lie in the same position as those on the lateral surface of the right bone.

The medial surface of the lacrimal is exposed most clearly on the left bone, with dorsal and ventral facets separated by a short body (Fig 16A and 16C). The body is divided into a thin, medially concave anterior part that walled part of the nasal chamber (Fig 16D) and a thickened posterior orbital margin (Fig 16E). The single lacrimal duct foramen opens into the posterior edge of the nasal concavity. The dorsomedial border of the bone bears a large tapering facet for the prefrontal. Anterior to this facet, the smooth bone surface changes slightly in its orientation and may represent the point at which the lacrimal contacted the nasal (Fig 16A, 16C and 16F). The ventral margin of the bone bears a deep groove divided into anterior and posterior articular surfaces for the maxilla and jugal respectively. The ventral groove is flanked by medial and lateral laminae (Fig 16G). However, whereas the medial lamina is deepest anteriorly, the lateral lamina is deeper posteriorly. This arrangement ensures that the lacrimal, maxilla, and jugal are locked. The ventral groove accommodates the nasal process of the maxilla anteriorly, leaving this process exposed in lateral view but braced medially by the medial lacrimal lamina, and the jugal posteriorly, with the lateral lacrimal lamina bracing the jugo-maxillary contact. Overall, the lacrimal of the Japanese albanerpetontid is broadly similar to that figured for both *Albanerpeton inexpectatum* [3] and *A*. *pannonicum* [16], although the detailed shape (lateral recess, dorsal and ventral process dimensions) is different in each taxon. The reported fusion of the prefrontal to the lacrimal in *A*. *inexpectatum* [3, 7] obscures the dorsal relationship between these elements.

**Fig 17:**
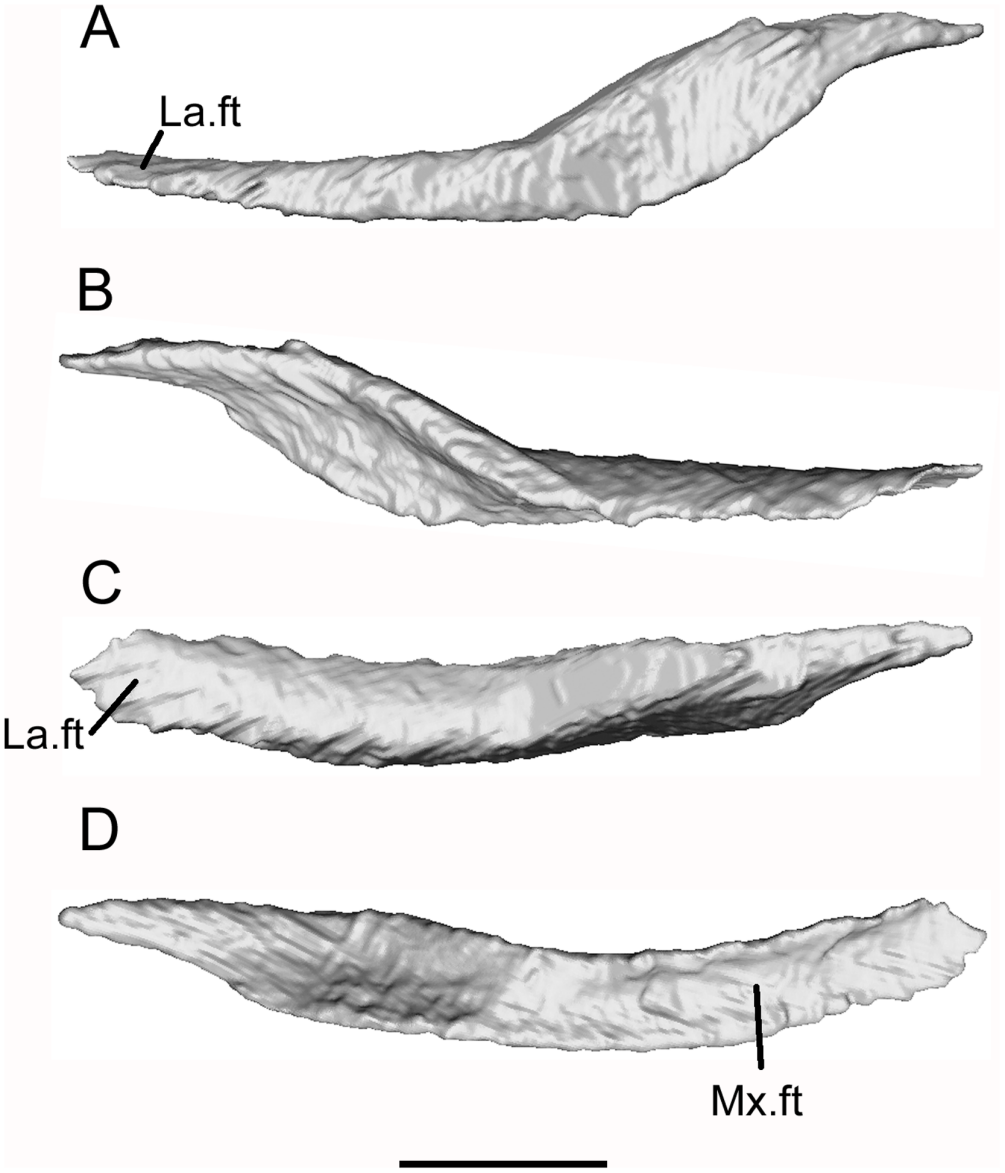
*Shirerpeton isajii* gen. et sp. nov., SBEI 2459, jugal. Left jugal as segmented from μCT slice data in A, lateral; B, medial; C, dorsal; and D, ventral views. Abbreviations: La.ft, lacrimal facet; Mx.ft, maxillary facet. Scale bar = 1 mm.

Unlike extant lissamphibians, albanerpetontids are known to have retained a jugal [6, 8,16] but dorsal reconstructions of the skull show it as a sliver of bone running between the maxilla and suspensorium. McGowan [8] reconstructed the jugal as overlapping the lateral surface of the squamosal, but this has not been confirmed from three-dimensional material of the squamosal. Venczel and Gardner [16] described several partial jugals of *Albanerpeton pannonicum* in association with the maxilla and lacrimal. Their most complete specimen is shown as extending only a short distance beyond the end of the maxillary tooth row, with its slightly expanded posterior edge bearing both medial and lateral facets. This would require that the tip slotted into a recess on the squamosal or was wedged between the squamosal and quadrate. However, in the Tetori specimen, the scans have revealed complete left and right jugals, the former lying under the frontal and the latter lying within the matrix in association with the right maxilla (Figs 2, 5, 6 and 17). The bone has the form of a curved bar, divided into a shallow anterior section and a vertical posterior blade. The anterior portion is tapered at its tip and consists of narrow lateral and medial flanges that straddled the dorsal surface of the maxilla. Dorsally, the anterior end bears a short facet for the lacrimal which overlapped both maxilla and jugal at this point. The posterior half of the bone is deeper in its mid section, but also tapers to a point posterodorsally, as seen on the left bone. Precisely how the jugal articulated with the suspensorium, if at all, is uncertain, as we could find no evidence of an articular surface on the posteroventral tip of the bone (as recorded for *A*. *pannonicum* [16]). From the reconstruction, it appears likely that the posterodorsal tip of the jugal approached the lateral tip of the parietal postorbital wing. However, there are no obvious facets on the postorbital wing or the tip of the jugal, and the latter may have been connected to the skull roof by a ligament.

**Fig 18:**
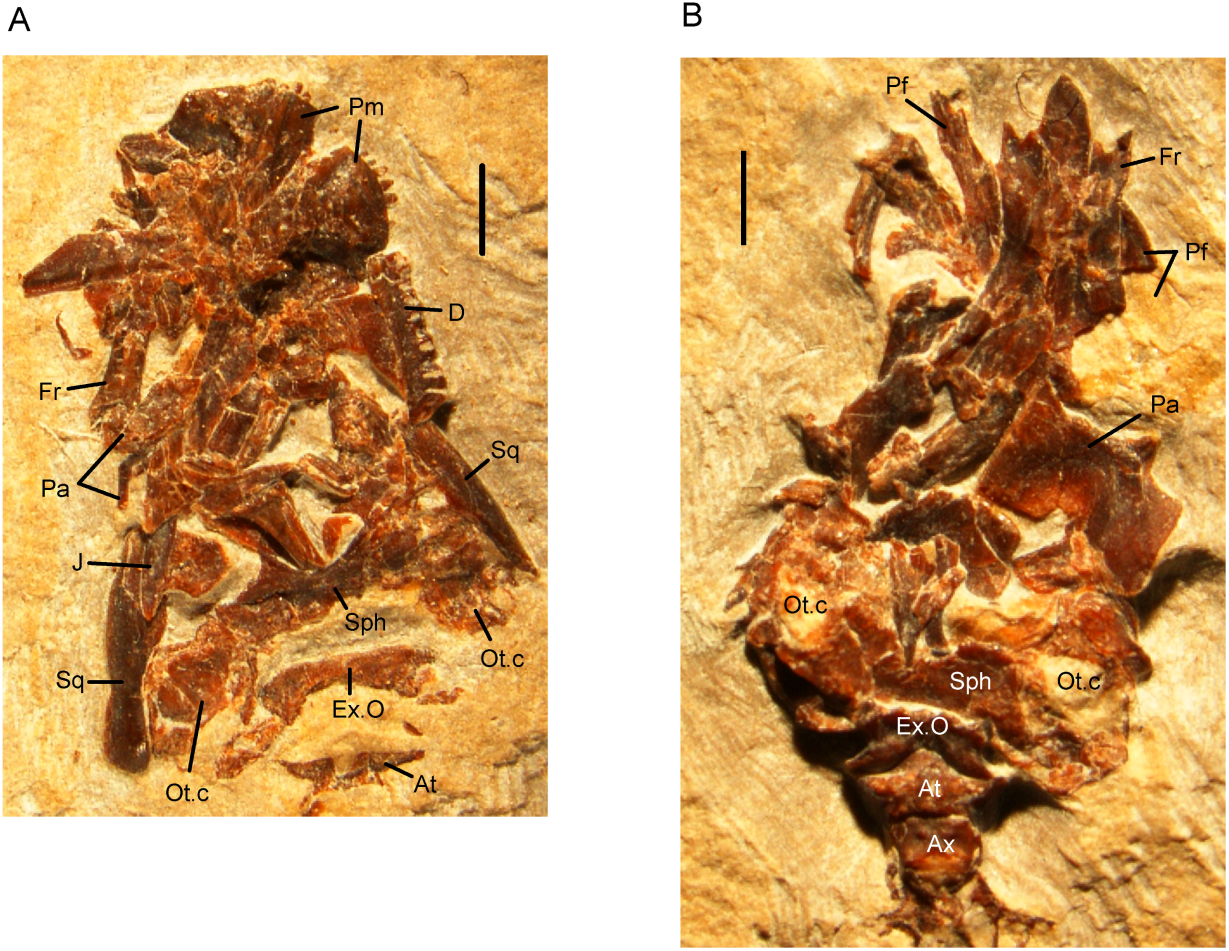
*Celtedens ibericus*, Lower Cretaceous (Barremian), Las Hoyas, Spain. digital photograph of specimen (Museo de las Ciencias de Castilla-La-Mancha, MCCM-LH-5710), skull of A, part and B, counterpart. Abbreviations: At, atlas; Ax, axis element; D, dentary; Ex.O, exoccipital; Fr, frontal; J, jugal; Ot.c, otic capsule; Pa, parietal; Pf, prefrontal; Pm, premaxilla; Sph, sphenoid; Sq, squamosal. Scale bars = 1 mm.

**Fig 19:**
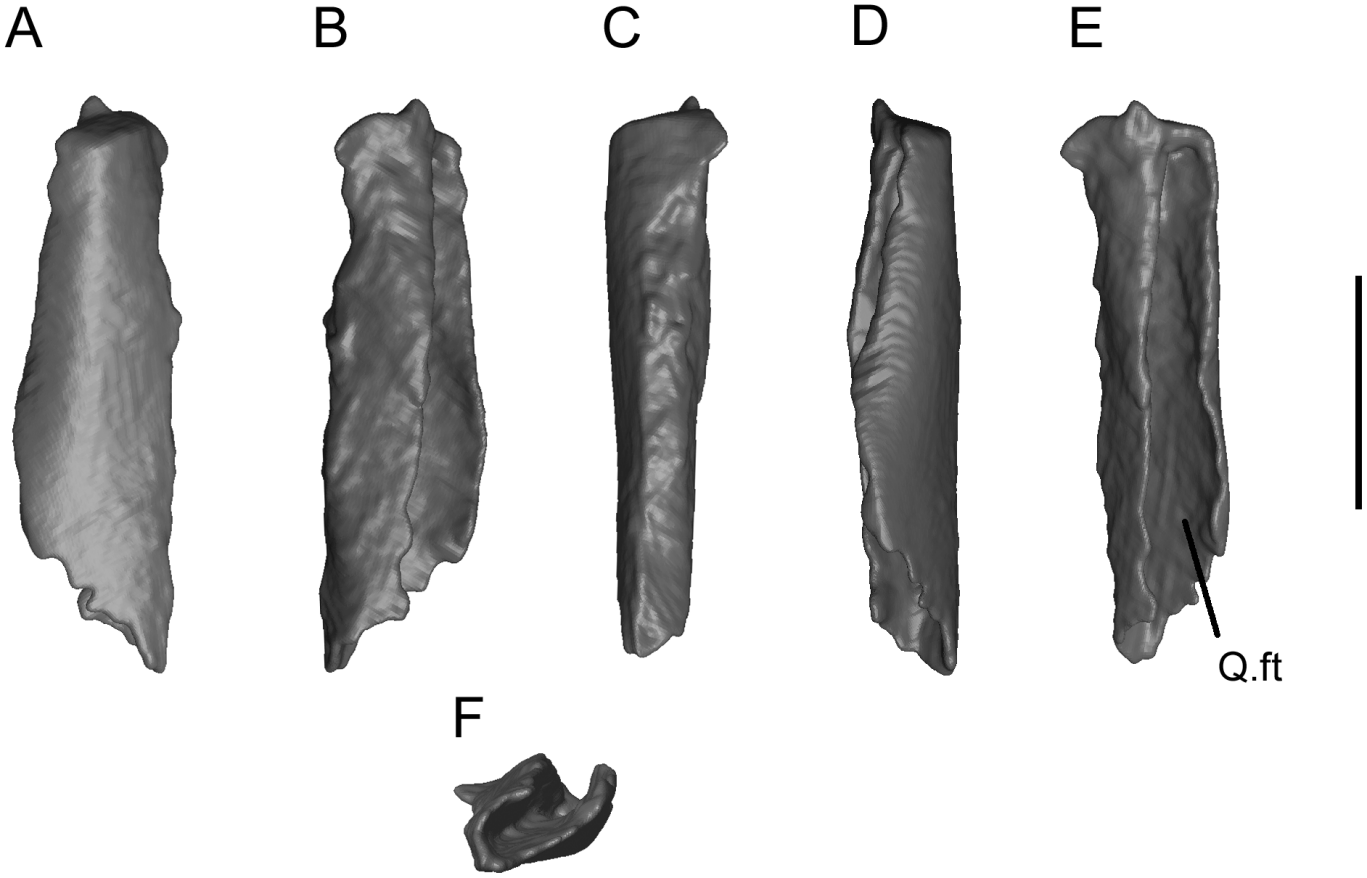
*Shirerpeton isajii* gen. et sp. nov., SBEI 2459, squamosal. Right squamosal as segmented from μCT slice data in A, anterodorsal; B, posteroventral; C, lateral; D, anteromedial; E, posteromedial; and F, ventral views. Abbreviations: Q.ft, quadrate facet. Scale bar = 1mm.

A rod-like element lying to the right of the frontal and right parietal (Figs 5–7) is interpreted as the right squamosal. It broadly resembles the squamosal preserved in the Las Hoyas *Celtedens ibericus* skull MCCM-LH15710 (Fig 18). As exposed on the Tetori block, the bone has the appearance of a slightly tapering blade but the μCT scans reveal a somewhat compressed cylinder, open along its medial side, with the posteroventral lamina slightly narrower than the anterodorsal one (Fig 19). The lateral margin is narrower than the open medial one and appears rugose but this edge is exposed on the block and the surface may be damaged. The cylinder is open at its anteroventral end, where the bone tapers to a lateral tip, and closed at its posterodorsal tip. However, this tip also lies at the edge of the block and the bone has broken at this point. We cannot therefore reconstruct the dorsal articular surface and are unable to determine precisely how the squamosal articulated with the rest of the skull. However, there is no evidence of an articulation surface for it on the parietal, and the squamosal almost certainly met the braincase in some way. Judging from the morphology of the quadrate, it slotted into the recess created within the squamosal, although the fit between the bones is imperfect. A partial left squamosal is associated with the left quadrate (Figs 5 and 6). Although the left squamosal reveals little detailed morphology, as the upper part of the bone has broken away, it has a slightly wider internal diameter than the right bone, suggesting that the latter has been somewhat compressed.

**Fig 20:**
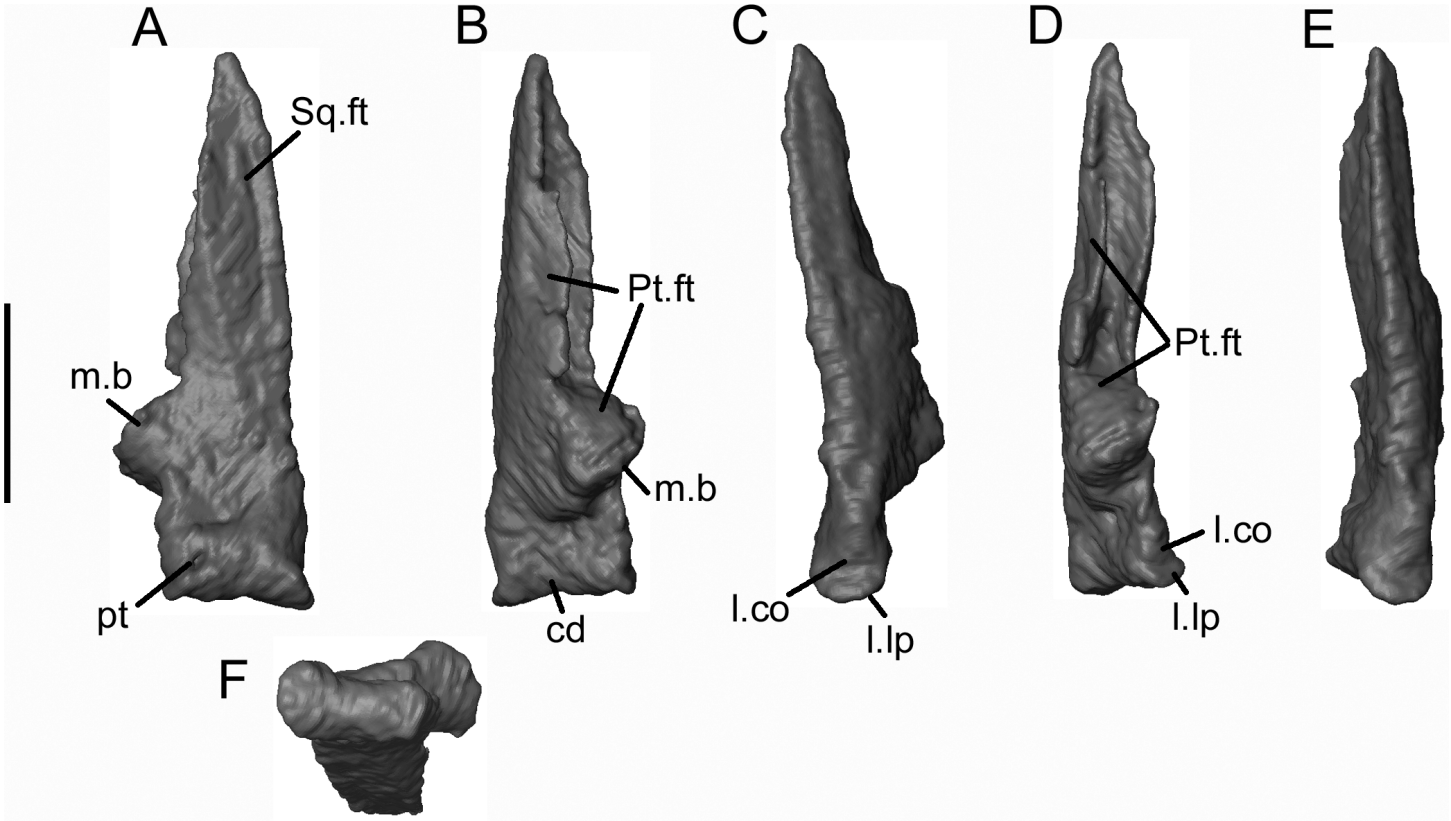
*Shirerpeton isajii* gen. et sp. nov., SBEI 2459, quadrate. Left quadrate as segmented from μCT slice data in A, anterodorsal; B, posteroventral; C, posterolateral; D, medial; E, lateral; and F, ventral views. Abbreviations: cd, condyle; l.co, lateral concavity; l.lp, lateral lip; m.b, medial boss; pt, pit; Pt.ft, pterygoid facet; Sq.ft, squamosal facet. Scale bar = 1 mm.

Both quadrates have been identified from μCT scans, lying to the right of the frontal and partially under the right parietal (Figs 5 and 6). The bone figured (Fig 20) is interpreted as a left bone by comparison with the holotype of *Celtedens ibericus* (MCCM-LH 6020) and extant salamanders. Estes and Hoffstetter figured a left quadrate of *Albanerpeton inexpectatum* in their skull reconstruction ([3] Fig 4) and as a very small component of their plate 5. Neither of these images shows much detail. The quadrate of *Shirerpeton* is a slender blade-like element with an anteroventral condyle for the articular and a tapering, facetted dorsal squamosal process that is hollowed out on its medial side. By comparison with other albanerpetontids, we interpret it as originally having been positioned with a strong posterodorsal to anteroventral angulation, so that the quadrate condyle met the transverse articular cotyle of the lower jaw almost horizontally. The quadrate condyle is asymmetric and has a pronounced lateral lip above which is a concavity (Fig 20C), the function of which is uncertain. It is not at an appropriate level to have met the tip of the jugal. A pronounced medial boss lies at roughly one- third of the height of the quadrate. The posteroventral surface of the bone is divided into three parts: a small ventral region above the condyle; a triangular area, apex medial, that incorporates the boss; and a slender, facetted and tapering dorsal process. This process is concave medially and convex laterally. The squamosal appears to have wrapped around the facetted lateral surface from anterior to posterior, whereas the pterygoid presumably met the quadrate shaft medially, attaching to the posterodorsal part of the medial boss and to a facet along the posteromedial edge of the quadrate blade. The anterodorsal surface (Fig 20A) is divided into a lateral pillar and a shallow medial concavity. A small rounded pit on the anteromedial corner of the bone (Fig 20A) extends onto the medial and then ventromedial aspect of the condyle leaving a small notch in its medial margin. This may have served for the attachment of a ligament stabilising the jaw joint.

The μCT scans revealed a number of other isolated elements, the identifications of which are uncertain given our limited knowledge of albanerpetontid cranial morphology. Nonetheless, three of these problematic elements are well preserved, morphologically distinctive, and appear to be cranial bones.

**Fig 21:**
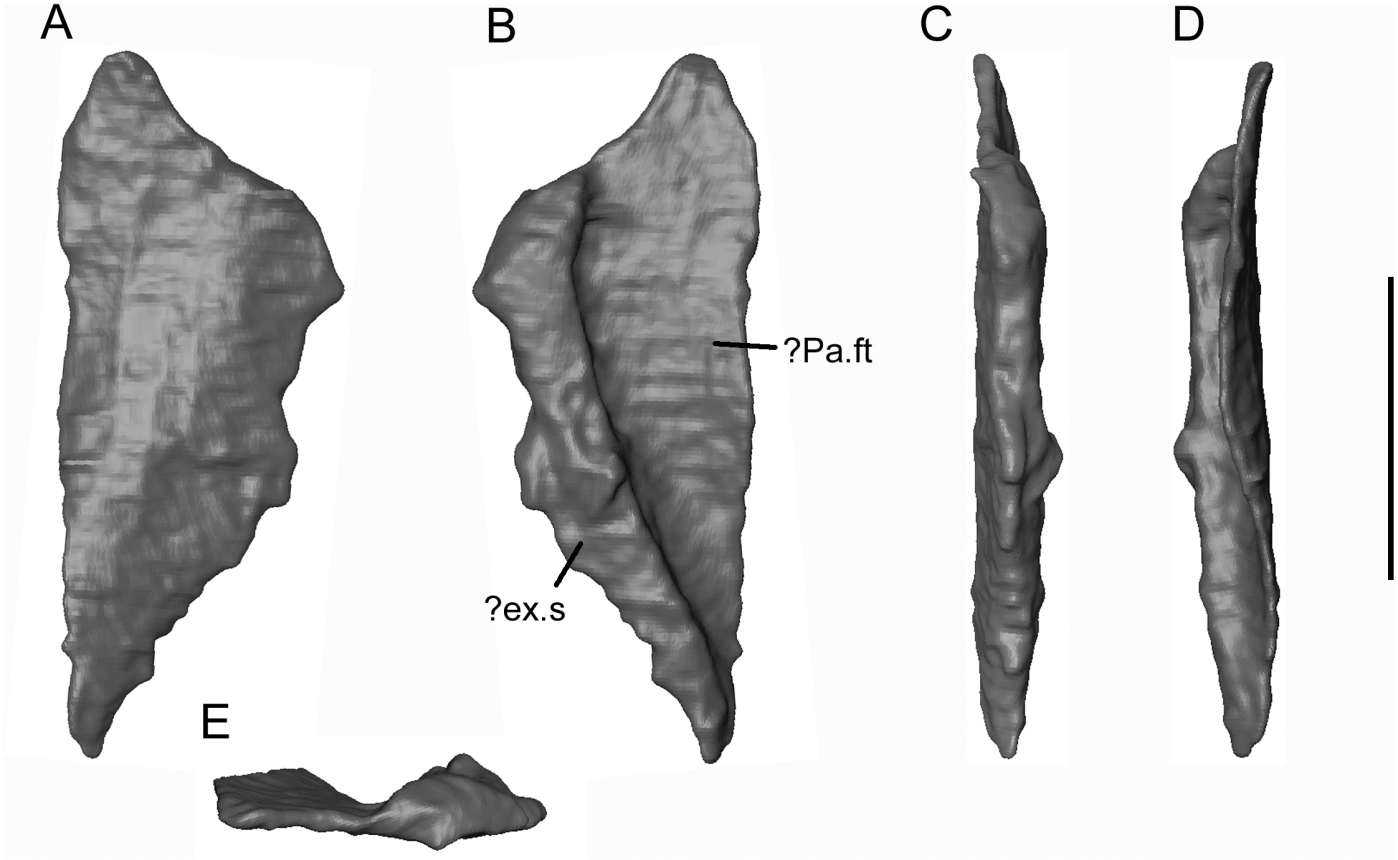
*Shirerpeton isajii* gen. et sp. nov., SBEI 2459, skull element. Possible left supratemporal element as segmented from μCT slice data in A, ventral; B, dorsal; C, lateral; D, medial; and E, anterior. Abbreviations:? ex.s,? exposed surface;? Pa.ft,? parietal facet. Scale bar = 1 mm.

The first (Fig 21), is a flat, half-rhomboid bone, with tapering ends. It lay adjacent to the lateral margin of the parietal (Figs 6 and 7). One long edge is thin and straight, the other is thickened and raised. One surface (possibly ventral) is featureless, the other (possibly dorsal) appears to be covered by a large deep facet. Correctly, or not, this element fits well into the lateral recess on the ventral surface of the parietal (Fig 3A), with the more acutely pointed end directed posteriorly and the raised edge exposed along the margin of the skull. As such, it could plausibly be a left supratemporal. Alternatively, as one edge is at the surface, it may represent part of a once larger element such as a pterygoid.

**Fig 22:**
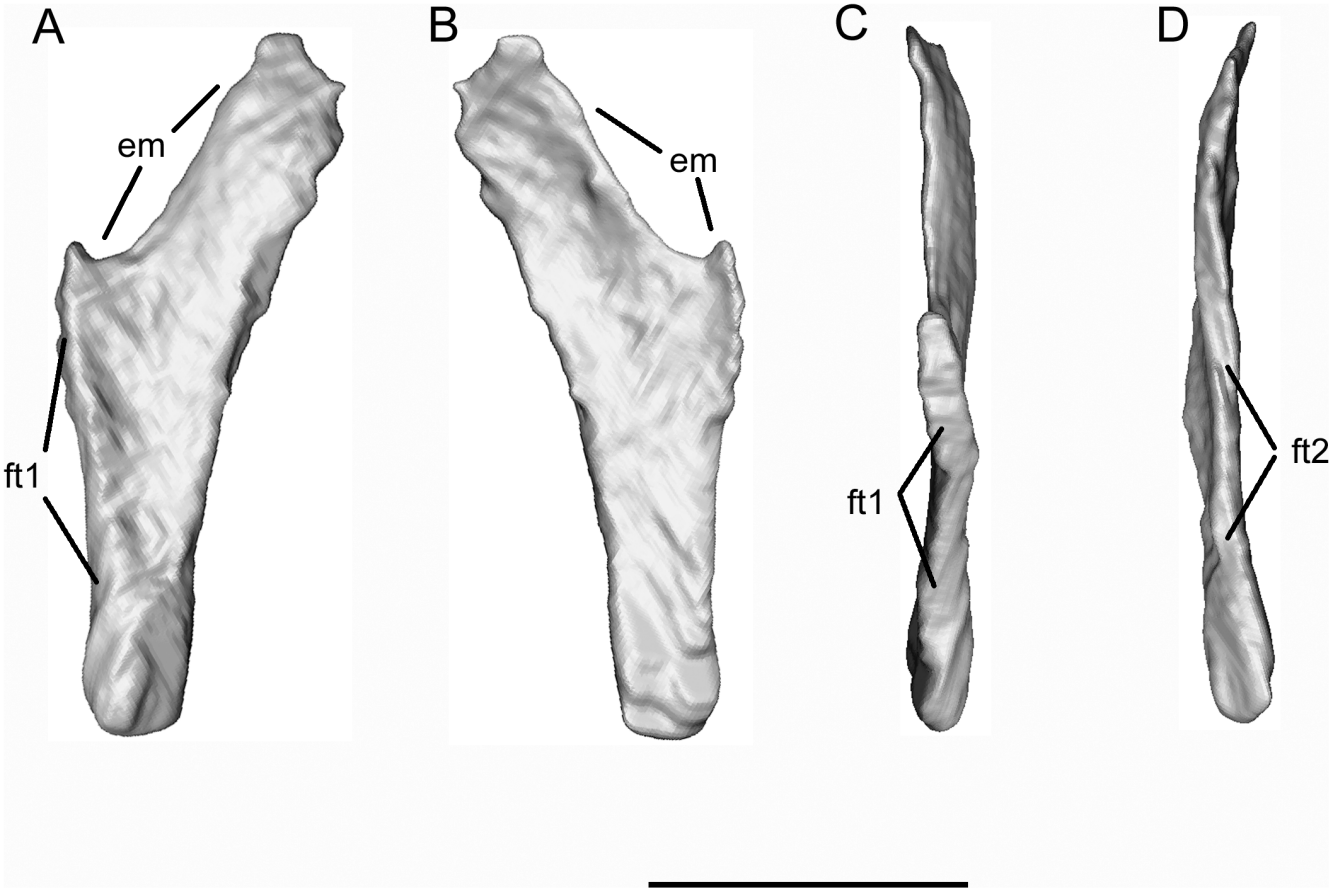
*Shirerpeton isajii* gen. et sp. nov., SBEI 2459, possible palatal element. Element as segmented from μCT slice data in A,? dorsal; B,? ventral; C,? lateral; and D,? medial views. Abbreviations: em, emargination; ft1, facet 1; ft2, facet 2 (as described in the text). Scale bar = 1 mm.

The second element (Fig 22) is a triangular bone with a long, slightly curved edge and two shorter edges, one thickened with an abutting facet (ft1) and the other thin and slightly embayed. There is some indication of a second facet (ft2) winding around part of this edge. On the block, the bone lies anterior to the tip of the frontal, close to the left maxilla (Figs 5–7). Aligned against the maxilla, the short thick edge could fit against the posterior part of the maxillary shelf. Depending on the orientation, the embayed edge (em) could correspond to the posterior limit of the choanal emargination, in which case it might be a palatine, or to the anterior edge of a subtemporal fenestra, whereby it might be an ectopterygoid.

**Fig 23:**
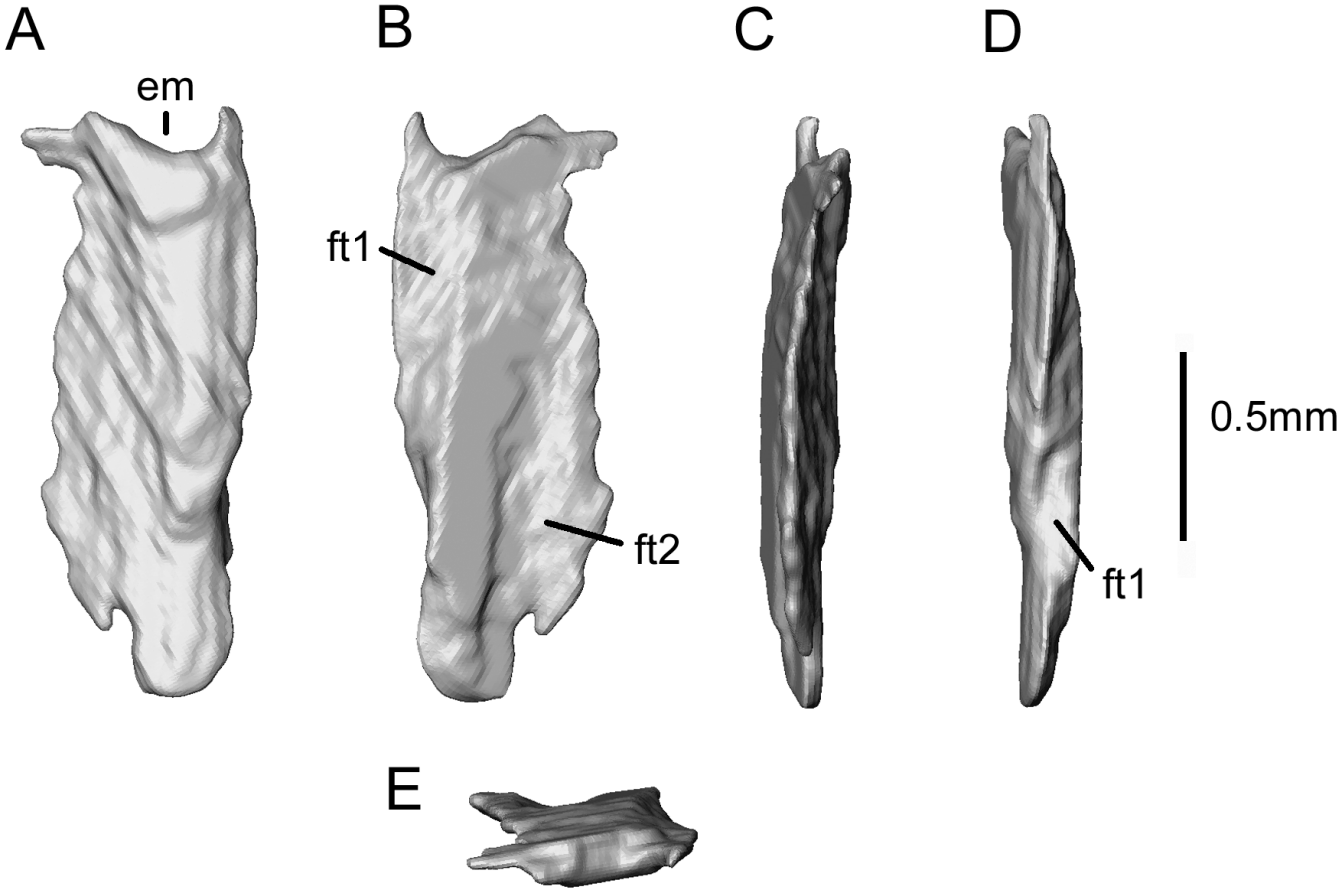
*Shirerpeton isajii* gen. et sp. nov., SBEI 2459, skull element. Unidentified skull element as segmented from μCT slice data in five views (anatomical orientation uncertain). Abbreviations: em, emargination; ft1, facet 1; ft2, facet 2 (see text). Scale bar = 0.5 mm.

The third bone (Fig 23) also seems to be complete and lay under the exoccipital, and close to the right maxilla and jugal (Fig 6,? Sk1). One surface, probably external, appears unfacetted (Fig 23A). The other, probably internal (Fig 23B), has facets on either side of an unfacetted flattened ridge. The narrower of the facets (Fig 23B, ft1) winds round the adjacent bone margin, suggesting that the margin abutted another element and then rested on a flange or ledge of that second element. The wider facet (Fig 23B, ft2) clearly overlapped a third bone. Of the shorter edges, one has a distinct embayment—suggesting it bordered an opening, whereas the other ends in a rounded lappet, with a distinct notch to one side of it—possibly partly enclosing a foramen. At present, we cannot identify this bone, although it must be part of the cranium. It is too small to be one of the main palatal elements and seems rather to have been exposed on the surface of the skull. If correct, this would again suggest the retention of a temporal element lost in crown lissamphibians.

#### Endocranium

**Fig 24:**
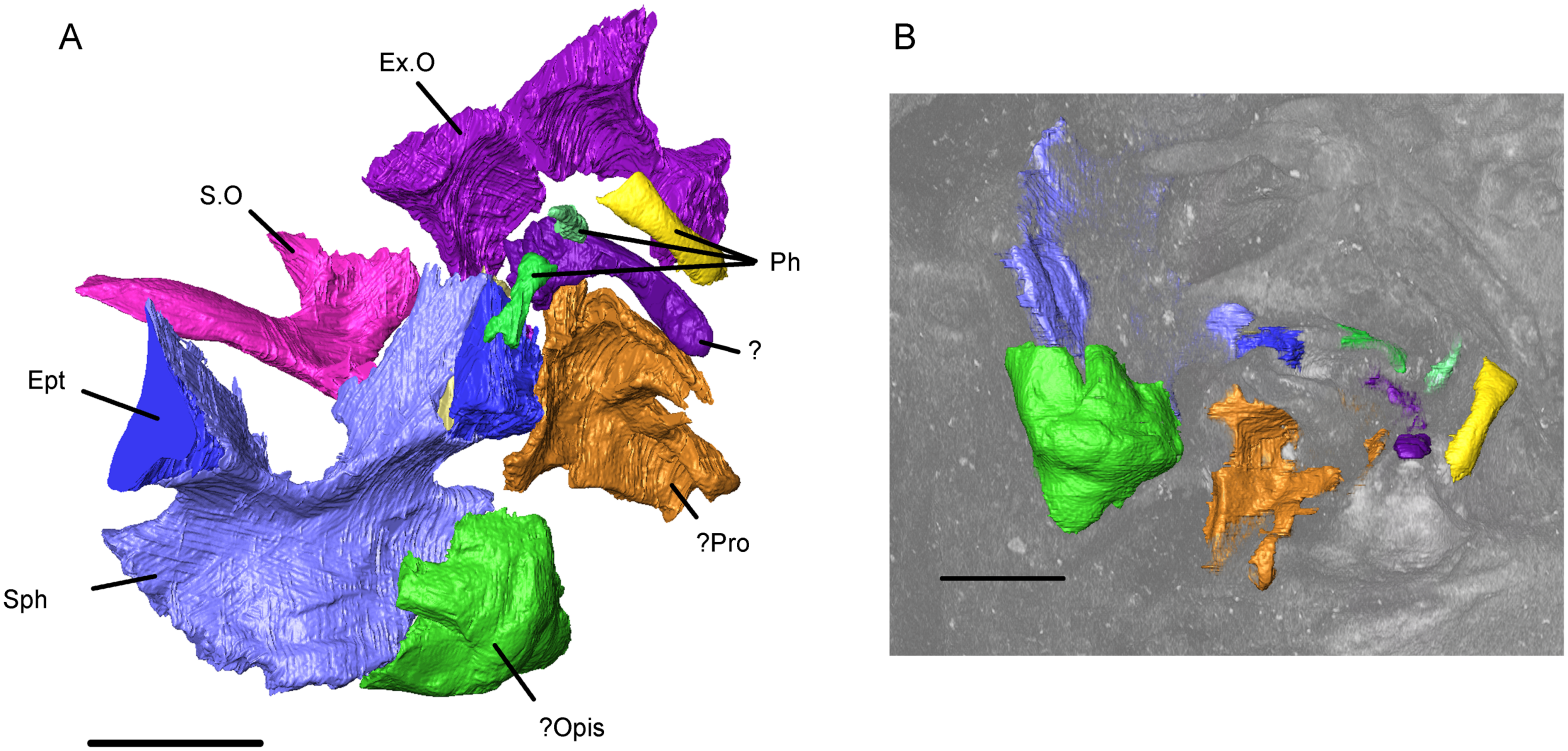
*Shirerpeton isajii* gen. et sp. nov., SBEI 2459, endocranium. A, Braincase elements as segmented from the μCT data; B, braincase elements as they appear at the surface of the matrix block, showing where erosion of the edges has occurred (note that the views in A and B are rotated in relation to one another, with B in the same orientation as in Fig 5). Abbreviations: Ept, epipterygoid; Ex.O, exoccipital plate;? Opis, possible opisthotic; Ph, parts of a digit;? Pro, possible prootic; S.O, supraoccipital; Sph, sphenoid;?, unidentified element. Scale bars = 1 mm.

The endocranium of albanerpetontids has been described for the Miocene *Albanerpeton inexpectatum* and the Pliocene *A*. *pannonicum* [3, 13]. In both species, it forms a single unit in which all components are fused and their original boundaries are uncertain. The endocranium of the Tetori albanerpetontid differs markedly in that the components are separate and are represented by the paired exoccipitals, a partial prootic and opisthotic, the sphenoid, and the supraoccipital (Fig 24). Associated with the sphenoid are paired epipterygoids.

**Fig 25:**
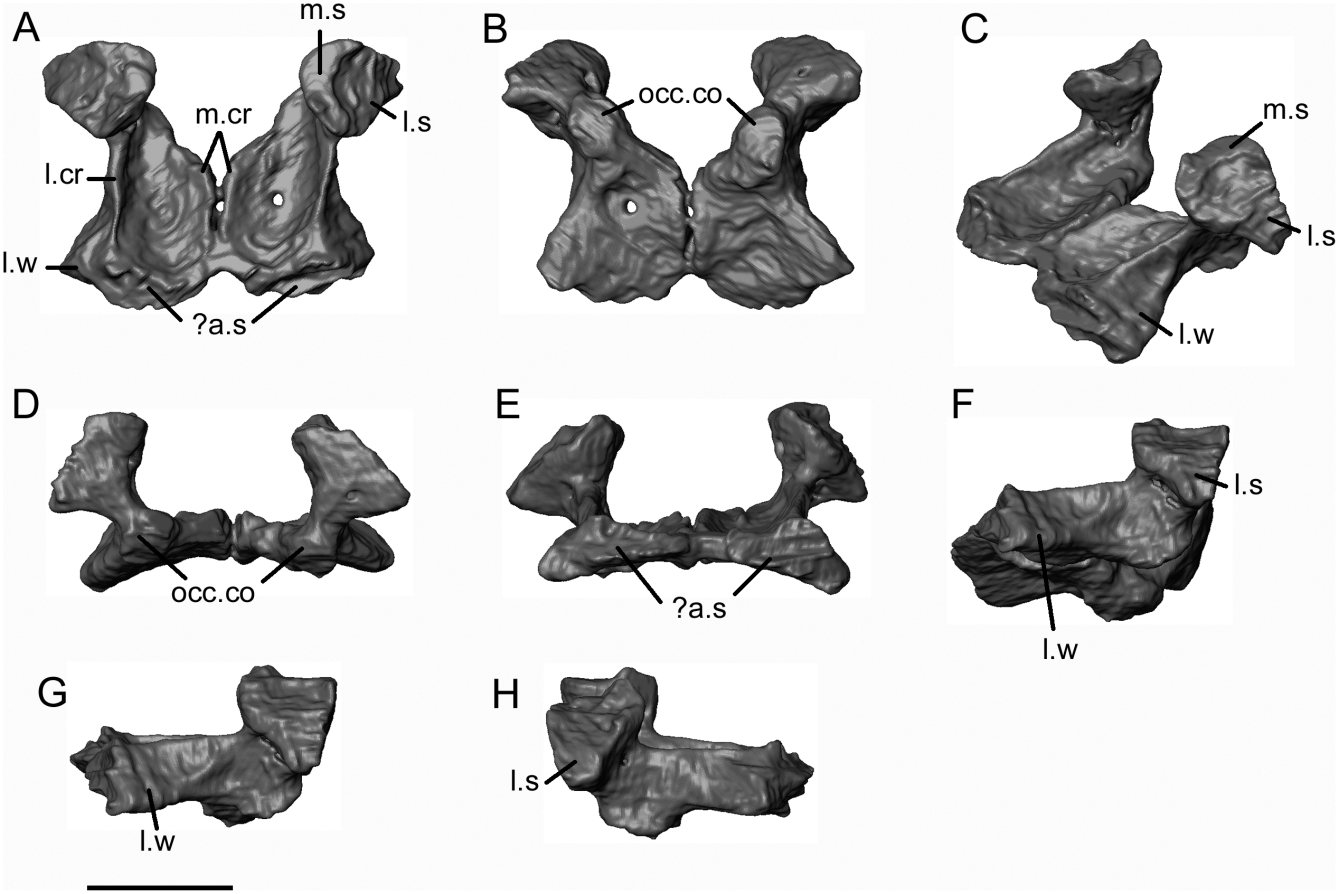
*Shirerpeton isajii* gen. et sp. nov., SBEI 2459, exoccipital plate. Conjoined exoccipitals as segmented from μCT slice data in A, dorsal; B, ventral; C, oblique left dorsolateral; D, posterior; E, anterior; F, oblique left ventrolateral; G, left lateral; and H, right lateral views. Abbreviations:? a.s, possible articular surface; l.cr, lateral crest; l.s, lateral surface; l.w, lateral wing; m.cr, medial crest; m.s, medial surface; occ.co, occipital condyles. Scale bar = 1 mm.

The exoccipitals (Fig 25) are partially fused along the anterior midline. Each comprises a short stout vertical pillar that expands both mediolaterally and anteroposteriorly into a large dorsal articular surface. The medial part of this surface accommodated the ventral process of the supraoccipital, but a more lateral surface remains and presumably met the otic capsule. The vertical pillar of the exoccipital bears some perforations but these are neither consistent in their position nor, when traced internally, do they show any clear path through the bone. We cannot therefore confirm whether these are openings for emissary veins or hypoglossal foramina, although the former is more likely. However, we can be certain that the exoccipital component is not perforated by a vagus/jugular foramen. If this foramen was present, it lay between the exoccipital and otic capsule. Ventrally, each exoccipital enlarges to form a thick, roughly rectangular basal plate that meets, and is partially fused with, the plate of the contralateral exoccipital. At the anteromedial junction of the vertical and horizontal parts of each exoccipital, there is a weakly-developed oval occipital condyle (possibly partly cartilaginous) that faces ventrally and medially (Fig 25B and 25D). The lateral margins of the basal plate are divergent so that the anterior edge is wider than the posterior one. Moreover, the plates are separated posteromedially, leaving the posterior margin of the bone V-shaped. The ventral surface of the basal plate is flat (Fig 25B). Dorsally, each plate bears a concavity between medial and lateral crests (Fig 25A). The medial crest borders the medial edge of the bone and, together, the contralateral medial crests form a double midline ridge that may originally have enclosed the anterior part of the notochord. The lateral crest extends from the base of the vertical exoccipital component to the anterior margin. It runs roughly parallel to the medial crest rather than diverging in line with the lateral margin. As a result, the lateral crest is flanked at its anterior end by a deep lateral wing of bone that bears an external concavity. However, the intact dorsal margin of the lateral crest appears to lack any articular surface and may therefore have formed the ventral margin of a natural opening (?fenestra vestibuli). Seen in anterior view (Fig 25E), the conjoined borders of the basal plate lie horizontally, although there is a ventrolateral expansion on either side of the midline where the bone expands into the lateral wing. This anterior edge appears to be complete and bears a vertical surface, although it is not clear whether this is an articular surface.

**Fig 26:**
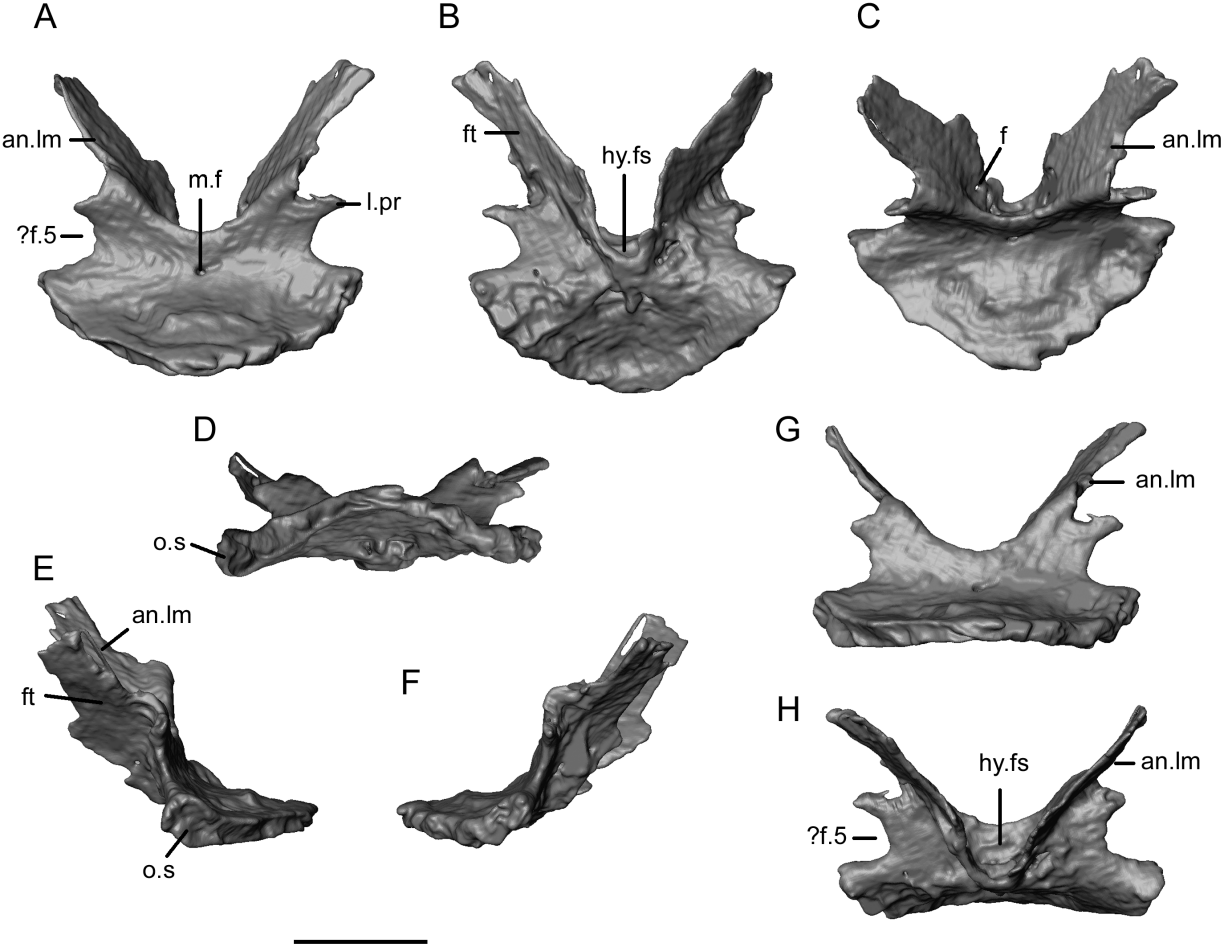
*Shirerpeton isajii* gen. et sp. nov., SBEI 2459, sphenoid. Sphenoid as segmented from μCT slice data in A, posterodorsal; B, anteroventral; C, dorsal; D, posteroventral; E, left lateral; F, right lateral; G, posterior; and H, anterior views. Abbreviations: an.lm, antotic lamina; f, small foramina; ft, facet;? f.5, possible trigeminal foramen; hy.fs, hypophyseal fossa; l.pr, lateral process; m.f, median foramen; o.s, oval surface. Scale bar = 1 mm.

The second endocranial element is a large complex symmetrical bone that we interpret as a compound sphenoid (Fig 26). The horizontal posterior plate has a thickened rounded posterior margin, again with a shallow vertical surface. However, the posterior midline and right side edges of the plate were exposed on the surface of the block, and may not be intact. The vertical surface runs around the rim of the horizontal plate, but is enlarged into a deeper ovoid surface at the anterolateral corners. These anterolateral surfaces may represent the basal articulations for the pterygoids. The anterior part of the bone turns dorsally at an angle of around 70 degrees to the basal plate. Seen in posterior view (Fig 26G), this vertical part of the bone is biradiate with tall anterodorsal antotic laminae forming a deep U-shaped dorsal margin, and double-notched lateral margins separated by a small lateral process (Fig 26A and 26B). We interpret the larger lower notch on each side as contributing to the margin of a foramen for the fifth cranial nerve (trigeminal). The upper notch is associated with a facet on the anterolateral surface of each blade. Seen in anterior view (Fig 26H), the bifurcating antotic laminae, presumably ossifications into the pila antoticae [70], flank a central recess that we interpret as the hypophyseal fossa. However, this has no floor and there is no trace of a parasphenoid rostrum. It therefore seems likely that the dermal parasphenoid was not fused to the endochondral basisphenoid or basal plate in this specimen, but may originally have underlain both. At their bases, the antotic laminae are perforated by small foramina (Fig 26C), possibly for branches of the internal carotid artery leaving or entering the median fossa. A single median foramen perforates the anterodorsal surface (Fig 26A).

**Fig 27:**
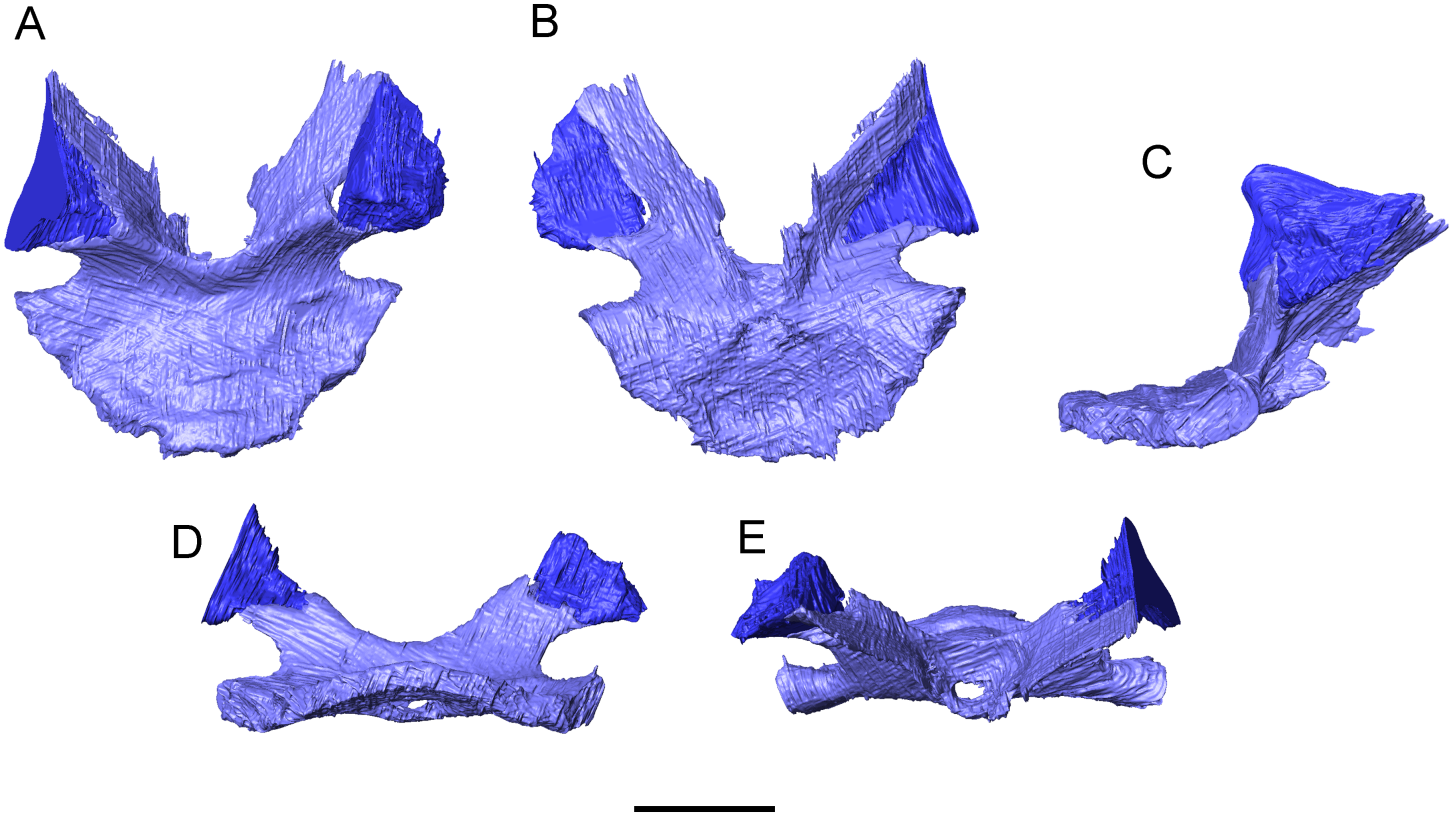
*Shirerpeton isajii* gen. et sp. nov., SBEI 2459, sphenoid and epipterygoids. Sphenoid (blue/purple) in association with epipterygoids (darker blue) as segmented from μCT slice data in A, dorsal; B, ventral; C, right lateral; D, posteroventral; and E, anteroventral views. Note that the apparent opening in the hypophyseal region is an artefact of segmentation, this image having been rendered from the slice data of the higher resolution scan. Scale bar = 1 mm.

In the scan slices, each of anterolateral facets on the antotic laminae is seen to support an additional element that is blade-like ventrally but expands and thickens dorsolaterally into a broad rugose surface (Fig 27). When the endocranial elements are positioned in the 3-D physical model, the apices of these dorsal elements align with anterolateral concavities in the ventral surface of the parietal (as described above). We therefore interpret the dorsal elements as epipterygoids.

**Fig 28:**
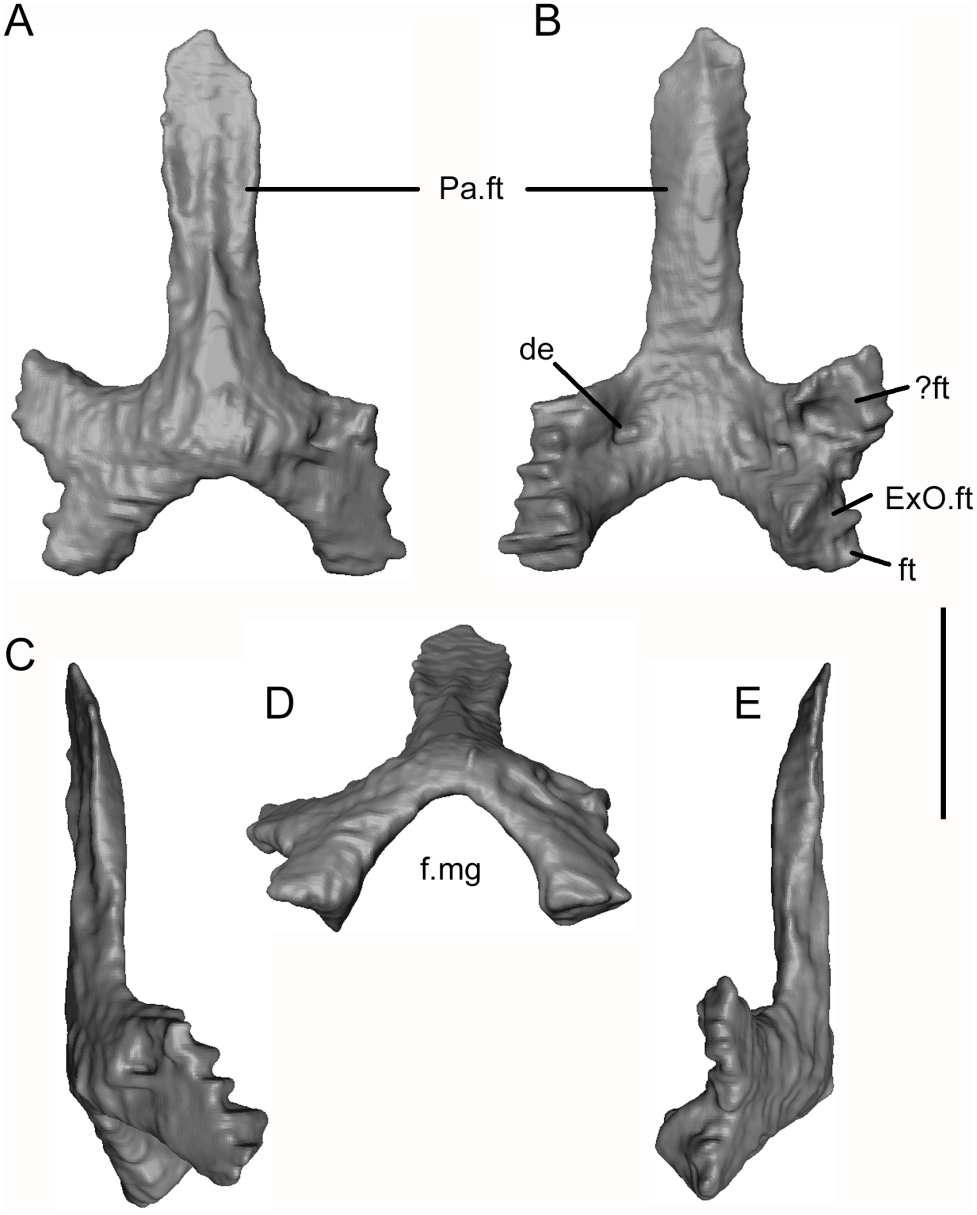
*Shirerpeton isajii* gen. et sp. nov., SBEI 2459, supraoccipital. Supraoccipital as segmented from μCT slice data in A, dorsal; B, ventral; C, right lateral; D, posterior; and E, left lateral views. Abbreviations: de, ventral depression; ExO.ft, exoccipital facet; f.mg, foramen magnum; ft, separate part of exoccipital facet, possibly for part of otic capsule;? ft, possible otic capsule facet; Pa.ft, parietal facet. Scale bar = 1 mm.

The third endochondral element preserved is a pentaradiate bone (Fig 28) lying adjacent to the exoccipitals and right parietal (Fig 24A). It is complete except for the right anterolateral corner, and its symmetrical shape identifies it as a median element. Given its long anterior process, a parasphenoid would be a plausible identification. However, the vaulted shape of the posterior end and the pattern of facets precludes this interpretation as it would not be possible to fit this element to the sphenoid described above. Moreover its posteroventrolateral facets fit the dorsomedial surfaces on the exoccipitals. The bone is therefore an ossification in the dorsal roof of the endocranium, by definition a supraoccipital. As seen in dorsal view (Fig 28A), the posterior arch is narrow and vaulted, with a deep posterior embayment forming the dorsal margin of the foramen magnum. To either side, there are short bifurcated lateral wings and there is a large anterior median process. This median process bears paired dorsal and ventral facets (Fig 28A–28C and 28E) and, in life, would have slotted between the posteromedial processes of the parietals. At the junction of the anterior process and the main body of the bone, the paired facets are separated by a short crest that would have intervened between the posterior tips of the parietals. The ventral surface of the arch is penetrated on each side by a small but conspicuous depression that may be a foramen, but the resolution makes this too difficult to determine. The right ventrolateral margin is damaged but the left is divided into anterior and posterior parts. The anterior part is medially concave but whether it is a facet for the otic capsule or formed part of the intracranial roof remains uncertain. The posteromedial edge clearly articulated with the medial edge of the exoccipital, but the posterolateral edge lies at a different angle and combines with the lateral exoccipital surface to form a large articular surface that was presumably for the otic capsule. Unfortunately, the incomplete preservation of the prootic and opisthotic (see below) renders a full reconstruction impossible.

An ossified tectum synoticum was reported as present in the *Albanerpeton inexpectatum* and *A*. *pannonicum*, but in the absence of visible sutures, Maddin et al. [13] were unable to determine whether this tectal ossification represented a separate supraoccipital ossification or a non-homologous membrane bone extension from the edge of the exoccipital. In the much older, and presumably more primitive Japanese taxon, it is clear that the endocranium is roofed by an independent ossification. The articulation of this element with the exoccipitals suggests that it incorporated at least a tectum posterius, but whether there was also a tectum synoticum is unclear.

The otic capsules of *Shirerpeton* are represented by incomplete elements, tentatively interpreted as parts of the left opisthotic and right prootic. They are separate from one another but lie close to both the sphenoid and exoccipital plates (Fig 24). Unfortunately they are also at the surface of the block and both have been damaged.

**Fig 29:**
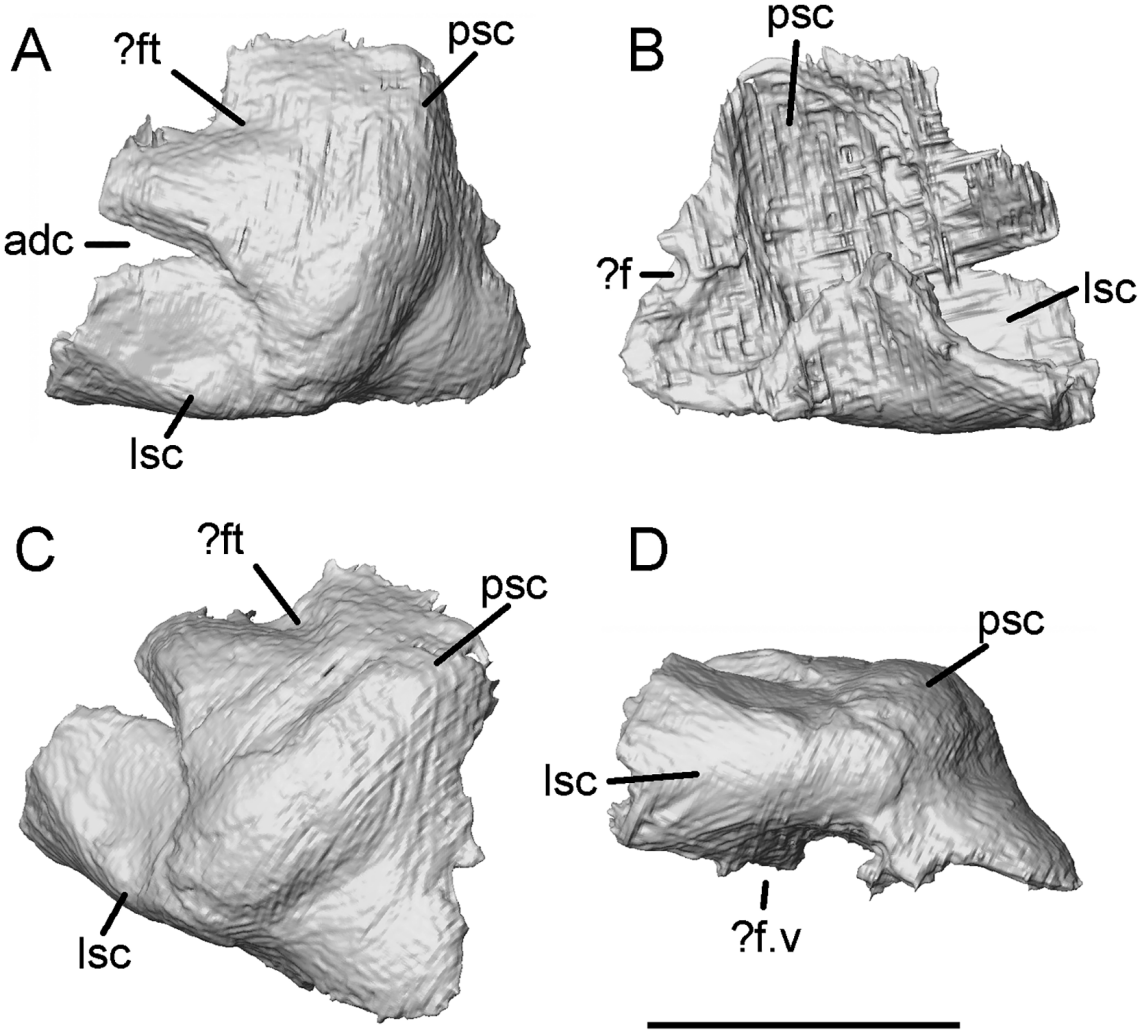
*Shirerpeton isajii* gen. et sp. nov., SBEI 2459, otic capsule. Possible left opisthotic as segmented from μCT slice data in A, dorsolateral; B, medial; C, posterolateral; and D, ventrolateral views. Abbreviations: adc, anterodorsal cleft;? f, curved edge of possible foramen;? f.v, possible position of fenestra vestibuli;? ft, surface depression that may represent a facet for a roofing element; lsc, groove or prominence for lateral semicircular canal; psc, groove or prominence for posterior semicircular canal. The apparent anterodorsal cleft is due to damage. Scale bar = 1 mm.

The opisthotic is represented by an irregular rectangle of bone that lay on the surface of the block, with its anterior margin crushed into the sphenoid (Fig 29). Its external surface is framed posteriorly and laterally by rounded ridges that we interpret as marking the courses of the posterior and lateral semicircular canals respectively. They are matched internally by deep grooves. Viewed in lateral aspect, the anteroventral margin of the bone is curved and probably formed part of the posterior margin of the fenestra vestibuli. Depressions on the dorsal and dorsolateral margins may represent contact points for roofing or suspensory elements, but the resolution is not good enough to be certain.

**Fig 30:**
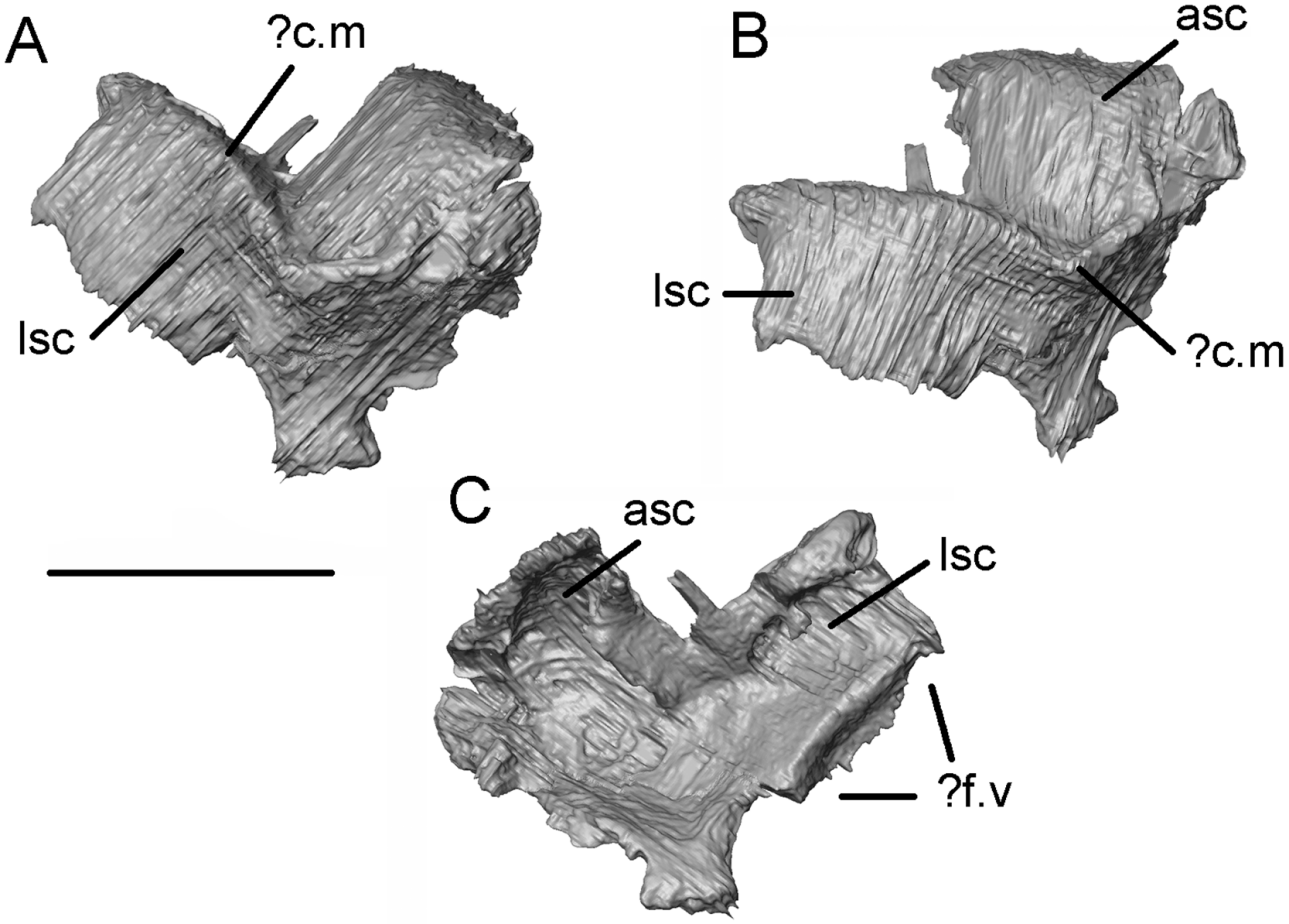
*Shirerpeton isajii* gen. et sp. nov., SBEI 2459, otic capsule. Possible right prootic as segmented from μCT slice data in A, anterolateral; B, lateral; and C, posteromedial views. Abbreviations: asc, groove or prominence for the anterior semicircular canal;? c.m, possible crista muscularis;? f.v, possible edge of fenestra vestibuli; lsc, groove or prominence for lateral semicircular canal. Scale bar = 1 mm.

The prootic, as interpreted, is an irregular element in which dorsal, anteroventral, and lateral laminae meet at a sharp external corner (Fig 30). A strong right-angled crest separates the dorsal lamina from both the lateral and anteroventral ones. By comparison with Neogene albanerpetontids [13], we interpret this crest, at least in part, as the crista muscularis. Seen in medial view, the dorsal and lateral laminae are grooved by what we interpret to be the anterior and lateral semiciricular canals. Each has a deeper ventral depression that may have held the respective ampullae. The anterodorsal portion of the bone is deeply grooved and probably held the anterior semicircular canal and its ampulla.

Posterior to the left parietal and to the left of the basisphenoid and opisthotic, though roughly on a line with these, there is a thick (?endochondral) element with a rounded external surface, one thick rugose end, and internal concavities (dark grey element labelled? in Figs 5–7). Due to its surface exposure at the edge of the block, its outline cannot be reconstructed with assurance but it is possible that this is also part of one of the otic elements.

**Fig 31:**
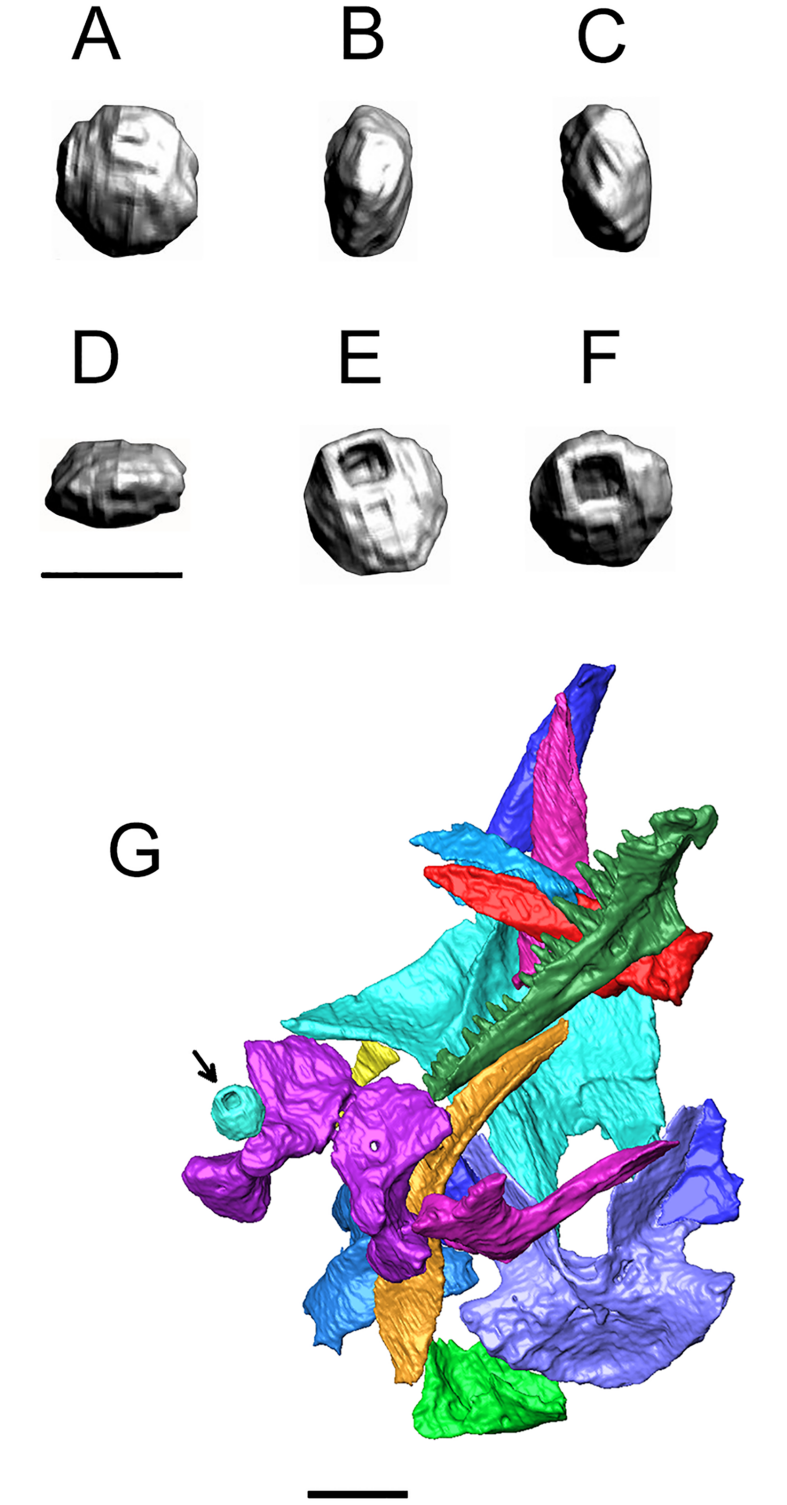
*Shirerpeton isajii* gen. et sp. nov., SBEI 2459, ossicle. A-F, Otic associated element as segmented from μCT slice data in six views (anatomical orientation uncertain). G is a partial view of the segmented braincase showing the position of this small element (arrowed) in relation to the exoccipital plate (but note that this view is rotated relative to the entire block in Fig 6). Scale bars A-F = 0.5 mm; G = 1 mm.

One further element is of potential interest (Fig 31). It is a small, hollow, subspherical element that was positioned adjacent to the exoccipitals (Fig 31G). One surface is circular, the obverse surface contains a large, almost square foramen that is asymmetrically placed and opens into the hollow interior. The hollow centre suggests part of the bone may have remained cartilaginous. The sides are rounded, and bear some distinct rugosities, but overall the element is compressed (either mediolaterally or dorsoventrally depending on its original position). A survey of extant amphibian cranial morphology has failed to provide a clear match, but given the rounded shape an operculum would be a possibility.

#### Mandible

**Fig 32:**
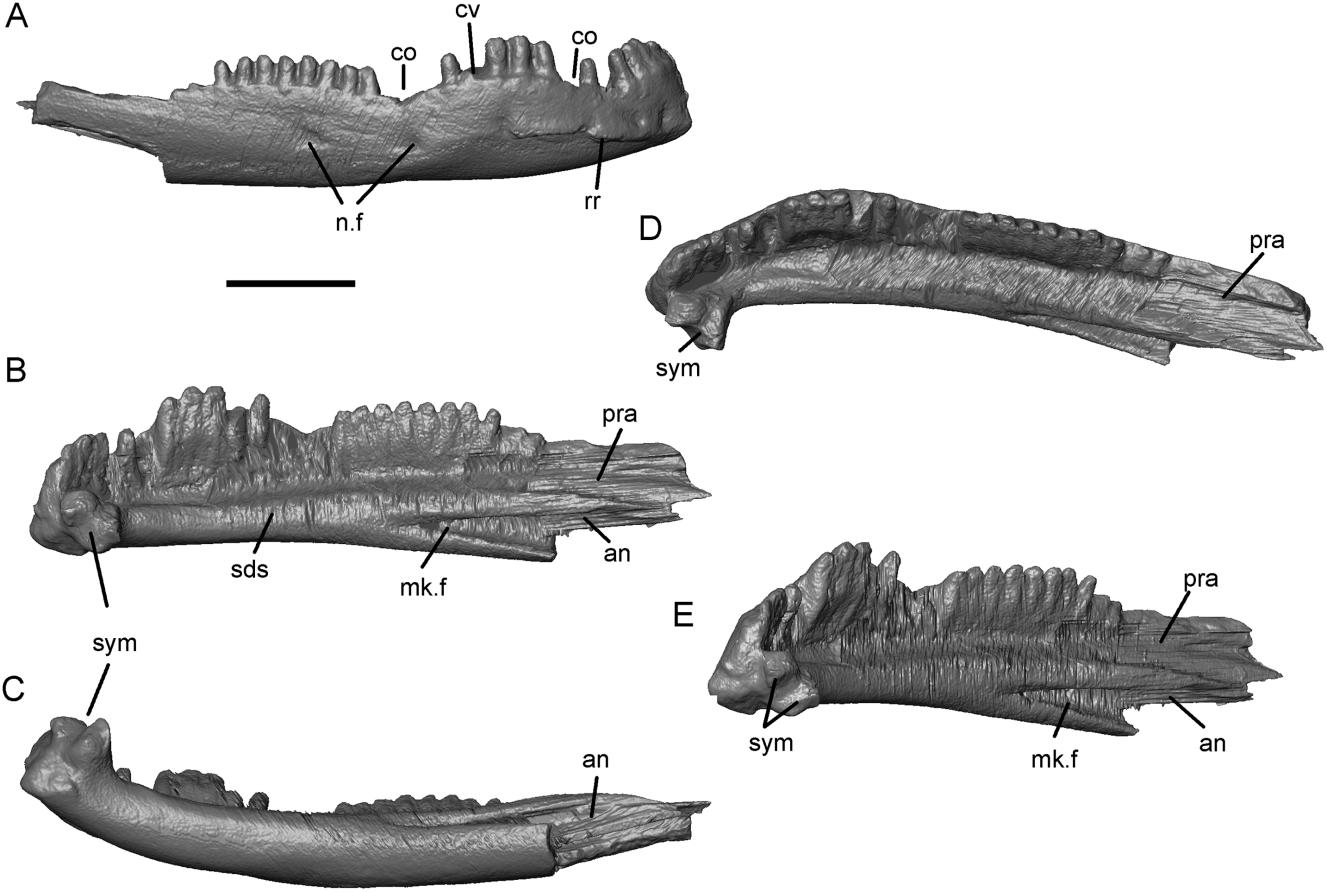
*Shirerpeton isajii* gen. et sp. nov., SBEI 2462, a right dentary, as segmented from μCT slice data. A, labial view; B, lingual view; C, ventral view; D, occlusal view; E, anterolingual view. Abbreviations: an, angular facet; co, concavity in dorsal jaw margin; cv, convexity in dorsal jaw margin; mk. f, Meckelian fossa; n.f, nutrient foramina; pra, prearticular facet; rr, rugose ridge; sds, subdental shelf; sym, symphyseal region with prongs; Scale bar = 1 mm.

There is no lower jaw material associated with SBEI 2459. However, SBEI 2459 is supplemented by SBEI 2405 and SBEI 2462 (Fig 4), two albanerpetontid right dentaries recovered separately but referred to *Shirerpeton* on the basis of their small size and the rarity of albanerpetontid bones in the deposit (3 out of 2459 catalogued specimens). SBEI 2405 is almost complete and is 4.5 mm long and less than 1.0 mm high at its deepest point. It may represent a smaller individual than the holotype specimen. SBEI 2405 was originally preserved in labial view (Fig 4A) but was extracted manually from the matrix resulting in some loss of surface detail. SBEI 2462 is broken at the posterior end and is from a slightly larger individual than SBEI 2405. It was scanned in the matrix, and at higher resolution than SBEI 2405, yielding more detailed images (Fig 32). Overall, the dentary of *Shirerpeton* is relatively long and shallow but the jaw depth varies along the jaw (Fig 32A). It is shallowest just posterior to the symphysis due to a concavity (co) in the anterodorsal margin. This margin then expands into a convexity (cv) that spans seven or eight tooth positions. As in the maxilla, the greatest expansion coincides with the largest teeth. Posterior to the convexity, there is a second concavity. This pattern of concavities in the dorsal jaw margin on either side of a convexity is seen on both dentary specimens and is therefore unlikely to be an artefact of breakage. Posterior to the second concavity, the alveolar margin straightens, angling dorsally so that the rear of the dentary is its deepest part. However, on the lingual surface, the dorsal margin of the subdental shelf rises more gradually. As a result, the posterior teeth are similar in size to those in the symphyseal region (rather than markedly smaller as in some albanerpetontid species). The labial surface is perforated by at least six nutrient foramina (SBEI 2462, Figs 4E and 32A). In its anterior part, this surface is also clearly divided into lateral and ventrolateral regions, partly demarcated in SBEI 2462 by a rugose crest (Fig 32A). There are 23–28 tooth positions and, as in the maxilla, there is heterodonty in tooth size, with the teeth adjacent to the labial convexity both taller and broader than either the anterior or posterior teeth. The lingual surface (Figs 4C, 4D, 32B and 32E) confirms details of albanerpetontid morphology such as the tooth implantation, lack of pedicelly, the presence of symphyseal prongs, and a Meckelian fossa that is closed for about two-thirds of its length (to approximately the 18-19th tooth position). The resolution of the scans does not permit the morphology of the tooth crowns to be described. Facets within the opening into the Meckelian fossa may have accommodated the angular (Figs 4D and 32B–32E), whereas a second facet posterodorsal to the tooth row probably served for the prearticular (Fig 32B–32E). This facet appears to lie mainly or entirely posterior to the level of the tooth row, unlike many albanerpetontid taxa where it extends forward below the tooth row roughly to the level of the opening into the Meckelian fossa.

### Postcranial skeleton

None of the postcranial skeleton is visible on the surface of the SBEI 2459 block but the μCT scans have revealed the presence of eight vertebrae or partial vertebrae, some ribs, a small long bone lying mainly to the left of the skull bone mass, and elements of what appear to be a single digit.

**Fig 33:**
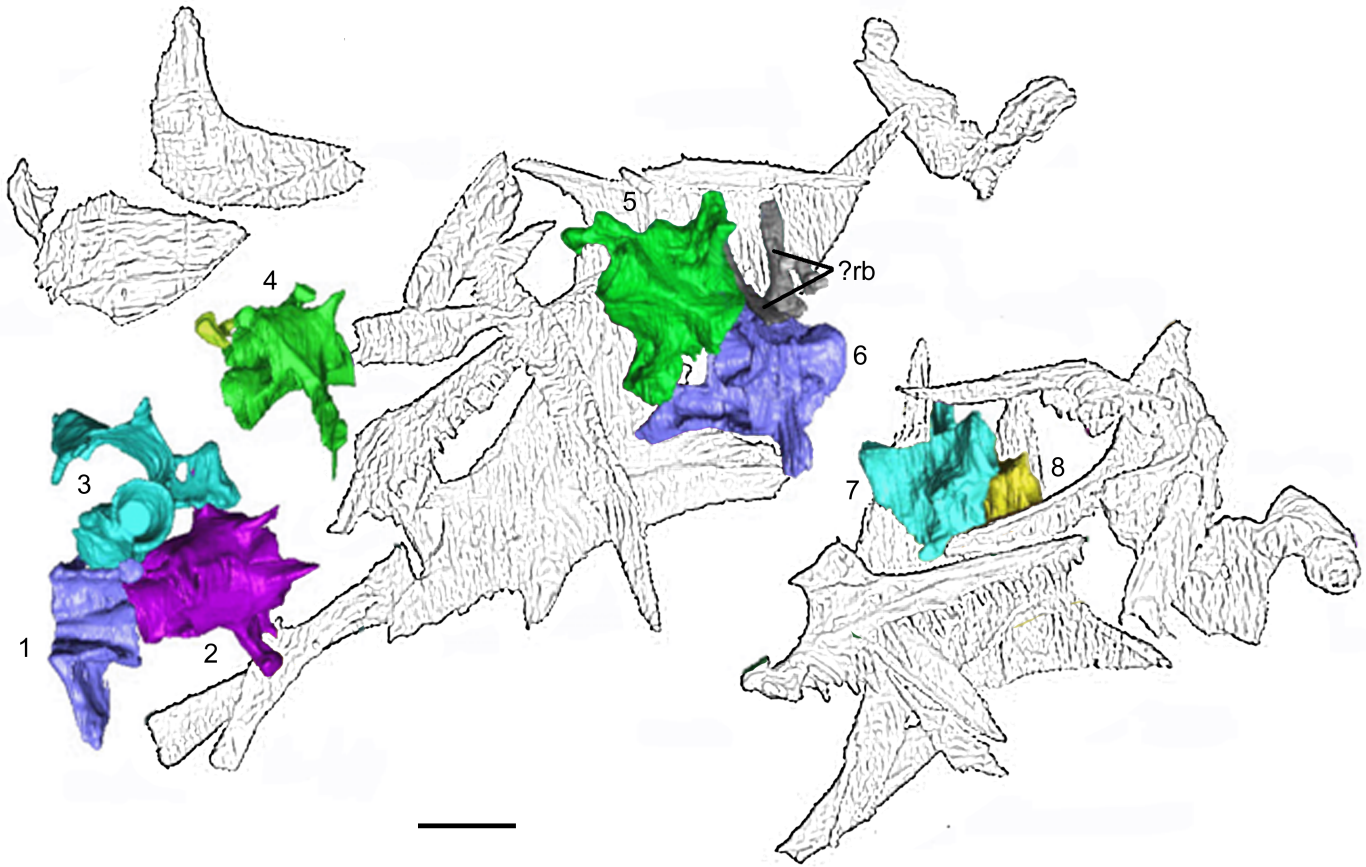
*Shirerpeton isajii* gen. et sp. nov., SBEI 2459, vertebrae. Associated vertebrae as segmented from μCT slice data. The numbers relate to the text description. Abbreviations:? rb, rib-like elements illustrated in Fig 34. Scale bar = 1 mm. Note that this figure is rotated relative to the scans of the entire specimen in Figs 5 and 6.

Collectively, the vertebrae form a curved series below the skull elements (Fig 33), and each has the anterior zygapophyses directed away from the anterior edge of the block. However, they are disarticulated and it is not clear whether they once formed a morphological sequence. For descriptive purposes, the vertebrae are numbered as 1 through 8. Three vertebrae (1–3) lie within the matrix close to the nasal at the anterior end of the block, another (4) lies behind them close to the left maxilla, two (5–6) lie below the posterior end of the frontal, and two (7–8) lie below the right parietal. Of the latter, one (7) has its transverse process exposed between the posterior parietal rami. The anterior cluster is represented by two relatively complete vertebra, and one (1) that has been truncated at the edge of the block. This further demonstrates that part of the specimen was lost during an initial stage of block trimming.

Albanerpetontid vertebrae have been described briefly elsewhere [3, 6, 8, 23]. Apart from the unique atlas-axis complex, the vertebrae are relatively unremarkable. The only known complete albanerpetontid skeletons (*Celtedens ibericus*, [6, 8]) have an estimated 20 post-axial presacral vertebrae, one sacral, and about 24 caudals. Albanerpetontid vertebrae are fully notochordal with short unicipital transverse processes bearing free ribs in the presacral series and longer transverse processes and hypapophyses in the tail. The vertebrae of *Shirerpeton* conform to this morphology. As in other albanerpetontids, the centrum is relatively long, and notochordal, with a median constriction. Ventrally, all but one of the centra bear a keel but there are no traces of hypapophyses. The exception (vertebra 4) has a more rounded keel but also long tapering transverse processes that suggest it is an anterior caudal. Vertebra 1 also has tapering transverse processes, although these are shorter. The remaining vertebrae have short, deep transverse processes and are therefore probably from the trunk region, although the depth of the processes in vertebra 5 suggests it could be a sacral. The most complete neural arches are those of vertebrae 2, 5, and 6. They bear small, elongate, anterior and posterior zygapophyses, with curved articular surfaces, set at an angle of roughly 45 degrees to the horizontal. The neural spine is positioned posteriorly and is supported by a short buttress lying between the posterior zygapophyses.

**Fig 34:**
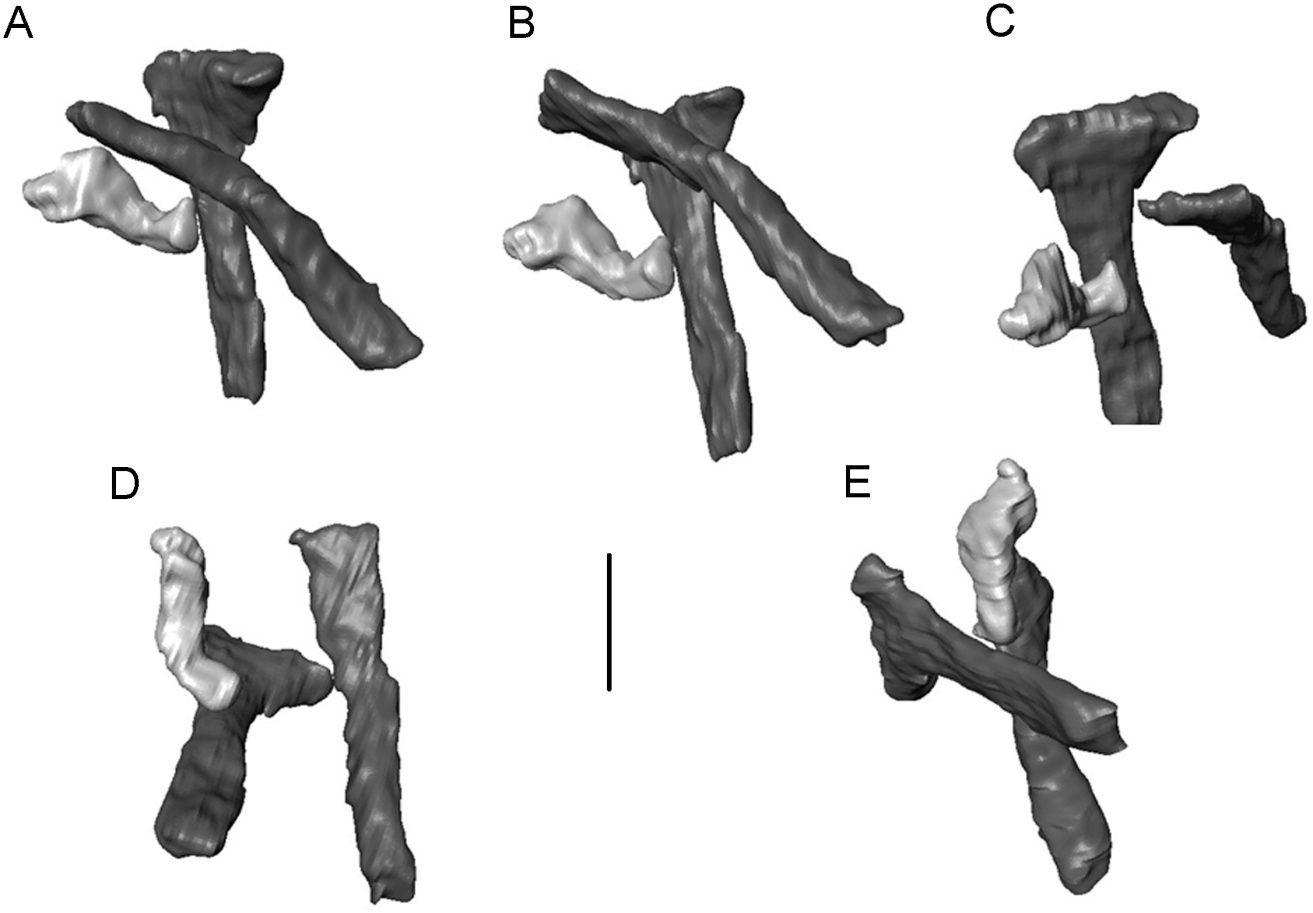
*Shirerpeton isajii* gen. et sp. nov., SBEI 2459, rib-like elements. Rib-like elements adjacent to vertebrae as segmented from μCT slice data in five views (original orientation uncertain). The dark grey elements are probably ribs or hyoid elements. The pale grey element is unidentified. Scale bar = 0.5 mm.

Lying adjacent to vertebra 6 are three bar-like bones (Figs 33 and 34). Two (dark grey in Fig 34) may be ribs or hyoid elements. One has a significantly expanded head and the other does not. The third bone (pale grey in Fig 34) is irregular in shape, with the two ends twisted in relation to one another. Whether this element is cranial or postcranial is uncertain.

**Fig 35:**
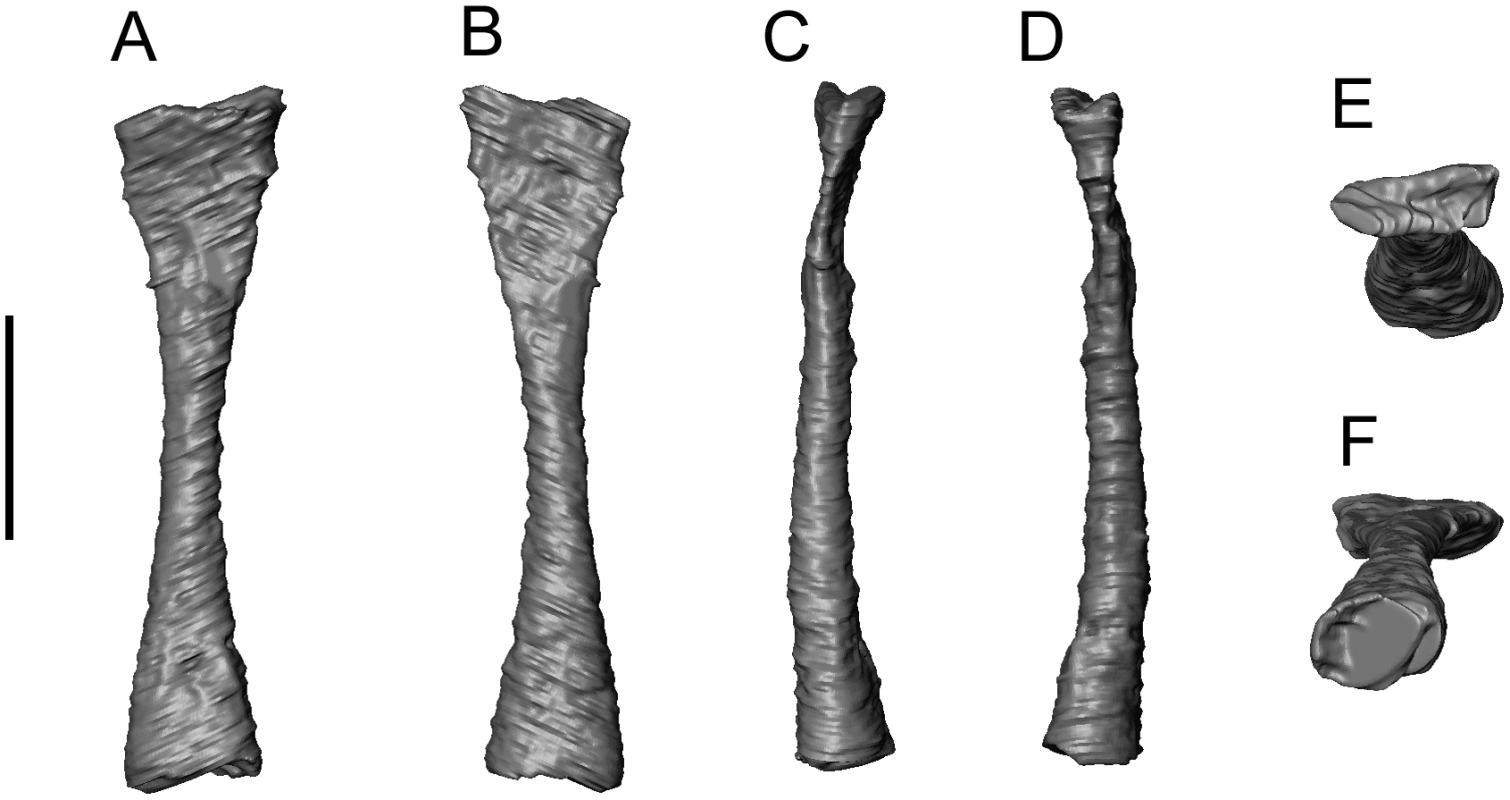
*Shirerpeton isajii* gen. et sp. nov., SBEI 2459, limb element. Limb element as segmented from μCT slice data in six views (original orientation uncertain). Scale bar = 1 mm.

One small limb bone lies between the anterior vertebral cluster and the frontal, adjacent to the two vertebrae at the edge of the block (Fig 35). It has a relatively long slender shaft and expanded proximal and distal ends. One end (shown as proximal in Fig 35A–35E) is anteroposteriorly compressed. The other end is rounded in section (Fig 35F). Given that the bone is shorter than three vertebral lengths, and very slender in midsection, it cannot (assuming similar proportions to *Celtedens ibericus*: [6, 8]) be either a femur or a humerus, nor can it be a metapodial as these elements are proportionally much shorter. From the size and shape, a tibia seems the most plausible interpretation.

Close to the otic capsule elements, there a series of small flattened, phalanx-like elements, the last of which is narrow and slightly curved, resembling the ungual phalanges of *Celtedens* ([8], SEE pers. obs.). Further phalanx-like elements are preserved behind the right parietal and adjacent to the sphenoid (see Fig 24A). We interpret these elements as the components of one or more digits.

## Phylogenetic analysis

Other than the new material described herein, there are currently four recognised albanerpetontid genera: *Anoualerpeton* ([20], Middle Jurassic, Europe, and Early Cretaceous, Morocco: 2 species), *Celtedens* ([6], Early Cretaceous, Europe: 2 species), *Wesserpeton* ([34], Early Cretaceous, Europe: 1 species), and *Albanerpeton* ([3], Early–Late Cretaceous, North America and Oligocene–Pliocene, Europe: 7 named species). There is also unnamed material from the Lower Cretaceous Spanish locality of Uña (Uña taxon [71]) and from the Palaeocene of Canada (Paskapoo taxon [30]). Extensive work by Gardner [7, 26–30, 72–73] and others [6, 8, 16–17, 20, 32–34] has led to a detailed understanding of many aspects of albanerpetontid morphology and a relatively stable phylogenetic framework for known taxa.

**Fig 36:**
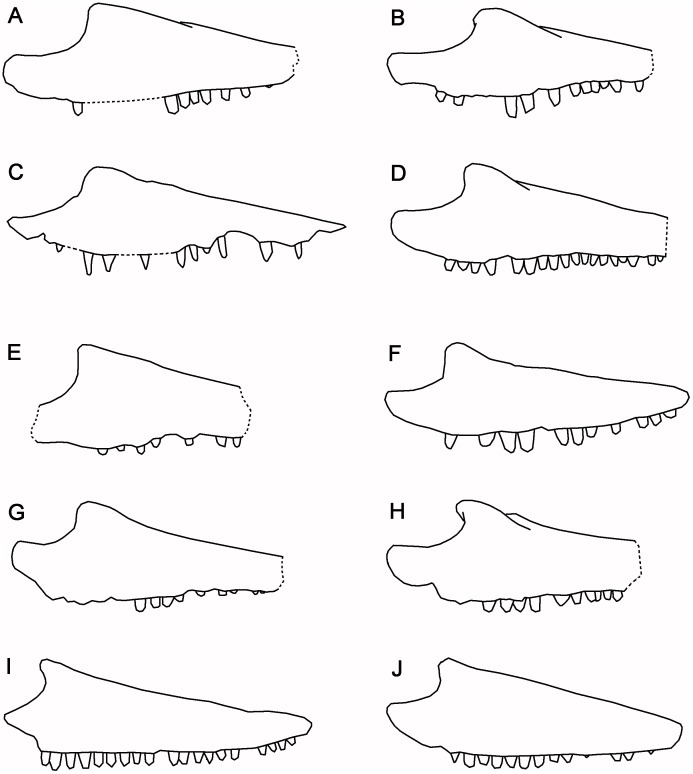
Lateral profiles of maxillae in albanerpetontids (not to scale). A, *Anoualerpeton priscum* (Middle Jurassic, UK); B, *An*. *unicum* (Early Cretaceous, Morocco); C, *Shirerpeton isajii* (Early Cretaceous, Japan); D, *Wesserpeton evansae* (Early Cretaceous, UK); E, *Albanerpeton arthridion* (Early Cretaceous, North America); F, *Albanerpeton gracile* (Late Cretaceous, North America); G, *Albanerpeton nexuosum* (Late Cretaceous, North America); H, *Albanerpeton galaktion* (Late Cretaceous, North America); I, *Albanerpeton inexpectatum* (Miocene, France); J, *Albanerpeton pannonicum* (Pliocene, Hungary). Outlines redrawn or reconstructed from: A-B [20]; C, original, SBEI 2459; D [34]; E [26]; F-H [29]; I [72]; J [16]. Images C-H have been "mirror-imaged" for ease of comparison.

**Fig 37:**
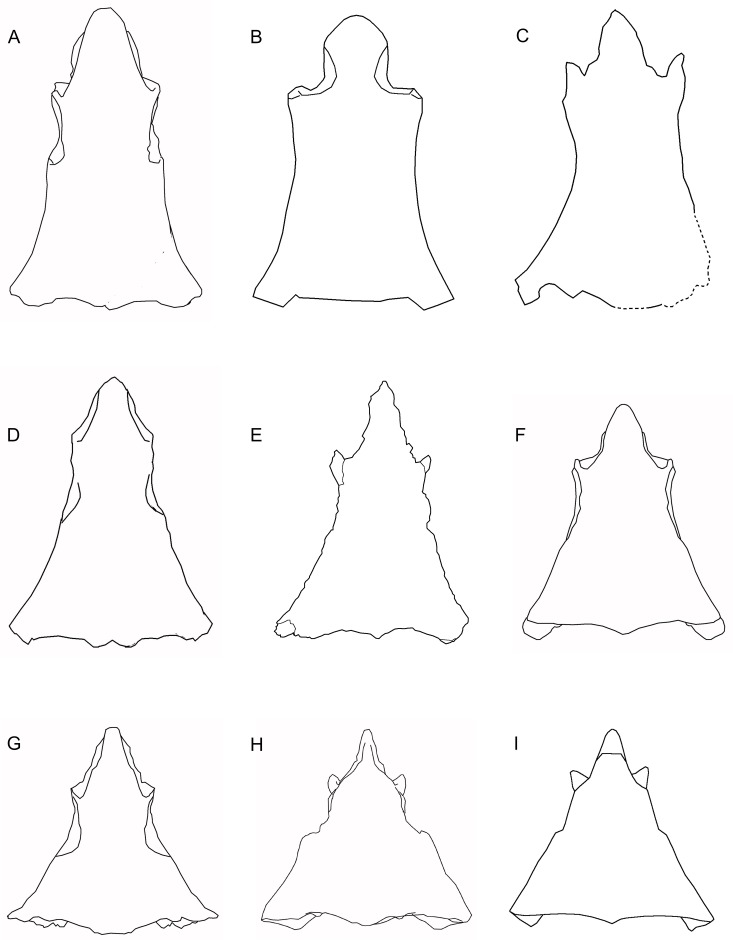
Comparison of frontal shape in albanerpetontids, scaled so that posterior widths are roughly equal. A, *Anoualerpeton unicum* (Early Cretaceous, Morocco); B, *Celtedens ibericus* (Early Cretaceous, Spain), based on [28]; C, cf. *Celtedens ibericus* (Early Cretaceous, Spain); D, *Wesserpeton evansae* (Early Cretaceous, UK); E, *Shirerpeton isajii* (Early Cretaceous, Japan); F, *Albanerpeton arthridion* (Early Cretaceous, North America); G, *Albanerpeton gracile* (Late Cretaceous, North America); H, *Albanerpeton pannonicum* (Pliocene, Hungary); I, *Albanerpeton inexpectatum* (Miocene, France). Figures redrawn from: A [20]; B [28]; C, original, MCCM-LH-15710; D [34]; E, original, SBEI 2459; F [26]; G [29]; H [16]; I [72].

Recently used data matrices [16, 34] coded 31 characters. Unfortunately, a majority of these (58%) relate to the premaxilla which is commonly preserved in microvertebrate deposits but is not preserved in the Japanese specimen. From the structure of the anterior margin of the nasal we infer that the nasal abutted the premaxilla medially but overlapped part of the premaxilla laterally. This does not match any of the states described [16] for character 4. Given that we lack the premaxilla of the Japanese albanerpetontid, we have coded this as unknown rather than create an additional autapomorphic state. Of the remaining, non-premaxillary, characters, we were able to score all but one for *Shirerpeton*. *Shirerpeton* has a long premaxillary process of the maxilla (ch.15:0) (Fig 36C), lacks a dorsally projecting process on the dentary behind the tooth row (ch.16:0), and has the maxillary tooth row extending anterior to the level of the facial process (ch.20:0), all regarded as primitive states [34]. We were unable to score the presence or absence of labial sculpture on the maxilla with confidence as the irregularities on the surface of the maxilla may be artefacts of damage or reconstruction (ch.17:?), but sculpture seems to be absent on the dentary. However, *Shirerpeton* shares the presence of a convexity of the maxillary and dentary alveolar margin (ch.18:1) with *Anoualerpeton* and *Albanerpeton nexuosum*, although it differs from both in having the convexity preceded by a concavity that renders the dentary very shallow anteriorly. It also resembles *Anoualerpeton* spp. and *Albanerpeton nexuosum* in having larger teeth anteriorly (ch.19:1). With an estimated snout-vent length of around 45 mm, *Shirerpeton* also shows the derived condition (ch.25:1), as do *A*. *arthridion* and *Wesserpeton*. On frontal characters (Fig 37E), *Shirerpeton* shows the derived state for frontal shape (ch.21:1), having a frontal that is approximately triangular rather than bell-shaped; a proportion of frontal length to posterior width that lies at the borderline between long (ch.22:0) and moderate (ch.22:1); a long derived internasal process (ch.23:1); a derived condition of the ventrolateral crests (ch.24:1); a derived condition for the anterior limit of the orbital margin (ch.28:1), located posterior to the midpoint of the anteroposterior long axis; a derived tapered form for the internasal process (ch.29:1); and a weak median keel along the ventral surface (ch.31:1). However, character 29 in the matrix of Sweetman and Gardner [34] stems from the original description of *Celtedens ibericus* as having a ‘bulbous’ nasal process [8, 74], a description based on interpretation of the articulated, but split, holotype specimen. An attributed specimen (MCCM-LH-15710) from the type locality, Las Hoyas, has the anterior end of the frontal disarticulated and clearly preserved (Fig 37B). It shows the nasal process to be a rounded taper rather than bulbous, and there is also greater separation between nasal and prefrontal facets than originally proposed. This raises doubts as to the original description.

Given that the most recent data matrices for albanerpetontids are heavily dependent on premaxillary characters [16, 34], it is important to begin to identify additional features that allow a more comprehensive characterisation of the skeleton as a whole. As a start, we have added three parietal characters. The first (ch.32) relates to the length of the postorbital wing compared to the frontoparietal suture width. The second (ch.33) relates to the extent to which sculpture does or does not cover the postorbital wing. The third (ch.34) relates to whether the occipital shelf is single or bifurcate.

**Fig 38:**
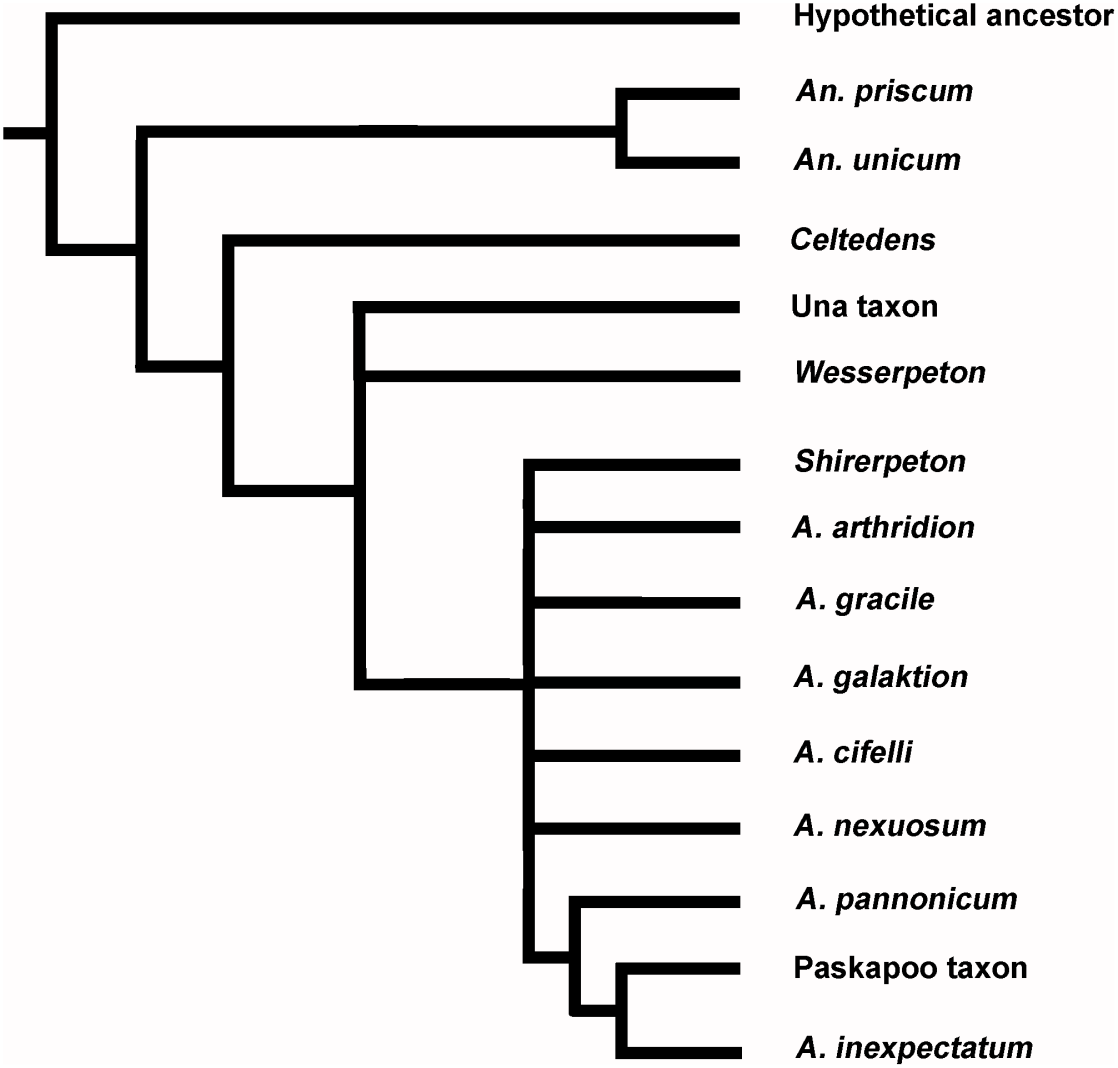
Strict consensus of 19 MPT’s from an analysis of the full matrix (NT search in TNT with Ratchet followed by a Traditional search of the resulting trees in RAM). Of the 19 MPT’s, 11% placed *Shirerpeton* as the sister taxon to a monophyletic *Albanerpeton*; 42% placed it as the sister taxon to *A*. *arthridion*; and 47% placed it crownward of *A*. *arthridion* (see Fig 39). An analysis with the limited matrix and without Hypothetical Ancestor yields trees with the same topology for the in-group taxa.

**Fig 39:**
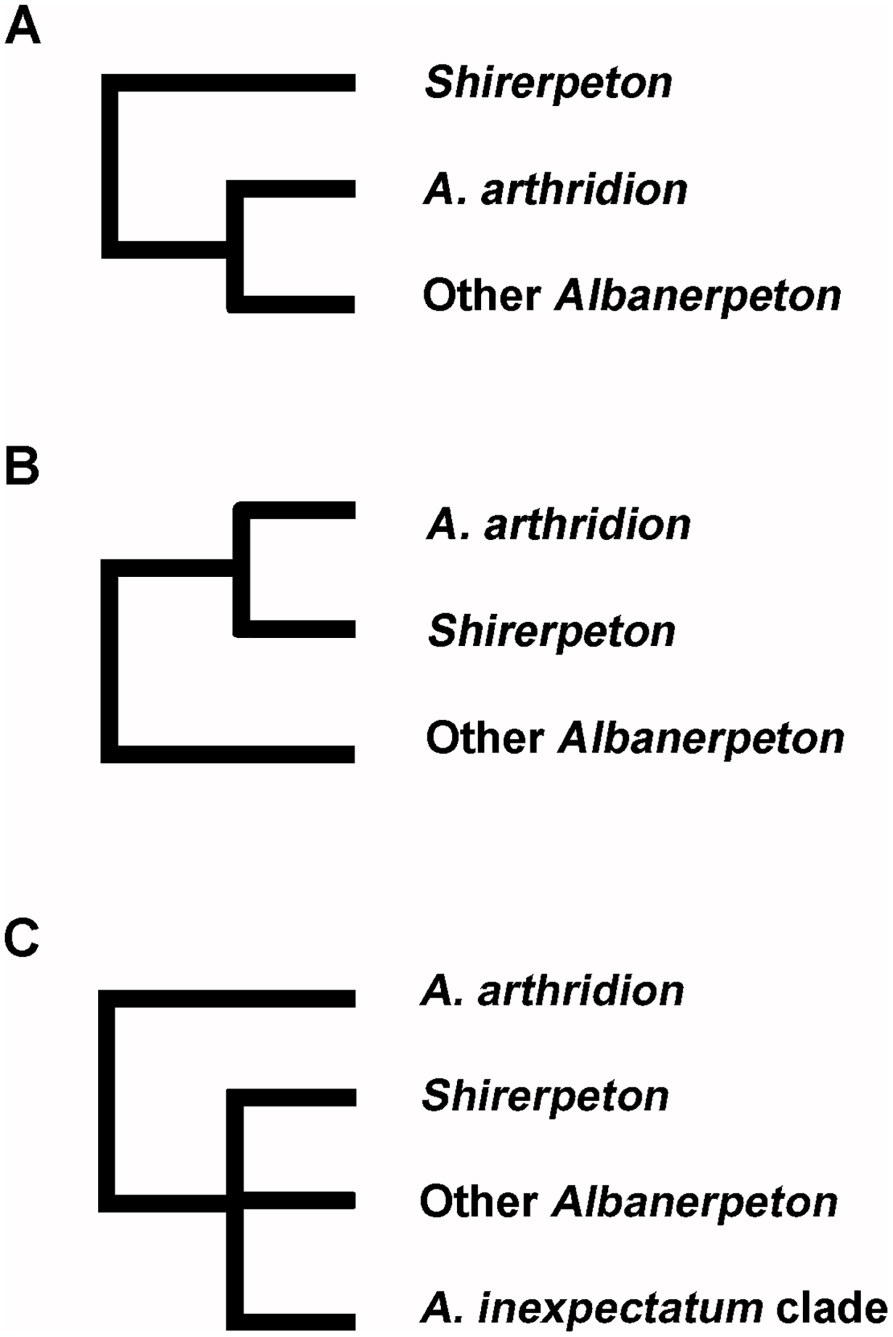
The three configurations recovered within the individual trees of the different analyses described in the text. In (C) the position of *Shirerpeton* is unstable with respect to individual *Albanerpeton* sp. The ‘*A*. *inexpectatum* clade’ consistently comprises *A*. *inexpectatum*, *A*. *pannonicum* and the Paskapoo taxon, with the variable addition of *A*. *nexuosum*.

**Fig 40:**
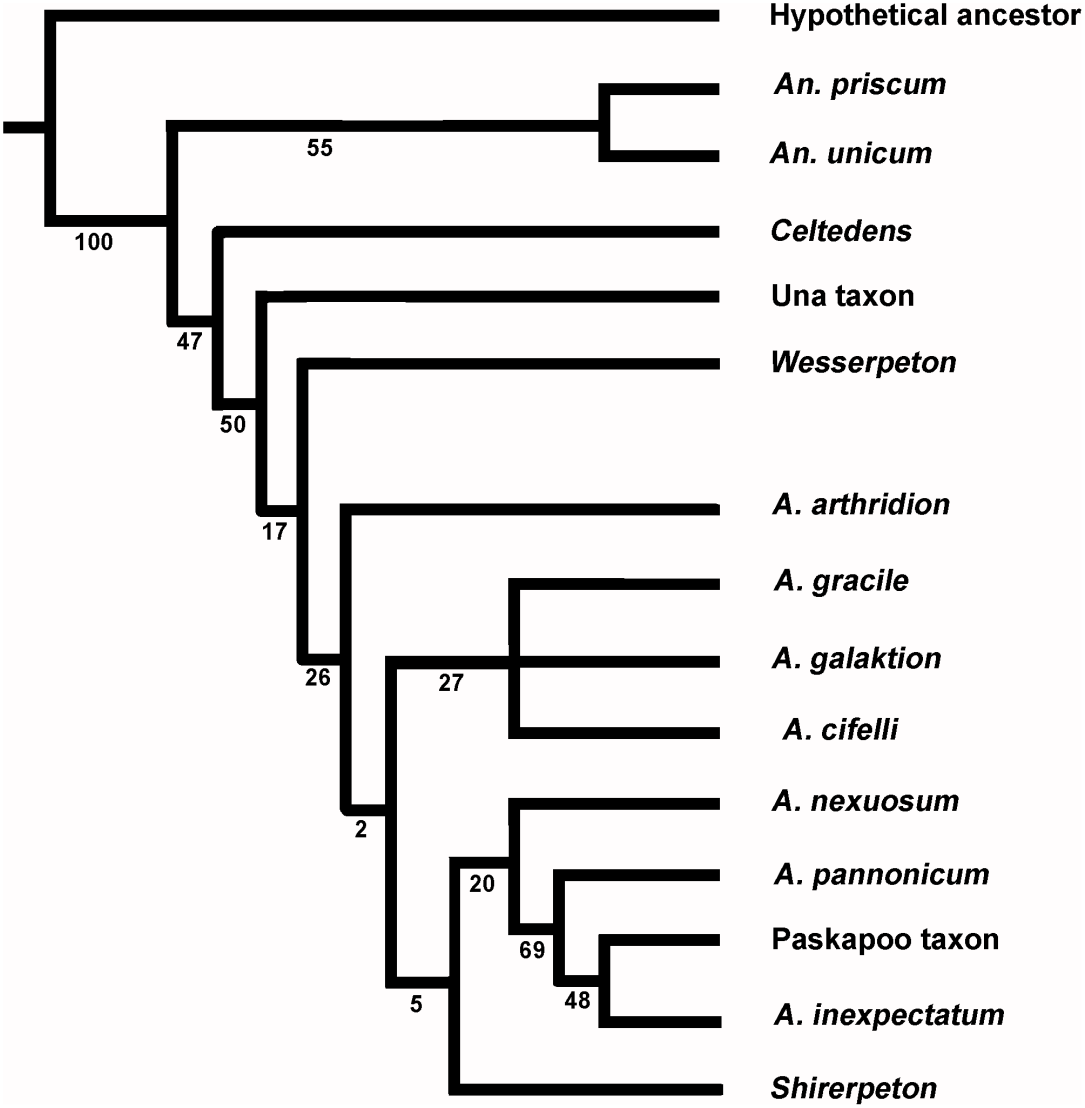
Bootstrap analysis (1000 replicates) using TNT (NT search + Ratchet [20 iterations]) of the full matrix, with the hypothetical ancestor as outgroup. The same analysis run using the limited matrix yielded the same topology, with minor differences in the Bootstrap values.

We ran a parsimony analysis, using the character matrix of Sweetman and Gardner [34] with our additional characters (S1 File). The first analysis was run with the Branch and Bound option of PAUP (for direct comparison with the analysis of Sweetman and Gardner [34]) (see S1–S3 Figs). A second analysis used TNT (version 1.5, [75]), New Technology search option with Ratchet (20 iterations) and 1000 Random addition sequences, followed by a scrutiny of the resulting trees using Traditional search. In each case, we ran the analysis (a) with and without the all-0 hypothetical ancestor of Sweetman and Gardner [34], rooting either with a basal node (PAUP) or with *Anoualerpeton priscum* (TNT), and also (b) with and without character 29 as the derived state is an uncertain autapomorphy of *Celtedens*. Despite the differences between analyses, and the programme used (PAUP, TNT), the resulting tree topologies were closely similar. Fig 38 shows the strict consensus of 19 trees from a TNT analysis of the full matrix (34 characters) and with the hypothetical ancestor as the outgroup (L = 51), but the tree topology was the same with character 29 deleted, and with the hypothetical ancestor replaced with *Anoualerpeton*. *Shirerpeton* is placed in an unresolved polytomy with *Albanerpeton* species. The same analyses run with PAUP (Branch and Bound), yielded 53 trees (L = 57) of which the Strict Consensus was identical to that in Fig 38. Examination of the individual trees from both analyses identified three alternative positions for *Shirerpeton* (Fig 39): i) as sister group to *Albanerpeton* as a whole; ii) as sister taxon to *A*. *arthridion*; or iii) in variable positions crownward of *A*. *arthridion*. The first of these arrangements was the rarest (15%), with the remaining trees showing the second or third topology in roughly equal numbers. However, a Bootstrap analysis (2000 replicates) using TNT (NT + Ratchet [20 iterations]) yielded a more resolved tree (Fig 40) resembling that of Sweetman and Gardner [34] in recovering a ‘robust-snouted clade’ comprising *A*. *nexuosus*, *A*. *pannonicum*, *A*. *inexpectatum*, and the Paskapoo taxon, and a ‘gracile-snouted clade’ comprising *A*. *gracile*, *A*. *galaktion*, and *A*. *cifellii*, with *A*. *arthridion* as the sister taxon to both clades, and *Wesserpeton*, the Uña taxon, *Celtedens*, and *Anoualerpeton* as successive outgroups. *Shirerpeton* is nested within *Albanerpeton* as the sister taxon of the ‘robust-snouted clade’, but Bootstrap support values for all nodes are very low and only the ‘robust-snouted clade’ survived a decay analysis beyond one step. Moreover, when synapomorphies are followed on the tree, none unequivocally supports the placement of *Shirerpeton* as the sister taxon of the ‘robust-snouted clade’ and only two frontal characters (ch.22, 28) support the placement of *Shirerpeton* crownward of *Wesserpeton*.

In summary, the results of the phylogenetic analyses consistently support the placement of *Shirerpeton* crownward of *Anoualerpeton* and *Celtedens*, and provide more limited support for a position crownward of the Uña taxon and *Wesserpeton*, in a closer relationship with species currently referred to the genus *Albanerpeton*. This is discussed further below.

## Discussion

### Novel morphology

SBEI 2459 preserves the disarticulated, but associated, bones of a single small individual. Although a few surface elements have suffered damage, individual bones are largely uncrushed and the μCT scans have revealed significant new information on albanerpetontid cranial morphology. The specimen preserves several elements that were previously either unknown or incompletely known from other albanerpetontid taxa–notably the nasal, septomaxilla, prefrontal, lacrimal, jugal, quadrate, squamosal, parietal, supraoccipital, sphenoid, and other braincase components. Moreover, some of the unidentified elements suggest that the albanerpetontid skull may have retained other elements subsequently lost in crown Lissamphibia (e.g. the supratemporal).

**Fig 41:**
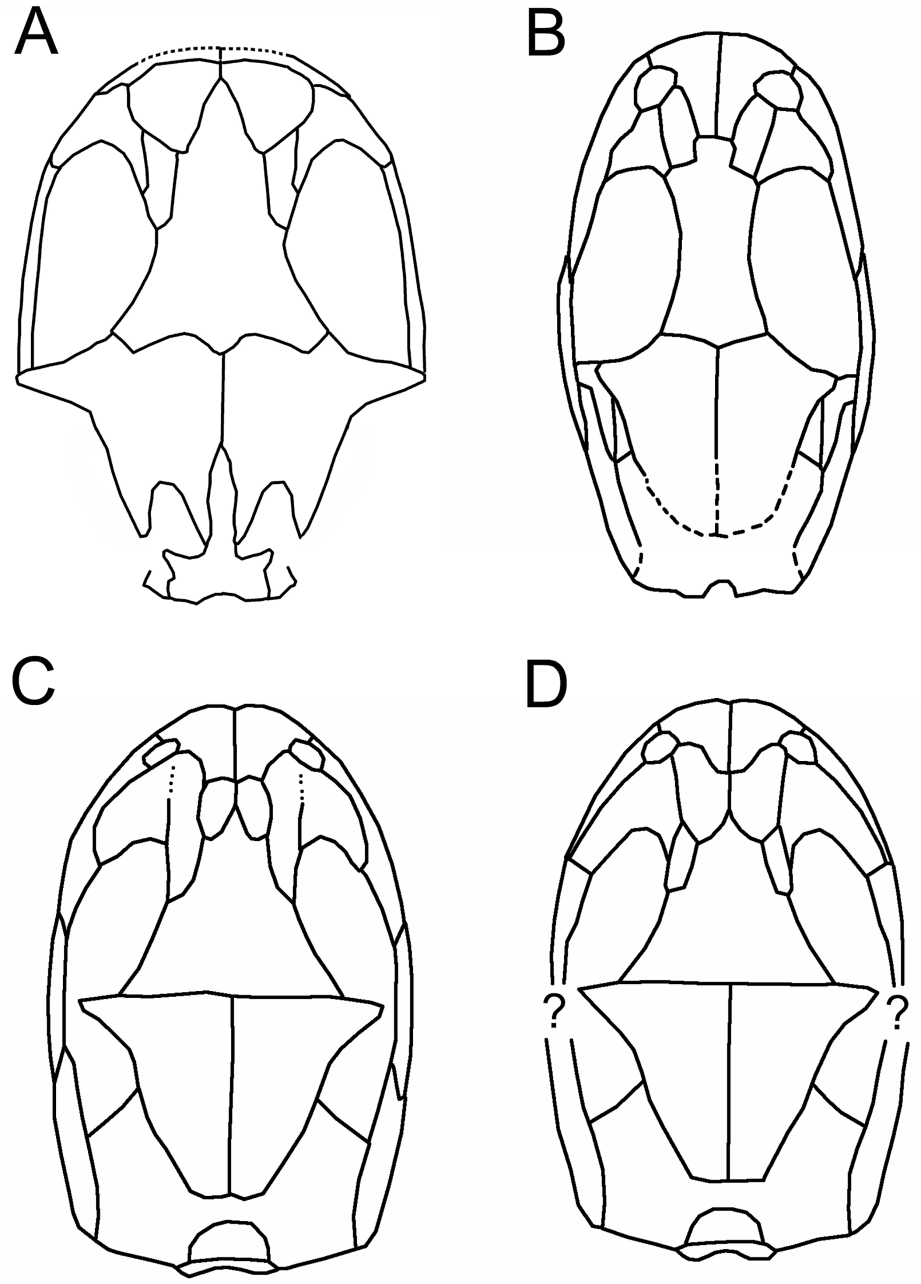
Skull roofing bones in albanerpetontids (not to scale). A, *Shirerpeton isajii*; B, *Celtedens ibericus*; C, *Albanerpeton inexpectatum*; D, *Albanerpeton pannonicum*. B-D, redrawn from [16], but with B based on [8].

The albanerpetontid rostrum was first reconstructed by Estes & Hoffstetter [3] on the basis of disarticulated, but well-preserved, bones of the Miocene species, *A*. *inexpectatum*. The nasals were not preserved but, based on surrounding bones, the authors reconstructed them as small oval elements lying between the frontal, premaxillae, and prefrontals, but excluded from the narial margins. The prefrontal was inferred to be fused to the lacrimal, with some uncertainty as to the limits of each bone. Gardner [72–73] broadly agreed with this interpretation. However, on the basis of articulated Early Cretaceous material of *Celtedens ibericus* from Las Hoyas, Spain, McGowan [8] concluded that small ovoid nasals ‘did not appear to exist’, and re-interpreted Estes and Hoffstetter’s [3] fused prefrontals as elongate, slender nasals. In McGowan’s interpretation, the posterolateral facet on the frontal accommodated the lacrimal, not a prefrontal, with the nasal articulating further anteriorly and entering the narial margin. However, the Las Hoyas specimens are flattened and are usually split between part and counterpart, complicating interpretation (SEE pers. obs.). The rostral morphology of *Celtedens ibericus* is therefore uncertain. More recently, three-dimensionally preserved and partly articulated material of *A*. *pannonicum* [16] clarified the relationships of the nasals, prefrontals, lacrimals, and frontal to one another, at least in Neogene *Albanerpeton*. SBEI 2459 supports the interpretations of Estes & Hoffstetter [3] and of Venczel & Gardner [16], rather than McGowan [8]. It demonstrates the presence of a discrete prefrontal that articulated with the frontal medially and lacrimal laterally, and of a separate nasal. As in *A*. *pannonicum*, the nasals of *Shirerpeton* were relatively large. Our reconstruction (Fig 3) suggests that the nasals were separated for most of their length by the internasal process of the frontal, but entered the narial margins and contacted the lacrimals to exclude the prefrontals from those margins. In shape (relative angles of the dorsal and lateral processes, position and depth of the lateral groove), the lacrimal of *Shirerpeton* more closely resembles that of *A*. *inexpectatum* than *A*. *pannonicum*, allowing for the non-fusion of the prefrontal (Fig 41).

A small septomaxilla has also been revealed in *Shirerpeton*. This bone is variably present in members of all extant lissamphibian clades [68] but has not previously been described in an albanerpetontid.

The quadrate of *Albanerpeton inexpectatum* was mentioned briefly by previous authors [2, 3], and figured at small scale. In *Shirerpeton*, the facets suggest that the quadrate had a firm articulation with the squamosal and pterygoid. SBEI 2459 has also confirmed the presence of discrete jugals in albanerpetontids, as in *Eocaecilia* [76] but not extant lissamphibians. However, unlike previous reconstructions [8] that present the jugal as a straight bar between the maxilla and suspensorium, in *Shirerpeton* the jugal has a posterodorsal curvature and may have had a ligamentous attachment with the skull roof. There are no obvious posteroventral facets or processes to indicate how (or whether) the jugal contacted the suspensorium.

**Fig 42:**
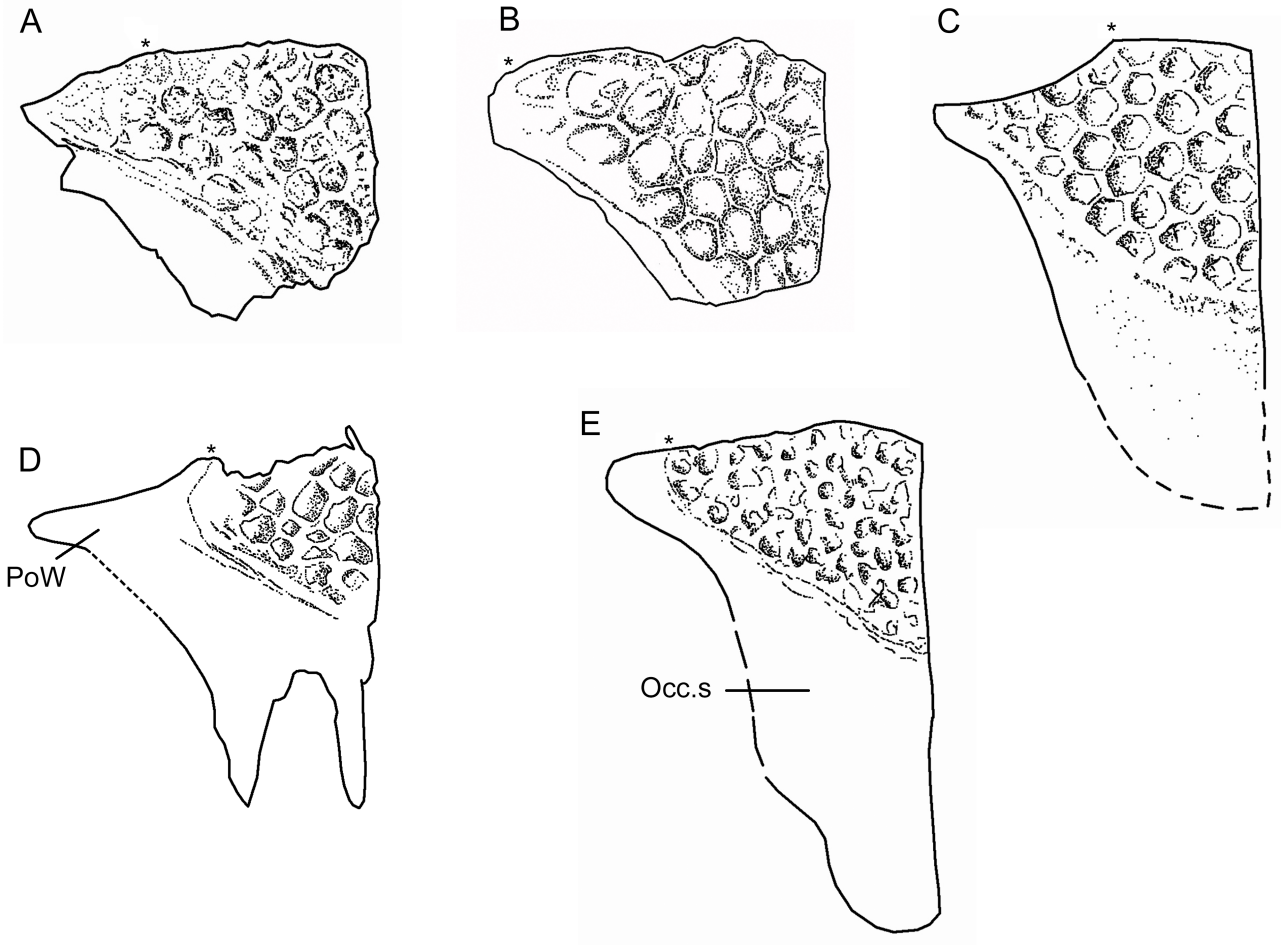
Parietal morphology in albanerpetontids (not to scale). A, *Anoualerpeton priscum* (Middle Jurassic, UK); B, c.f. *Wesserpeton evansae* (Early Cretaceous, UK); C, *Celtedens ibericus* (Early Cretaceous, Spain); D, *Shirerpeton isajii*, Early Cretaceous, Japan); E, *Albanerpeton inexpectatum*, Miocene, France). In each case, the small asterisk marks the lateral limit of the frontal facet, lateral to which is the postorbital wing. Abbreviations: Occ.s, occipital shelf; PoW, postorbital wing. Figures redrawn from: A [23] (mirror-imaged for comparison); B, original, NHMUK PV R 36956; C [6]; D, original, SBEI 2459; E [3].

Although the albanerpetontid parietal is probably as taxonomically distinctive in its shape and ornamentation as the frontal, it has only rarely been preserved and/or described (Fig 42). Estes & Hoffstetter [3] provided images of the almost complete parietals of *A*. *inexpectatum*, showing the bones to be paired, with an acuminate lateral postorbital wing, a sculptured triangular central area, and unsculptured occipital shelves (Fig 42E). The parietals of *Celtedens ibericus* [6,8] have a similar overall shape, although the details are less clearly preserved (Fig 42C). For the remaining taxa, however, the parietal is either unknown or fragmentary. McGowan [23] figured the anterior portion of a right parietal from the Middle Jurassic of Britain, referable to *Anoualerpeton priscum* (Fig 42A), and Wiechmann [71] figured fragments of two left bones of ‘*Celtedens*’ *guimarotae* (not reproduced here as the figures are difficult to interpret). Sweetman & Gardner [34] did not describe the parietal of *Wesserpeton*, but a partial (anterior) left bone was recovered from the type deposit (Natural History Museum, London, NHMUK PV R 36956, Fig 42B). The Tetori holotype specimen (Figs 3 and 42D) is therefore exceptional in preserving both parietals almost in their entirety. They differ from the known parietals of other taxa in three major respects: the postorbital wing is relatively longer; there is a more limited lateral extension of the sculpture so that it does not encroach on the postorbital wing; and the occipital shelf is shorter and bifurcated. This bifurcation is clearest on the right bone where part of a vertebra extends between the posteromedial and posterolateral rami. The detailed three-dimensional preservation of the parietals in *Shirerpeton* also demonstrates, for the first time, how they articulated with the supraoccipital and epipterygoids.

Perhaps the greatest differences in morphology between *Shirerpeton* and the European Neogene taxa are found in the braincase. Estes and Hoffstetter [3] figured stereopairs of an isolated three-dimensional braincase of *A*. *inexpectatum*, but these images are too small to yield any detail. Moreover, this specimen, formerly in the collections of the Museum National d’Histoire Naturelle, Paris, is now apparently lost [13]. Nonetheless, it is clear from the images and the description that the component elements of the braincase were fully conjoined. A second three-dimensional braincase, attributed to the Pliocene *A*. *pannonicum*, has also been described [13]. Again, the components are co-ossified, leaving the homologies of some regions uncertain. Although Maddin et al. [13] reported the presence of an ossified tectum synoticum in *A*. *pannonicum*, with an anterior projection, the lack of sutures left the authors unable to determine whether this ossification represented a supraoccipital. In *Shirerpeton*, however, it is clear that the supraoccipital is an independent ossification. Neither of the Neogene braincases has an anteromedian process as long as that of *Shirerpeton*, but *A*. *pannonicum* preserves a short process that may be incomplete. No living lissamphibian has a discrete supraoccipital ossification, although De Beer [70] suggested that it might be included within the os basale of caecilians. A supraoccipital ossification is also absent in putative stem-lissamphibians like the temnospondyl *Doleserpeton* [77], but it is present in the lepospondyl *Brachydectes* [78] where the element is remarkably similar to that of *Shirerpeton*.

**Fig 43:**
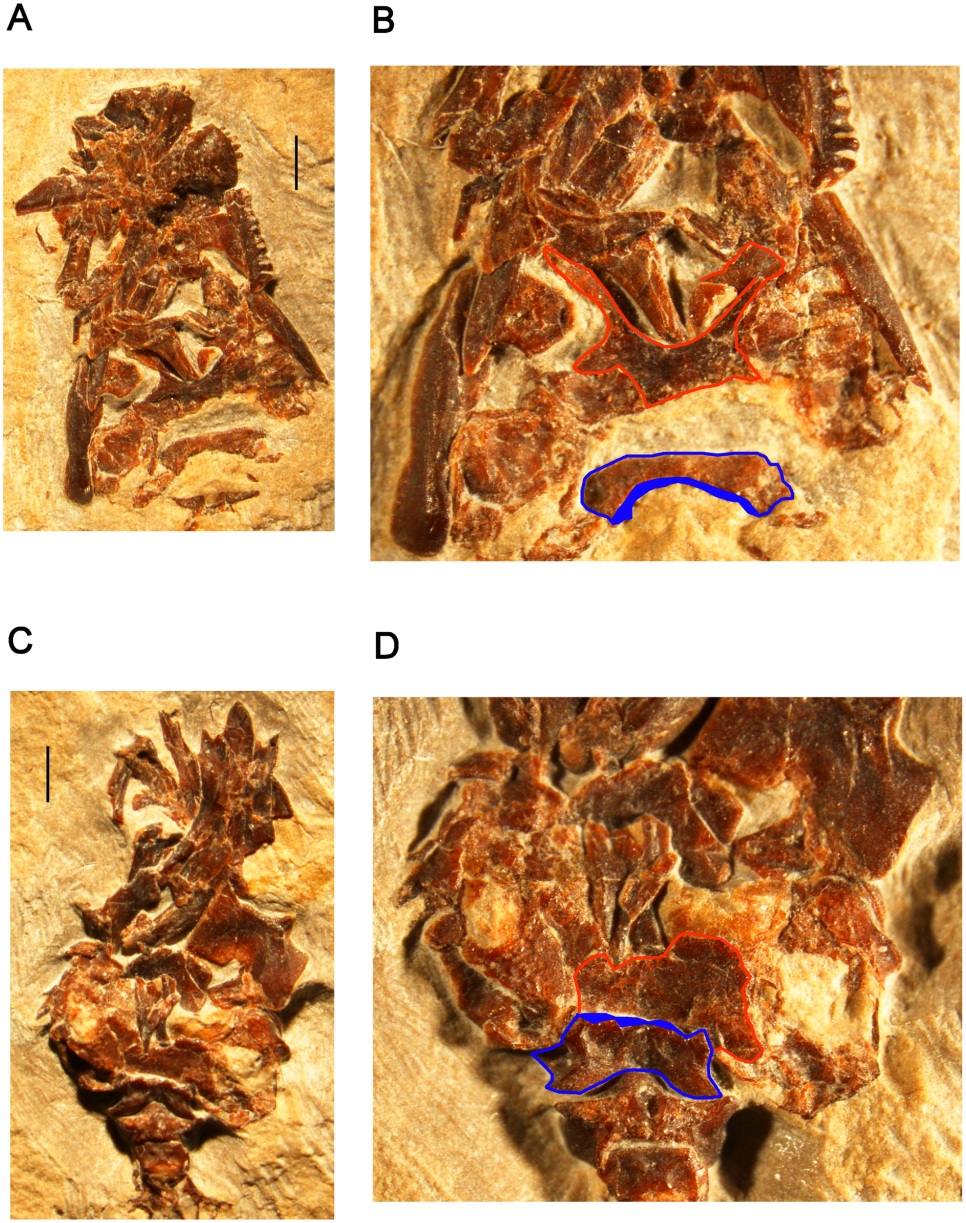
*Celtedens ibericus*, Las Hoyas specimen, Museo de las Ciencias de Castilla-La-Mancha, MCCM-LH-15710, digital photograph of skull of part and counterpart. A,B MCCM-LH-15710b, with A, whole skull and B, enlargement of braincase region; C,D, MCCM-LH-15710a, with C, whole skull and D, enlargement of braincase region. In B and D, blue outlined element is the exoccipital plate, and red outlined element is the sphenoid. Scale bar = 1 mm.

Ventrally, *Shirerpeton* lacks a median basioccipital but the paired exoccipital plates presumably had the same embryonic origin. Whether the notochord was enclosed by the plates, or overlay them in the midline is unclear. Maddin et al. [13] postulated the presence of a transverse suture between anterior and posterior parts of the braincase floor in both *A*. *inexpectatum* and *Celtedens ibericus*. This is also the case in *Shirerpeton*, although our examination of the key Las Hoyas specimen (Museo de las Ciencias de Castilla-La-Mancha, Cuenca, Spain, MCCM-LH-15710) showed that labelled suture in this specimen [13] is actually a break within the exoccipital plate, with the other part of this element preserved on the counterpart (blue outlines in Fig 43). Moreover, the more anterior element interpreted by [13] as the dorsum of the braincase, is actually a sphenoid like that of *Shirerpeton* (red outlines in Fig 43). In *A*. *pannonicum*, Maddin et al. [13] concluded that the ventral floor of the neurocranium was ‘composed largely of the parasphenoid’ with ‘minor contributions at the posterolateral corners from the otic capsules’. This is very different from the condition in *Shirerpeton* where the floor is clearly formed by the exoccipital plates. The configuration of the parasphenoid remains unknown. In living frogs and salamanders, the exoccipital plates do not generally meet in the midline, but this may not apply to the solidly fused os basale of caecilians [70].

Both previous accounts of the albanerpetontid braincase [3, 13] recorded the presence of one or more hypoglossal foramina piercing the exoccipital. Foramina are also present in the Tetori specimen but we were unable to follow a clear path through the bone and therefore the identification of these foramina remains uncertain.

The albanerpetontid palate remains an enigma. One small element in the Tetori skull has been tentatively identified as a possible ectopterygoid or palatine but the other ventral components of the skull (parasphenoid, pterygoids, vomers, elongated median hyoid element) are not preserved. It seems likely that these ventral skull components were displaced either laterally or anteriorly and were on the part of the block that was lost before the dorsal elements were exposed.

### Systematic position of *Shirerpeton* within Albanerpetontidae

As most albanerpetontid material is disarticulated, the elements most frequently used in diagnosis are the premaxilla and the distinctive median frontal [6, 30, 34, 74]. The oldest, and apparently more plesiomorphic, taxa (*Anoualerpeton*, *Celtedens*) have a frontal that has been described as ‘hour-glass’ or ‘bell-shaped’ [6, 8, 73–74], in that the bone is significantly longer than it is wide, and the lateral orbital borders are concave (Fig 37A–37C). The anterior limit of the frontal orbital margin (posterior edge of the prefrontal facet) lies anterior to the mid-point of the anteroposterior long axis. The second frontal morphotype, which is considered to characterise the derived genus *Albanerpeton* [28, 30, 34, 72], has lateral borders that are straight (or nearly so) and a posterior margin that is significantly wider than the anterior one (Fig 37F–37I). The anterior margin of the orbit lies at or posterior to the mid-point of the long axis of the frontal. The frontal length and width are roughly equal, so that the bone is strongly triangular, a shape enhanced by an acuminate internasal process that may be flanked by smaller anterolateral processes. However, length/width proportions differ considerably between early species like *A*. *arthridion* and derived Neogene species such as *A*. *inexpectatum* and *A*. *pannonicum*. The Barremian genus *Wesserpeton* [34] has a frontal that is intermediate in morphology between the two main types, in that it is triangular and acuminate like that of Neogene *Albanerpeton*, but is somewhat longer in relation to its posterior width, and the anterior margin of the orbit is roughly level with the mid-point of the frontal long axis (Fig 37D).

As reconstructed, the frontal of *Shirerpeton* has a length/posterior width proportion of 1.23, as compared to 0.87–1 for *A*. *inexpectatum* [72]; 0.88–0.95 for *A*. *pannonicum* [16]; ~1.2 in *A*. *gracile*, *A*. *nexuosum*, *A*. *galaktion* [29, 30], and *A*. *arthridion* [26]; 1.0–1.3 for *Wesserpeton* [34]; 1.3–1.4 for *Celtedens ibericus* (depending on the reconstruction used); and 1.57 for *Anoualerpeton unicum* and *An*. *priscum* [20]. Of all species currently known, the reconstructed frontal shape and proportions of *Shirerpeton* are most like those of the North American *A*. *arthridion* (Fig 37F), the oldest (uppermost Aptian or lower Albian) species referred to *Albanerpeton*, although the internasal process is longer in the Japanese taxon.

Estes & Hoffstetter [3] figured the parietals of *A*. *inexpectatum* which, although slightly damaged posteriorly, clearly differ in shape from those of the Japanese taxon in that the unsculptured posterior occipital shelf is long and wide rather than bifurcate. There is also a difference in proportion in relation to the width of the fronto-parietal contact and the length of the postorbital wing. In *A*. *inexpectatum*, the postorbital wing is relatively short (Fig 42E) and the sculpture extends almost to its tip. In *Shirerpeton*, the wing forms almost half the anterior width of the bone, and is unsculptured (Fig 42D). The parietals are also preserved in *Celtedens ibericus* [6, 8], but are crushed. The postorbital wings of *Celtedens*, as reconstructed (Fig 42C), are proportionally longer than those of *Shirerpeton* (more than 58% parietal width), but bear sculpture as in *A*. *inexpectatum*. The same condition is seen in the few fragmentary parietals preserved for the Middle Jurassic *Anoualerpeton priscum* [23], Fig 42A), and in the parietal fragment attributed to *Wesserpeton evansae* (Fig 42B). Unfortunately, the tip of the wing is missing in both UK taxa, but (from the angle of the converging margins) it does not appear to have been long. A long, unsculptured postorbital wing may therefore be a diagnostic feature of *Shirerpeton*, but without comparable elements from *A*. *arthridion* and members of the ‘gracile-snouted’ clade, this remains tentative.

Of the jaw elements, comparison between albanerpetontid species has focused mainly on the premaxilla, an element that remains unknown in *Shirerpeton*. As listed [34], maxillary characters have been limited to the presence or absence of labial sculpture; the position of the anteriormost tooth relative to point of maximum indentation of the narial margin; and the presence or absence of an expansion of the labial dental margin in association with enlarged teeth. *Shirerpeton* shows the primitive state in the first two of these, and shares the heterodonty and expanded dental margin with species of *Anoualerpeton* and *Albanerpeton nexuosum*. However, as Fig 36 demonstrates, there are other maxillary features that vary between species. These include the length and shape (rounded or pointed) of the anterior premaxillary process; the degree of concavity of the anterior narial margin; and the shape of the dorsal facial process in terms of angulation and profile. The difficulty is that few complete maxillae have been described and most sample sizes are small. On the basis of existing material, the maxilla of *Shirerpeton* (Fig 36C) appears to be distinct in combining heterodonty with a relatively short, pointed premaxillary process, little curvature of the narial margin, and a low, rounded facial process. Even fewer dentary characters have been identified, other than those relating to labial sculpture and heterodonty, and the presence of a dorsal process posterior to the tooth row, currently reported only in *Albanerpeton inexpectatum* [34]. Again, *Shirerpeton* most closely resembles *Anoualerpeton* and *Albanerpeton nexuosum* in having enlarged anterior teeth supported by an expansion of the labial dental margin, although it differs in the greater degree of sinuosity of the margin due to the concave-convex-concave profile. The dentary of *Shirerpeton* also appears to differ in two other ways. The anterior limit of the prearticular facet seems to be in line with the rear of the tooth row and posterior to the opening of the Meckelian fossa, whereas it extends forward below the tooth row to a point roughly in line with the opening of the Meckelian fossa in most other taxa where this region is known. Secondly, in many albanerpetontids (including *A*. *nexuosum* and the two species of *Anoualerpeton*), the subdental shelf rises steeply at the posterior end of the tooth row such that the posterior tooth loci are significantly smaller (25% or less) than those close to the symphysis. In *Shirerpeton*, the subdental shelf is shallower and, apart from the group of enlarged teeth, there is less of a difference in size between anterior and posterior teeth. Thus the combined characters of the maxilla and dentary in *Shirerpeton* allow it to be distinguished from other albanerpetontid taxa known only from dental elements. This includes elements from the Upper Cretaceous of Central Asia attributed to ‘*Nukusurus*’ [31], which lack heterodonty and have an anteriorly extended prearticular facet and very small posterior teeth.

Taken together with the morphological features, the results of the phylogenetic analyses provide some support for the placement of the Japanese albanerpetontid crownward of the European *Wesserpeton* and the Uña taxon (sensu [34]). Although the Strict Consensus (PAUP, TNT) is partially unresolved (Fig 38), only a minority of individual trees placed the Japanese taxon as the sister group to a monophyletic *Albanerpeton*. Of the remaining trees, roughly half placed the Japanese albanerpetontid as the sister taxon to *A*. *arthridion*, which it resembles in frontal morphology and small size, but not dentary morphology (proportionally deeper anteriorly in *A*. *arthridion*, without the strongly sinuous alveolar margin [26]). The other half of the individual trees placed the Japanese taxon crownward of *A*. *arthridion*, but of unstable position in relation to other included species. The Bootstrap analysis (Fig 40) placed *Shirerpeton* on the stem of the ‘robust-snouted’ clade. These topologies raise the question as to whether the Japanese species should be attributed to *Albanerpeton*.

The type species of *Albanerpeton* is the European Miocene *A*. *inexpectatum* [3]. Although the first albanerpetontids (represented by dentaries) recovered in North America were placed in the genus *Prosiren* [1], the designated holotype of *Prosiren* was a vertebra later shown to belong to a salamander [12]. Based on their unique structure (tooth and symphysial morphology) the dentaries originally attributed to *Prosiren* [1] were re-assigned to the distinctive European genus *Albanerpeton* [12], as was all subsequent North American material. In the interim, however, there have been both temporal range extensions (Middle Jurassic to Pliocene), and a recognition of generic diversity among European Mesozoic albanerpetontids (*Anoualerpeton*, *Celtedens*, *Wesserpeton*). Compared to these relatively short-lived genera, *Albanerpeton* as currently defined extends from the? Aptian-Albian to the late Pliocene, an interval of more than 100 Myr. Given the difference in frontal shape between, for example, *A*. *arthridion* and *A*. *inexpectatum* (Fig 37), it seems likely that there is greater taxonomic diversity within *Albanerpeton* than is currently recognised by the nomenclature. This applies particularly to the earliest North American species, and the unstable placement of the Japanese taxon in relation to these species (especially *A*. *arthridion*) may partly reflect this. Resolution of this problem would be advanced by the recovery and description of additional elements (especially parietals) for the North American species, as current diagnoses and phylogenies rely too heavily on premaxillary characters.

Based on the above discussion, we consider it preferable to give the new Japanese albanerpetontid separate generic status. This reflects i) its distinctive character combination; ii) the striking differences in braincase morphology between *Shirerpeton* and the Neogene *Albanerpeton* species; iii) the unstable phylogenetic position of the Japanese taxon in relation to existing *Albanerpeton* species; iv) a lack of overlap of preserved elements, resulting in more than 50% missing data when coded into existing matrices (mainly due to a lack of premaxillae); v) the very limited knowledge of albanerpetontid parietal and braincase morphology; and vi) the fragmentary nature of most of the North American material. The separate taxonomic identity also facilitates an objective discussion of albanerpetontid biogeography.

### Systematic position of Albanerpetontidae among tetrapods

Discussion of the systematic position of albanerpetontids amongst tetrapods is complicated by the lack of agreement on the evolutionary relationships and monophyly of lissamphibians [14, 79–80], and by a lack of information on the early history of each major group. *Triadobatrachus* [77, 81–82] and *Czatkobatrachus* [83–85] provide some information on early frog evolution, but it is very incomplete, as is that for early caudates [86–89]. *Eocaecilia* is more completely known [5, 76] but, until the recent description of the Triassic *Chinlestegophis* [78] as a possible stem-caecilian, it had been isolated in time. The recovery of new material pertaining to the early history and divergence of lissamphibians is clearly crucial.

Albanerpetontids have the potential to shed light on early lissamphibian morphology, but in the current state of phylogenetic flux, a detailed analysis of albanerpetontid relationships to other tetrapods is beyond the scope of the current paper. Existing data matrices differ quite strikingly in their coding of the same taxa (e.g. compare the codings in [5, 14] vs [79]) and yield conflicting results, even when the same approaches are used. Nonetheless, the presence of epipterygoids and a separate supraoccipital in albanerpetontids argues against a nested position within Batrachia (as a caudate sister group) as some previous authors have suggested [1–5]. Moreover, although there is no question as to the monophyly of Albanerpetontidae, the differences in braincase anatomy between *Shirerpeton* and the Neogene species of *Albanerpeton* provide a clear argument against representing Albanerpetontidae as a single operational unit in phylogenetic analyses as has often been the case [5, 10, 79, 90]. *Shirerpeton* is separated from *A*. *inexpectatum* and *A*. *pannonicum* by around 100 million years, but is itself separated by a similar time interval from the likely Permo-Triassic ancestral albanerpetontid of which nothing is known.

### Biogeography

Whatever the precise relationship between albanerpetontids and the three living lissamphibian clades, their last common ancestor must have lived in the Paleozoic. This leaves a long hiatus before the first recorded appearance of albanerpetontids in the Middle Jurassic, limiting discussion as to their place of origin. The Jurassic record is currently restricted to Europe (France, England, Portugal [21, 22, 71, 91]) and North Africa (Morocco [18]). Most Early Cretaceous records are also European (Britain [34, 92], France [93], Italy [94], Spain [6], Sweden [95]) and the group is represented in Europe for much of the Late Cretaceous (Spain, France, Hungary, Romania, [96]), as well as intervals of the Palaeogene (Oligocene, Germany [17]) and Neogene (France, Germany, Czech Republic, Austria, Hungary, Italy [17]). Albanerpetontids survived in Europe until at least the late Pliocene (Italy [24]) where they are associated with the remains of extant reptile and amphibian genera.

There are no confirmed records of albanerpetontids in North America before the late Early Cretaceous and this coincides with the first occurrence of specimens currently referred to *Albanerpeton* [17, 26]. Gardner’s phylogenetic work [16–17, 28, 34] recognises *A*. *arthridion* from the Cloverley and Antlers formations of Oklahoma and Texas, USA, as the sister taxon to all other (younger) *Albanerpeton* species and, *Shirerpeton* aside, our analyses support this placement. The precise age of the oldest horizons bearing *A*. *arthridion* are uncertain, as radiometric dates are lacking, but they are generally given as uppermost Aptian or lowermost Albian [26, 97]. *Albanerpeton* then apparently radiated in North America through the Late Cretaceous (at least seven attributed species), with records in every stage from the Cenomanian to the Maastrichtian [17]. Specimens from the middle to late Palaeocene of Canada [17] show that the group survived the end-Cretaceous extinction in North America, but there are currently no younger records. In Europe, the genus *Albanerpeton* is first reported from the Late Cretaceous (Campanian) of France, but it is important to note that many Late Cretaceous European albanerpetontid records are based on fragmentary specimens that are really diagnostic only at family level. The two named European species, the Miocene *A*. *inexpectatum* and Pliocene *A*. *pannonicum*, fall within a single well-supported clade [17] with material from the Palaeocene Paskapoo Formation of Canada.

Up until recently, therefore, the distribution pattern was consistent with the hypothesis that the genus *Albanerpeton* originated in North America, around the mid-Cretaceous, and subsequently spread to Europe. However, as Gardner & Bőhme [17] observed, the expanding record of albanerpetontids in Europe weakens that argument, as does the absence of any confirmed record of albanerpetontids in North America prior to the latest Aptian/earliest Albian. This apparent absence may be an artefact of the fossil record, there being no Middle Jurassic or Berriasian-Barremian microvertebrate faunas known from North America. However, albanerpetontids are also conspicuously absent from the Upper Jurassic Morrison Formation despite the existence of several rich microvertebrate localities where they would be expected to occur (based on other components of the faunal assemblage). It is therefore possible that albanerpetontids first reached North America during the Early Cretaceous but, if so, from where [26]?

One possibility is that derived albanerpetontids spread to North America from Europe via a North Atlantic route, as suggested for some mammals [98–99]. This hypothesis has received support from the description of the European *Wesserpeton* and its placement as a sister taxon to *Albanerpeton*. Sweetman & Gardner [34] suggested that the split between the ancestral lineages of these two genera might have occurred in western Eurasia, with a *Wesserpeton*-like ancestor subsequently migrating into North America via Europe and giving rise to *Albanerpeton*. They further proposed (based on *Wesserpeton*) that ‘acquisition of a triangular (frontal) shape preceded shortening of the bone and that this evolutionary trait was acquired in western Eurasia some time prior to the Barremian’ [34].

An alternative route into western North America, where records are concentrated, would be from eastern Asia via the Beringian land bridge. This route has been proposed for several groups of dinosaurs, lizards, mammals and choristoderes that appeared in North America toward the end of the Early Cretaceous [100–103]. However, some authors [17, 31–32] have argued against that scenario for albanerpetontids, on the basis that the first record in North America (*A*. *arthridion*) both predated the opening of the Beringian land bridge and predated any record of albanerpetontids in Asia. The first objection is moot as there is uncertainty both on the precise dating of the relevant American deposits (e.g. [97]) and of the opening of the land bridge (e.g. [101–104]). Nonetheless, up until now, the second objection stood, because the earliest confirmed Asian records of albanerpetontids were from the early Cenomanian of Uzbekistan [31–33,105–106]). To explain their apparently late appearance in Asia, Gardner & Averianov [31] and Skutschas [32] favoured a mid-Cretaceous dispersal of albanerpetontids from North America into Asia. Clearly, the recovery of a relatively derived albanerpetontid in the Early Cretaceous of Japan is not consistent with that scenario. It is plausible that the ancestral *Wesserpeton*-like stock proposed by Sweetman & Gardner [34] could have spread eastwards rather than (or as well as) westwards from western Eurasia, although the potential for interchange may have been limited by the opening of the Turgai Straits between Asia and western Eurasia from the late Middle Jurassic onward [31, 107]. Moreover, none of the Middle Jurassic assemblages of Russia and Central Asia has yielded conclusive evidence of albanerpetontids [33, 108]. This is surprising given the general resemblance of these assemblages to the contemporaneous faunas of Britain where albanerpetontids do occur [22]. An albanerpetontid frontal was reported [106] from the Middle Jurassic Balabansai Formation of Kirghizia, but this observation cannot be confirmed as the specimen was neither figured nor described and cannot be located ([31].

It remains possible, of course, that the apparent absence of the group at some localities is an artefact of local environmental or taphonomic factors [109]. Albanerpetontids have yet to be recovered from the fossil rich Chinese Daohugou Beds (Jurassic) or the Jehol Biota (Early Cretaceous), yet the discovery of *Shirerpeton* in Japan, then part of the eastern Asian mainland, renders it highly likely that the group was present in China and other parts of Asia at this time. Recent isotope analysis of reptile bone suggests that the Early Cretaceous Yixian Formation, at least, was deposited under similar (cool) climatic conditions (mean air temperature of ~10°C, [110]) to those of the Kuwajima Formation, but perhaps the lacustrine environments that have been richly sampled in China were less suitable for small terrestrial albanerpetontids. Most Mesozoic albanerpetontids are found in lowland estuarine or swampy environments [17]. The Japanese Kuwajima Formation conforms to this pattern (swampy inland wetland), but albanerpetontid remains are extremely rare (to date, 3 out of 2459 catalogued specimens) and it seems likely that they entered the deposit from further afield, possibly in the gut of a predator.

One further chapter in the history of Asian albanerpetontids is currently being written. Daza et al. [111] recently described a collection of lizard fossils from an amber deposit in Myanmar (Burma) dated to Albian-Cenomanian age. The image of one very small specimen, tentatively identified [111] as an early chamaeleonid, was re-identified by one of us (SEE) as an albanerpetontid. μCT has confirmed this identification and work on that specimen is ongoing. It provides an important temporal link between the Japanese specimen and the younger material from Uzbekistan, and offers support for the idea that albanerpetontids were well established and relatively widespread across at least eastern and south-eastern Asia during the Cretaceous.

## Conclusions

The recovery of the new Japanese specimens sheds new light on albanerpetontid morphology and biogeography, but raises as many questions as it resolves. There is clearly much more to discover about these enigmatic little tetrapods, in terms of their morphology, relationships, and evolutionary history. Recent discoveries have demonstrated that the group had a more extensive temporal and geographical distribution in Asia than previously understood. Awareness of this among researchers may lead to further discoveries.

## Supporting information

S1 Fig Strict consensus of 53 trees run in PAUP using the full matrix, with the hypothetical ancestor as outgroup. This tree topology matches that recovered from the TNT analysis shown in Fig 34. Of the 53 individual MPTs, 15% placed *Shirerpeton* as the sister taxon to a monophyletic *Albanerpeton*; 45% placed it as the sister taxon to *A*. *arthridion*; and 40% placed it crownward of *A*. *arthridion*.
(TIF)

S2 Fig PAUP analysis of the full matrix run without the hypothetical ancestor. Left, Bootstrap Analysis; right, 70% Majority Rule Tree of 53 individual trees.
(TIF)

S3 Fig Bootstrap analysis using PAUP of the limited matrix. There is less resolution with respect to *Wesserpeton* and the Uña taxon.
(TIF)

S1 File Characters and data matrix used in the phylogenetic analysis. (DOCX)
