# Supplementary figures and images for: The first record of albanerpetontid amphibians (Amphibia: Albanerpetontidae) from East Asia
Source: PLoS One. 2018 Jan 3;13(1):e0189767. doi: 10.1371/journal.pone.0189767 (PMC5752013; doi:10.1371/journal.pone.0189767)

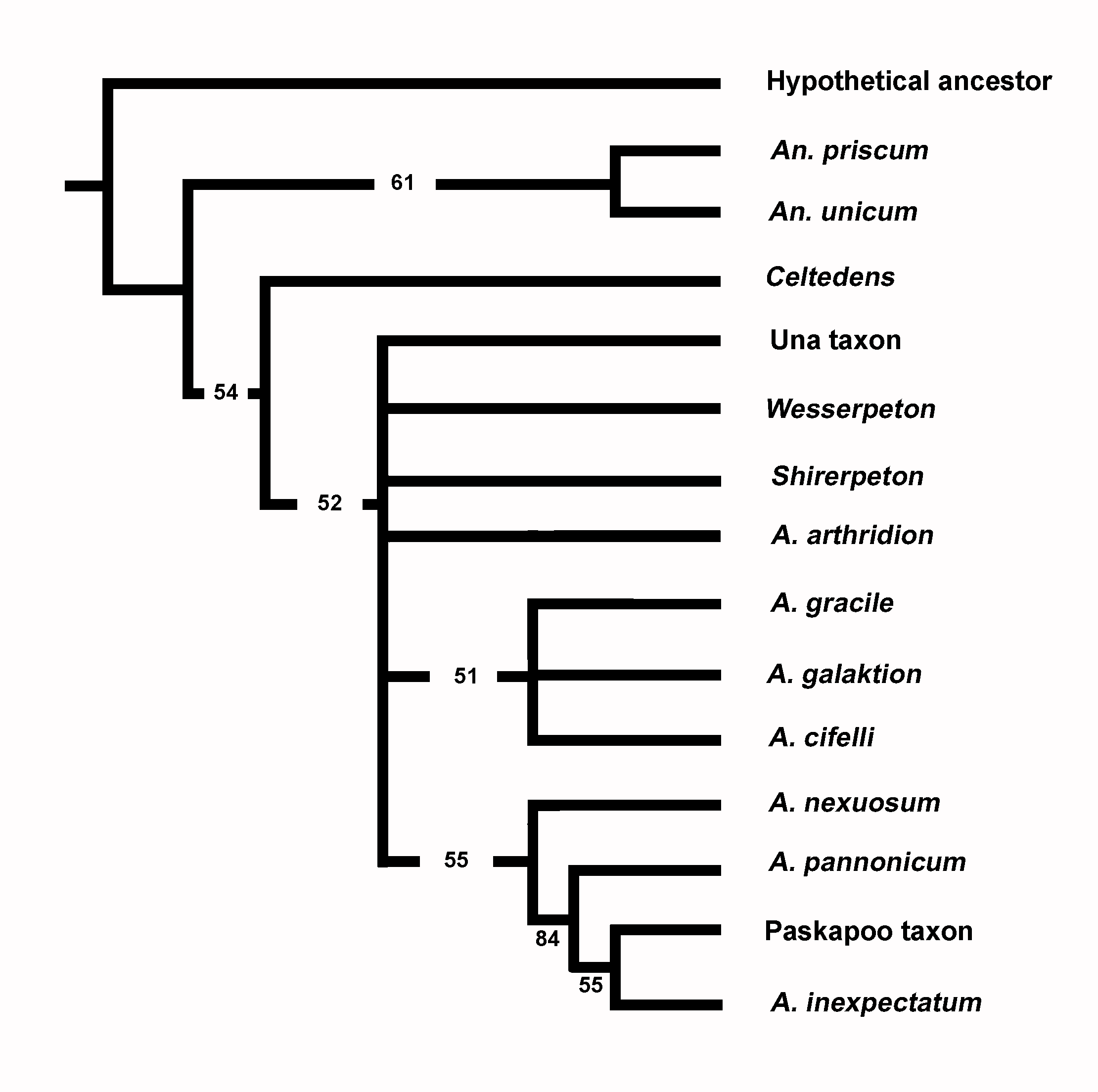

Supplement: S1 Fig — This tree topology matches that recovered from the TNT analysis shown in Fig 34. Of the 53 individual MPTs, 15% placed Shirerpeton as the sister taxon to a monophyletic Albanerpeton; 45% placed it as the sister taxon to A. arthridion; and 40% placed it crownward of A. arthridion. (TIF) [file pone.0189767.s001.tif]

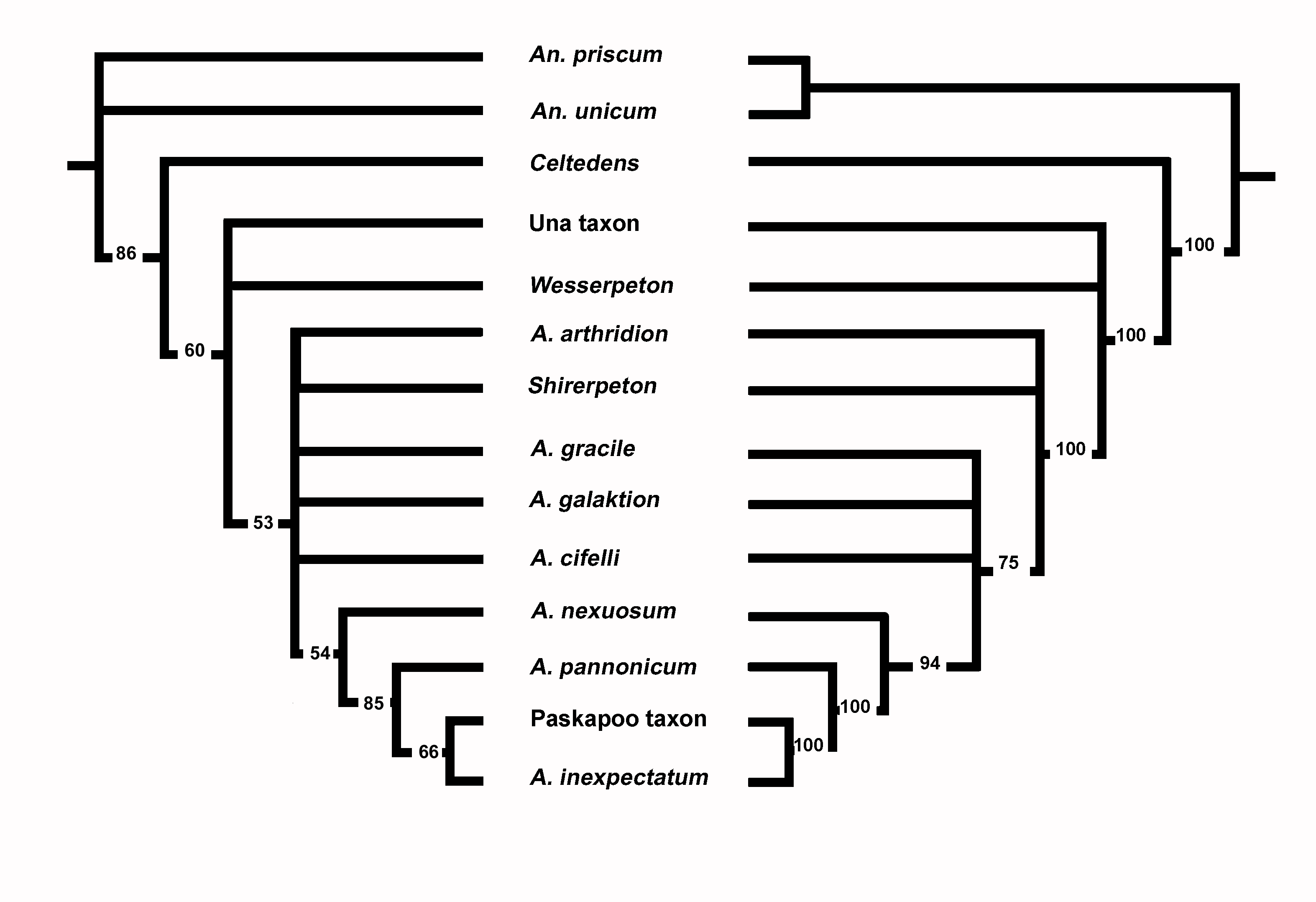

Supplement: S2 Fig — Left, Bootstrap Analysis; right, 70% Majority Rule Tree of 53 individual trees. (TIF) [file pone.0189767.s002.tif]

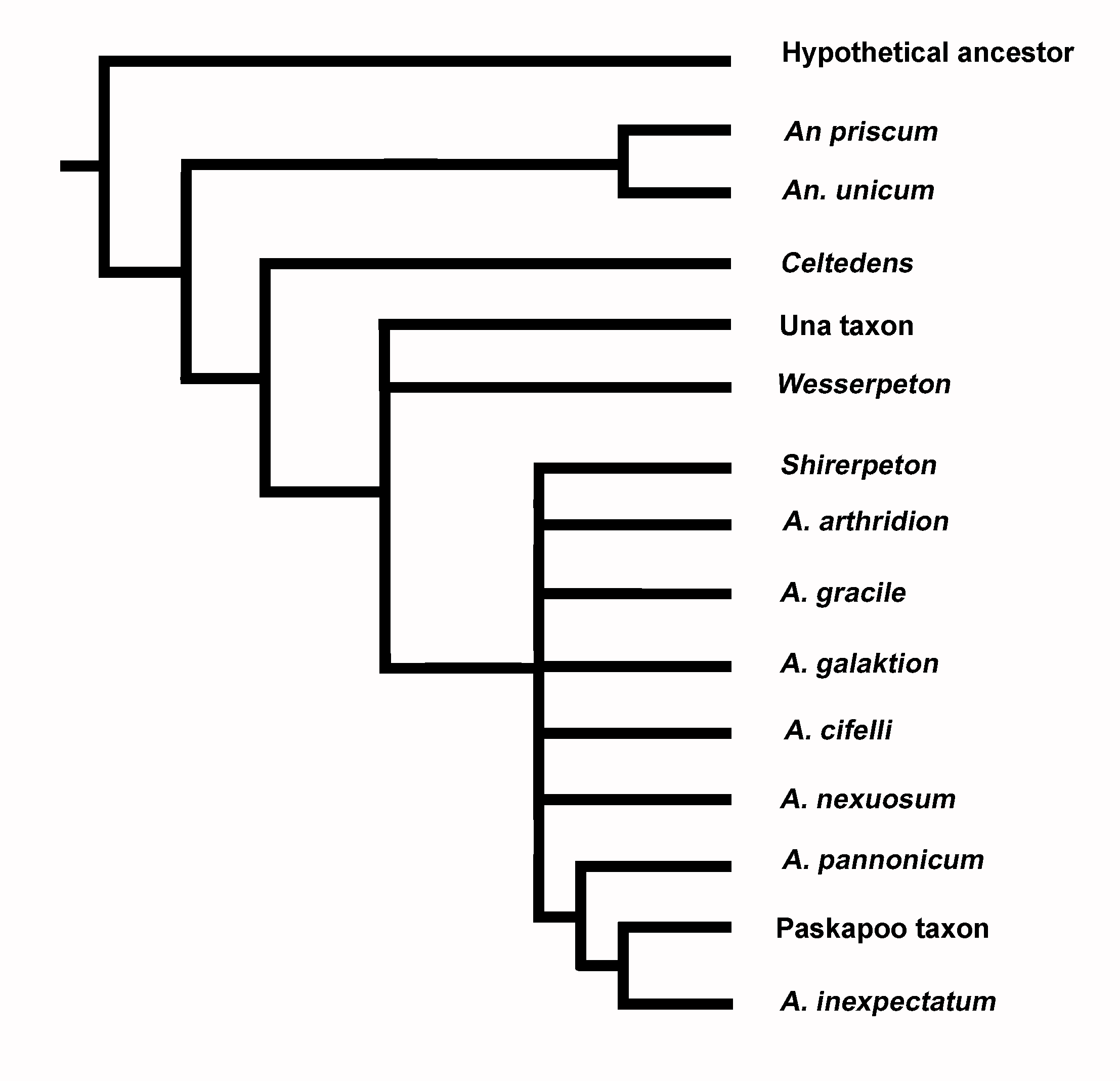

Supplement: S3 Fig — There is less resolution with respect to Wesserpeton and the Uña taxon. (TIF) [file pone.0189767.s003.tif]
