# Supplementary material for: The first record of albanerpetontid amphibians (Amphibia: Albanerpetontidae) from East Asia
Source: PLoS One. 2018 Jan 3;13(1):e0189767. doi: 10.1371/journal.pone.0189767 (PMC5752013; doi:10.1371/journal.pone.0189767)
Supplement: S1 File — (DOCX) [file pone.0189767.s004.docx]

**S1 file. Characters and data matrix used in the phylogenetic analysis**

Characters 1-31 are taken from Sweetman and Gardner (2013), characters 32-34 are new to this analysis

1. Build of premaxilla: 0, gracile; 1, robust.

2. Ratio of height of premaxillary pars dorsalis versus width across suprapalatal pit: 0, “high”, ratio greater than about 1.55: 1, “low”, ratio less than about 1.55.

3. Inter−premaxillary contact: 0, sutured medially (i.e., paired); 1, fused medially in some individuals.

4. Premaxillary−nasal contact: 0, premaxillary pars dorsalis minimally overlaps and abuts against or weakly sutured with anterior end of nasal; 1, premaxillary pars dorsalis minimally overlaps and strongly sutured with anterior end of nasal; 2, anterior end of nasal fits into lingual facet on premaxillary pars dorsalis and braced ventrolaterally by expanded dorsal end of lateral internal strut.

5. Boss on premaxilla: 0, present; 1, absent.

6. Relative size of premaxillary boss, if present: 0, covers about dorsal one−quarter to one−third of pars dorsalis; 1, covers about dor sal one−half of pars dorsalis.

7. Distribution of labial ornament on large premaxillae: 0, restricted to dorsal part of pars dorsalis; 1, covers entire face of pars dorsalis.

8. Pattern of premaxillary labial ornament: 0, discontinuous, anastomosing ridges and irregular pits; 1, continuous ridges defining polygonal pits; 2, pustulate.

9. Vertical position of suprapalatal pit on pars dorsalis: 0, “high”, with ventral edge of pit well above dorsal face of pars palatinum; 1, “low”, with ventral edge of pit just above or, more typically, continuous with dorsal face of pars palatinum

10. Size of suprapalatal pit relative to lingual surface area of pars dorsalis: 0, “small”, about 1%; 1, “moderate”, about 4–15%; 2, “large”, about 20–25%.

11. Outline of suprapalatal pit: 0, oval; 1, triangular or slit−like.

12. Form of dorsal process on lingual edge of maxillary process: 0, low, isolated ridge; 1, high flange, continuous labially with base of lateral internal strut.

13. Form of vomerine process on premaxilla: 0, prominent; 1, weak.

14. Diameter of palatal foramen in premaxilla relative to diameter of base of medial teeth on bone: 0, “small”, foramen diameter tooth diameter; 1, “large”, foramen diameter > about one and one−third tooth diameter.

15. Length of premaxillary lateral process on maxilla relative to height of process at base: 0, “long”, length > height; 1, “short”, length  height.

16. Dorsally projecting process on dentary immediately behind tooth row: 0, absent; 1, present.

17. Labial ornament on large maxilla and dentary: 0, absent; 1, present.

18. Labial or lingual profile of occlusal margin of maxilla and den tary: 0, essentially straight; 1, strongly convex or angular, with apex adjacent to tallest teeth.

19. Size heterodonty of teeth on maxilla and dentary: 0, weakly heterodont anteriorly; 1, strongly heterodont anteriorly.

20. Position of anterior end of maxillary tooth row relative to point of maximum indentation along leading edge of nasal process: 0, anterior to; 1, approximately in line.

21. Dorsal or ventral outline of fused frontals: 0, approximately rectangular− or bell−shaped; 1, approximately triangular.

22. Ratio of midline length of fused frontals versus width across posterior edge of bone, between lateral edges of ventrolateral crests, in large specimens: 0, “long”, ratio more than about 1.2; 1, “moderate”, ratio between about 1.2 and 1.1; 2, “short”, ratio equal to or less than about 1.0. (Sh=1.21 – so coded as 0 and 1)

23. Proportions of internasal process on fused frontals: 0, “short”, length . width; 1, “long”, length > width.

24. Form of ventrolateral crest on large, fused frontals: 0, narrow and convex ventrally to bevelled ventrolaterally in transverse view; 1, narrow and triangular in transverse view, with ventral face flat to shallowly concave; 2, wide and triangular in tranverse view, with ventral face deeply concave.

25. Estimated maximum snout−pelvic length: 0, “large”, > about 50 mm; 1, “small”, < about 45 mm.

26. Direction faced by suprapalatal pit in pars dorsalis of premaxilla: 0, laterolingually; 1, lingually.

27. Path followed by canal through pars palatinum in premaxilla, between dorsal and ventral openings of palatal foramen: 0, dorso−laterally−ventromedially; 1, vertically.

28. Position in frontals of anterior end of orbital margin relative to anteroposterior midpoint of frontals: 0, in front of; 1, in line with or behind.

29. Dorsal or ventral outline of internasal process on frontals: 0, tapered anteriorly; 1, bulbous.

30. Suprapalatal pit variably divided: 0, undivided; 1, divided in about one−third or more of specimens.

31. Flattened ventromedian keel extending along posterior two thirds of fused frontals: 0. absent; 1, present.

32. Postorbital wing of parietal length: 0, equal or longer than width of frontoparietal suture; 1, shorter than width of frontoparietal suture. (new)

33. Sculpture extent on postorbital wing of parietal: 0, sculpture extends on to wing; 1, wing unsculptured. (new)

34. Posterior process of parietal: 0, single; 1, double (new)

Matrix

Codings for all taxa except *Shirerpeton* taken from Sweetman and Gardner (2012)with codings for characters 32-34 added where possible from published descriptions or observations

Hypothetical ancestor 00000 00000 00000 00000 00000 00000 000?

*Anoualerpeton priscus*  0?0?? ?0?00 00000 00110 00110 00?00 0?0?

*Anoualerpeton unicus* 01000 00000 00?00 ??110 00000 00000 0???

*Celtedens ibericus* 000?? ?0?00 0???? 0000? 00000 1?010 0000

*Albanerpeton arthridion*  00000 00000 0000? 00000 11001 11100 0???

*Albanerpeton gracilis* 00000 00011 10000 00001 11000 11100 0???

*Albanerpeton galaktion* 00000 00012 10010 ?0000 11010 11100 0???

*Albanerpeton cifellii* 00020 00011 1000? ????? ????? 11?0? ????

*Albanerpeton nexuosus* 11110 10111 01001 00110 11110 11100 0???

*Albanerpeton inexpectatum* 11111 91211 00001 11001 12120 11101 0110

*Albanerpeton pannonicus* 11110 00011 00001 00001 12121 11101 1??0

Paskapoo species 11111 91011 00101 00001 1?101 11101 0???

*Wesserpeton evansae* 0a010 000aa 00?0a 0000a 10001 1a101 010?

Uña albanerpetontid 000?0 00?00 0???? ????? 10000 1?00? 0???

*Shirerpeton isaji* ????? ????? ????0 0?110 1a111 ??10? 1011
